# Supplementary material for: HaploCart: Human mtDNA haplogroup classification using a pangenomic reference graph
Source: PLoS Comput Biol. 2023 Jun 7;19(6):e1011148. doi: 10.1371/journal.pcbi.1011148 (PMC10281577; doi:10.1371/journal.pcbi.1011148)
Supplement: S1 File — Table A: Ground truth haplogroups of samples using in the downsampling and masking experiments. Haplogroups were determined by running HaploGrep2 classify with the --phylotree 17 flag. Sample JX154035 was excluded from downsampling experiments as described but is still shown in the posterior plots. Table B: Ground truth haplogroups in empirical paired-end FASTQ experiments. Samples were chosen such that each sample comes from a distinct sequencing center and so that the dataset reflects a high degree of geographic diversity. Africa is intentionally over-represented owing to the fact that African populations harbor the majority of the mitodiversity of humans. Table C: Background frequencies of nucleobases in the graph. Values were computed with the ODGI stats -S command. Note that these statistics are on the graph itself, not the embedded haplogroup, meaning that each base is counted once even if it is traversed by multiple embedded paths. Table D: Runtime and Peak Memory Usage on FASTA input. Results are averaged over three input samples (NCBI accessions MZ387838, MW057682, MN894713). Statistics were measured with the command /usr/bin/time -v. User time and wall clock time are reported to two significant digits, while peak memory usage is rounded to the nearest integer. In all cases HaploCart was run in quiet mode (-q) without posterior calculations (-np). Table E: Runtime and Peak Memory Usage on FASTA input. Results are averaged over three input samples (NCBI accessions MZ387838, MW057682, MN894713). Statistics were measured with the command /usr/bin/time -v. User time and wall clock time are reported to two significant digits, while peak memory usage is rounded to the nearest integer. In all cases HaploCart was run in quiet mode (-q) without posterior calculations (-np). Table F: Runtime and Peak Memory Usage on FASTA input. Results are averaged over three input samples (NCBI accessions MZ387838, MW057682, MN894713). Statistics were measured with the command /usr [file pcbi.1011148.s001.pdf]

Supplementary Material for `HaploCart`: Human mtDNA Haplogroup Classification Using a Pangenomic Reference Graph

Joshua Daniel Rubin[1]

Nicola Vogel[1]

Peter Wad Sackett[1]

Shyam Gopalakrishnan[2]

Gabriel Renaud[1]

[1]Department of Health Technology, Section for Bioinformatics, Technical University of Denmark, Kongens Lyngby, Denmark
[2] Center for Evolutionary Hologenomics, GLOBE Institute, Faculty of Health and Medical Sciences, Copenhagen, Denmark

May 18, 2023

Contents

1 Methods **1**
1.1 Mappability Scores from `GenMap` . . . 1
1.2 Incorporation of Artificial NuMT Reads . . . 2
1.3 PCR Duplicate Removal . . . 2
1.4 Test Data and Nucleotide Frequencies . . . 2

2 Results **4**
2.1 Empirical Data (Consensus FASTA) . . . 4
2.1.1 `HaploGrouper` Predictions on Empirical Consensus FASTA Sequences . . . 8
2.2 Simulated Data in FASTQ Format . . . 9
2.3 Simulated Paired-end FASTQ Posterior Plots . . . 10
2.4 Empirical Paired-end FASTQ Posterior Plots . . . 58
2.5 Runtime and Peak Memory Usage . . . 66
2.6 Empirical Data in FASTA Format . . . 68
2.7 Empirical Ancient Data in Consensus FASTA Format . . . 74
2.8 Empirical Ancient Data in BAM Format . . . 75
2.9 Empirical Ancient Data in BAM Format, Subsampled . . . 76

1 Methods

1.1 Mappability Scores from `GenMap`

To obtain site-specific mappability scores, chromosomes 1 to 22 plus the X chromosome from human genome build CHM13v1.1 were concatenated with the Y chromosome and the chrY_KI270740v1_random scaffold from hg38 and the rCRS to form a new reference genome. This reference was indexed using `GenMap` v1.3.0 with default parameters and subsequently mapped with the `map` command from `GenMap` with parameters `-K 30 -E 2 -t -w -bg -fl`[**19**]. The resultant per-base mappability scores are then used to calibrate the quantity $P(\neg M)$, i.e. the probability that a read is mismapped or originates from a NuMT, as described in the Inference section of the main text.

1.2 Incorporation of Artificial NuMT Reads

Our database of NuMT reads was taken from the UCSC Genome Browser, the data being "provided by Francesco Maria Calabrese, Domenico Simone and Marcella Attimonelli from the Department of Biochemistry and Molecular Biology "Ernesto Quagliariello (University of Bari, Italy)". These sequences were "[o]btained by running Blast2seq (program: `BlastN`) between each chromosome of of the Human Genome hg18 build and the human mitochondrial reference sequence (rCRS, AC: NC_012920), fixing the e-value threshold to 1e-03." Mapping coordinates were lifted over from hg18 to hg19 by using the Lift-Over part of the Galaxy software suite. Assembly of the HSPs was performed "with spreadsheet interpolation and manual inspection. BED format is used for the first three annotation tracks, while for the last one the SAM/BAM format is preferred"[**10**, **22**, **7**].

Each included NuMT read was selected uniformly from this compiled database of NuMT sequences of variable length using a custom script (`add_numt.sh` in the data repository - see the "Availability of Data and Materials" section of the main text).

1.3 PCR Duplicate Removal

`HaploCart` removes duplicate PCR reads from a GAM file before performing inference. This step is necessary because the inference algorithm assumes independence among reads, and if this assumption does not hold we may over-penalize errors in the read sequences.

We have implemented a subcommand within the `vgan` package for duplicate removal called `vgan duprm`. `HaploCart` calls this subcommand internally but it is also available for use as a standalone program. The subcommand requires that the GAM file has already been sorted with respect to coordinates using `vg gamsort`. Here we describe the procedure used. First, a few definitions from `vg` parlance are required.

Each node in a `vg` graph is identified by a unique node identifier called a *node ID*. Each node is associated with a unique node sequence which can be read in one of two *orientations* (forward or reverse). If the orientation is reverse the sequence must be read as a reverse complement. The index of a given base in a node sequence from the standpoint of a given orientation is termed the *offset* of that base. When `vg giraffe` maps a read to the graph, it is possible (and very likely) for the read to be mapped to multiple nodes. Each alignment of a read subsequence to a node sequence in a given orientation is termed a *mapping*, and the totality of all mappings of a read to connected nodes is called a *path*[**28**].

Our PCR duplicate removal procedure runs as follows. First we record the starting coordinates (node id and offset) of the first mapping to a node of the first read in the sorted GAM file. Call this read the *candidate*. Then for each subsequent read (call it the *putative duplicate*) we check whether the starting coordinates between the candidate and putative duplicate are the same. For paired-end reads we also require that the ending coordinates (i.e. the node ID and offset of the final mapping) are the same between the candidate and putative duplicate.

After this is done, the second read in the sorted GAM file becomes the new candidate, the third read becomes the putative duplicate, and we repeat the loop described above. This procedure continues until every pair of reads in the GAM file has been checked. Reads which have been marked as duplicates are then removed.

1.4 Test Data and Nucleotide Frequencies

The table of the FASTA IDs is found in Table S1, information about the 1000 Genomes samples used is found in Table S2 and nucleotide background frequency is found in Table S3.

`HaploGrouper` Results on Empirical Consensus FASTA Sequences

`HaploGrouper` takes as input VCF files. To obtain `HaploGrouper` predictions on consensus FASTA sequences we first perform a pairwise alignment with the rCRS using `minimap2`[**12**]. As with the paired-end FASTQ dataset we then call variants with `bcftools` under a haploid model (options: –ploidy 1 -c -v -Oz).

Software Versions

For all experiments we used `HaploCheck` version 1.3.2 and `HaploGrep2` version 2.4.0. All experiments were performed using `VG` version `Obolo`. All `samtools` commands used version 1.13. Our Snakemake version was 5.10.0. Our `ART` version was 2.5.8. Our `ODGI` version was v0.6.3-52-g0d7f950 ("Pulizia"). We downloaded `HaploGrouper` from the Gitlab repository `https://gitlab.com/bio_anth_decode/haploGrouper` with commit SHA e4c0a0e0. Our `bcftools` version was 1.10.2 using `htslib` version 1.10.2-3. Our `minimap2` version was 2.24-r1122. Our `ANGSD` version was 0.935 (htslib: 1.13).

Supplementary Table A: Ground truth haplogroups of samples using in the downsampling and masking experiments. Haplogroups were determined by running `HaploGrep2 classify` with the `--phylotree 17` flag. Sample JX154035 was excluded from downsampling experiments as described but is still shown in the posterior plots.

Haplogroup	NCBI Accession
HV4b	KP340180
L0a1a1	MK295855
L1c4b	MN894773
B2b3a	MW057682
H2a2a1	JX154035 [1]
J2a1a1a1	MZ190830
L2a1a3c	KR135866
T2e1a1b1	JN828512
L2a1j	KR135846
L1c2b	MN894780 [2]
Q1	MN849793
C1b	MN894713
Z1a	MG660559
D1	KP172430
A2	MZ387838
Y1b	GU123044
P	MN849673
L3b1	KT819256
U2e1b1	KT698031
F1a1	MH553920
I2b	MN516596
S1a	DQ404440
V3	MN516629
X3a	JQ245804
E1a1b	EF061151

[1] the sample was removed due to being the same haplogroup as the rCRS, see main methods.
[2] the sample was removed due to unstable mutations, see main methods.

Supplementary Table B: Ground truth haplogroups in empirical paired-end FASTQ experiments. Samples were chosen such that each sample comes from a distinct sequencing center and so that the dataset reflects a high degree of geographic diversity. Africa is intentionally over-represented owing to the fact that African populations harbor the majority of the mitodiversity of humans.

Haplogroup	NCBI Accession	Population
D6a1a	HG00473	Southern Han Chinese (CHS)
K1a4a1h	HG01051	Puerto Rican in Puerto Rico (PUR)
L3e4a	HG02666	Gambian in Western Division, The Gambia - Mandinka (GWD)
L1b1a3	HG03112	Esan in Nigeria (ESN)
L0a1a3	NA18510	Yoruba in Ibadan, Nigeria (YRI)
L3b1a1a	NA19036	Luhya in Webuye, Kenya (LWK)
D1h1	NA19661	Mexican Ancestry in Los Angeles, California (MXL)
J2a1a1e	NA20518	Toscani in Italy (TSI)

Supplementary Table C: Background frequencies of nucleobases in the graph. Values were computed with the `ODGI stats -S` command. Note that these statistics are on the graph itself, not the embedded haplogroup, meaning that each base is counted once even if it is traversed by multiple embedded paths.

Base	Background frequency
A	0.27532
C	0.30044
T	0.25780
G	0.16644

2 Results

We present additional results for empirical data in FASTA format on page 9 and for simulated paired-end NGS read data in FASTQ format on page 9.

2.1 Empirical Data (Consensus FASTA)

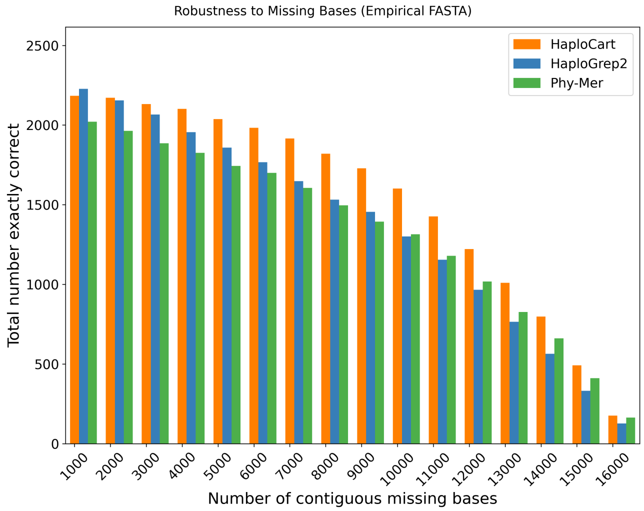

Supplementary Figure A: **Total Number of Exactly Correct Predictions as a Function of the Number of Contiguous Masked Bases on Consensus FASTA Input.** Counts are provided for `HaploCart`, `Phy-Mer`, and `HaploGrep2`. `HaploCart` outperforms the other two programs from 2Kb up to 16Kb.

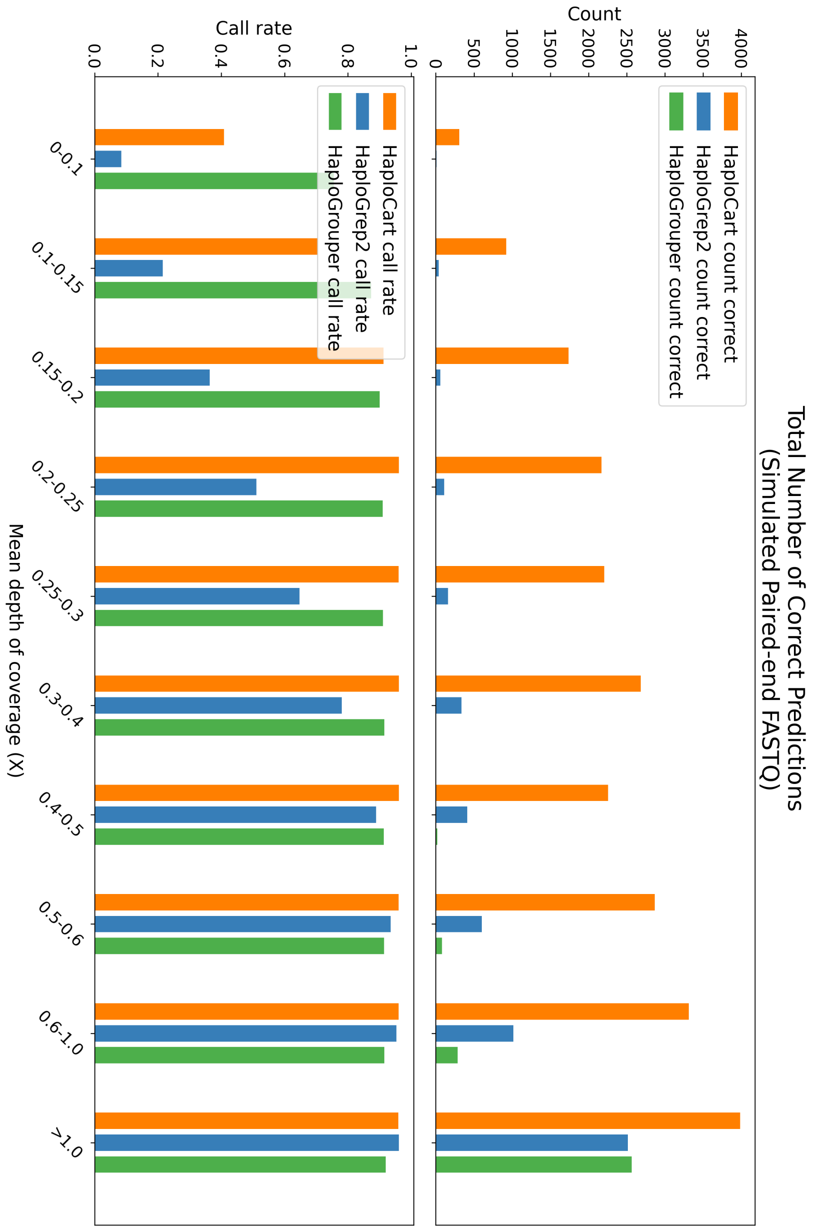

Supplementary Figure B: **Total number of predictions and call rates on simulated paired-end FASTQ data.** $[TOP]$ Total number of predictions on the simulated replicates which exactly match the underlying haplogroup, as determined by running `HaploCheck` at full coverage. $[BOTTOM]$ Call rates (i.e. proportion of samples for which a haplogroup assignment is provided by the program). For each window, `HaploCart` outperforms `HaploGrep2` and `HaploGrouper` by providing more reliable haplogroup assignments at a higher call rate.

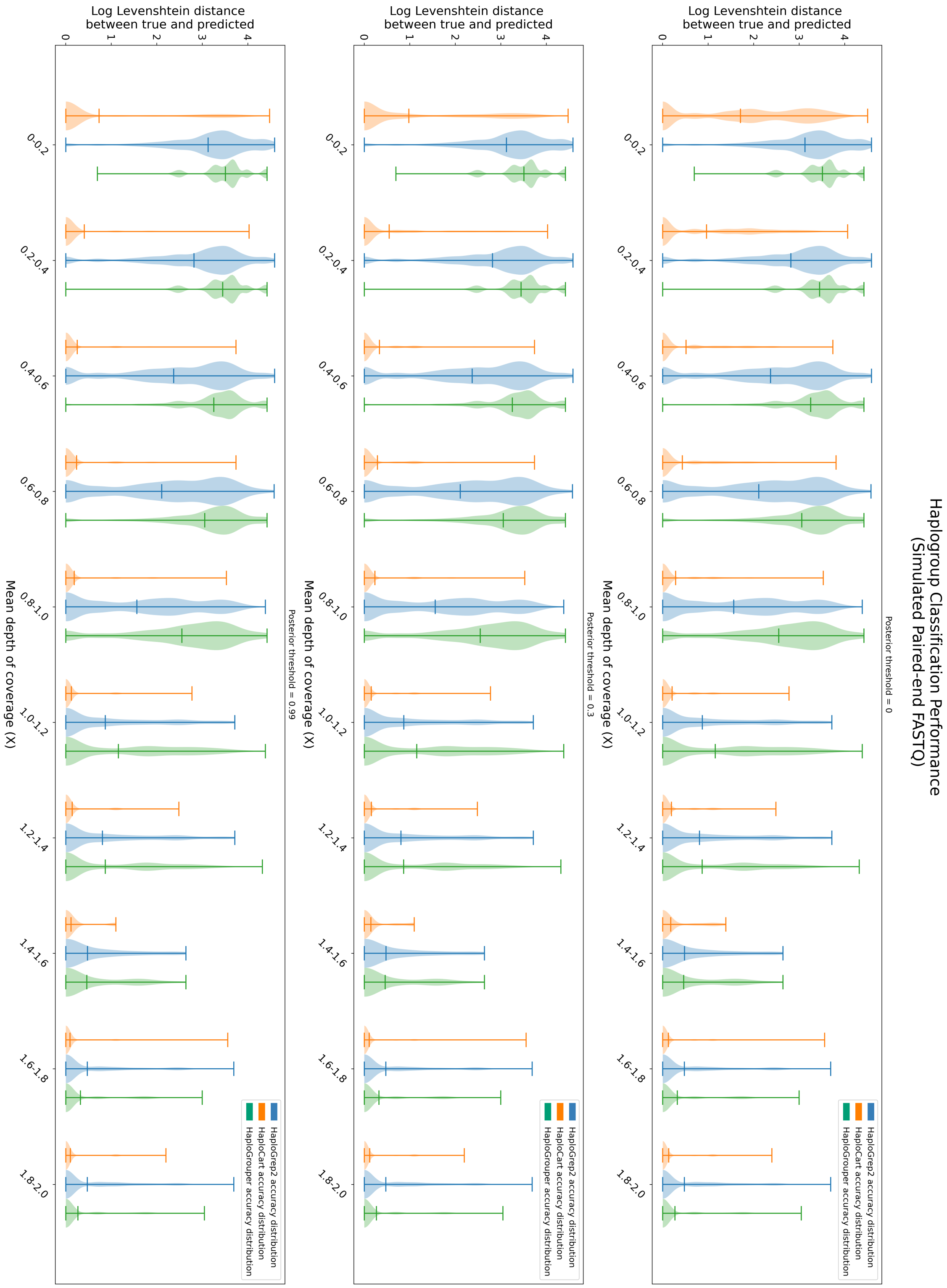

Supplementary Figure C: **Simulated Paired-end Downsampled FASTQ Samples at Varying Posterior Thresholds.** Distribution of log edit (Levenshtein) distances on the simulated paired-end FASTQ dataset at three different lower thresholds (0, 0.3, 0.99) on the `HaploCart` posterior probability of the haplogroup assignment. No threshold is applied to `HaploGrep2` or `HaploGrouper`. We observe a clear improvement in the edit distances of the most anomalous predictions as the threshold increases, which demonstrates the utility of `HaploCart` posterior probabilities for use in quality control.

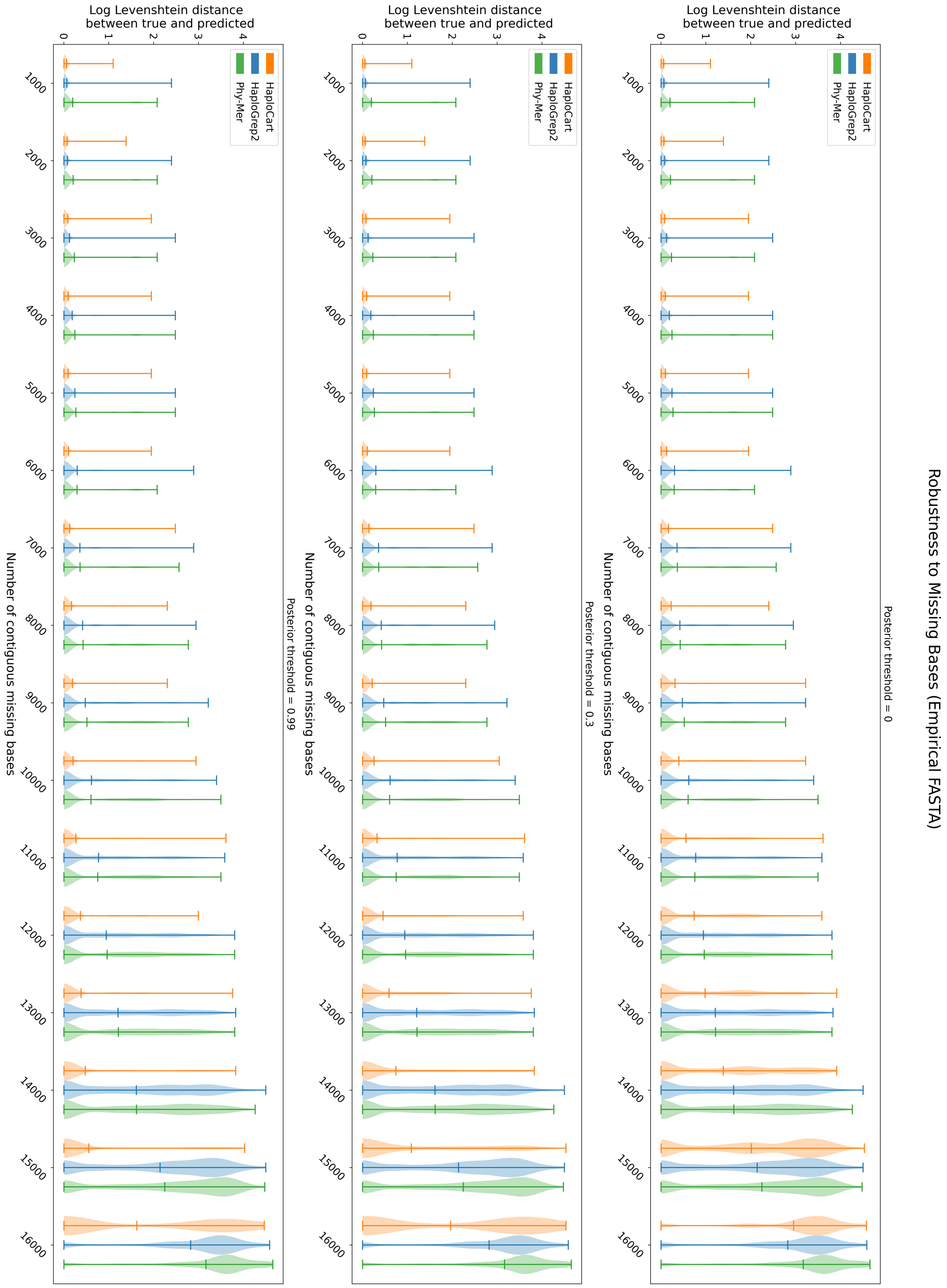

Supplementary Figure D: **Masking Experiment on Consensus FASTA Input at Varying Posterior Thresholds.** Distribution of edit (Levenshtein) distances on the masked consensus FASTA dataset at three different lower thresholds (0, 0.3, 0.99) on the `HaploCart` posterior probability of the haplogroup assignment. No threshold is applied to `HaploGrep2` or `Phy-Mer`. We observe a clear improvement in the edit distance distribution for `HaploCart` as the threshold increases, demonstrating the utility of `HaploCart` posterior probabilities for use in quality control.

2.1.1 HaploGrouper Predictions on Empirical Consensus FASTA Sequences

The vast majority of predictions concord with both HaploCart and HaploGrep2. For some samples, such as AY950293 and AY950293, HaploGrouper (prediction: M31a1) does not concord with a prediction shared by the other two programs (prediction: M31a1a). In another case (sample AF382002) we observe all three programs providing different predictions (H1a, H100, and HV_A73G), indicating that the underlying sample must be far from any sequence in the tree. We also see a case (sample AF346978) where HaploGrouper (prediction: HV0_T195C) agrees with HaploGrep2 (prediction: HV0+195) but not HaploCart (prediction: HV0d). Finally we see one case (sample AF381997) where HaploGrouper agrees with HaploCart on haplogroup R, but not with HaploGrep2 which calls haplogroup HV+73.

Since these data are empirical and may well constitute haplogroups outside the known tree, we do not have ground truth labels. Nonetheless, the fact that HaploGrouper does not seem to preferentially agree with either tool suggests that our program performs at least as well as HaploGrep2 at calling haplogroups on consensus sequences.

2.2 Simulated Data in FASTQ Format

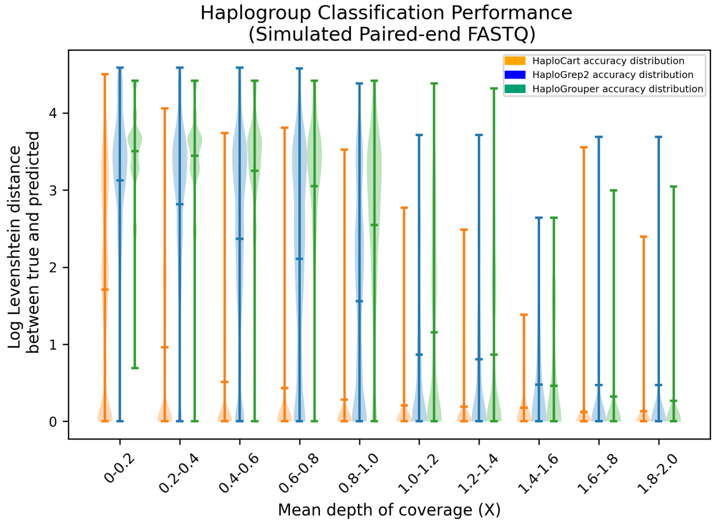

Supplementary Figure E: **Distribution of Log Edit Distances between Ground Truth and Predicted Haplogroups on Simulated FASTQ Data** Distribution of edit (Levenshtein) distances between assigned and underlying haplogroup of replicates from the simulated dataset. Central line represent the arithmetic mean of the distribution. For each window, `HaploCart` outperforms `HaploGrep2` and `HaploGrouper` at all coverage windows as evidenced by the mean of the distributions. Note that unlike `HaploGrep2` and `HaploGrouper`, `HaploCart` makes a prediction if even a single read maps to the graph.

2.3 Simulated Paired-end FASTQ Posterior Plots

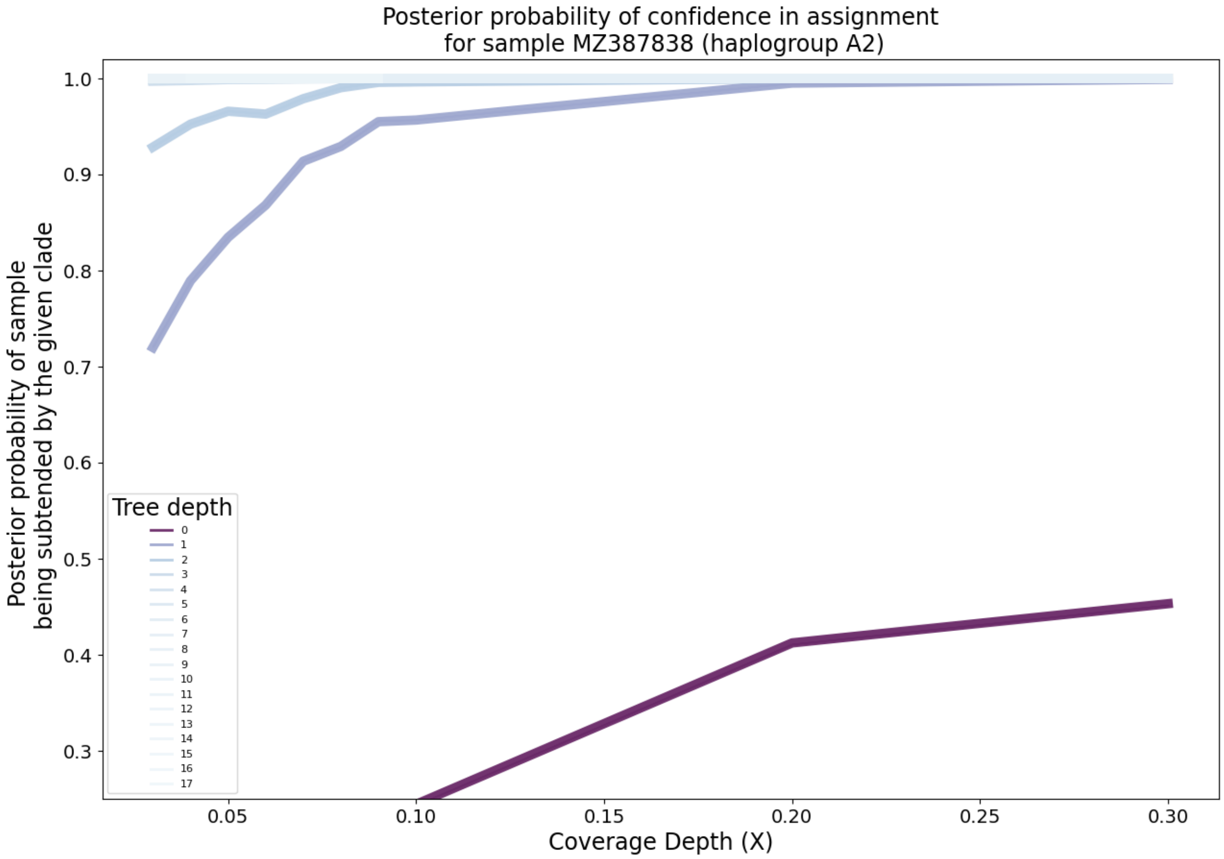

Supplementary Figure F: **Clade-level posterior probabilities of haplogroup assignment on simulated paired-end FASTQ data.** Each lineplot represents the mean over replicates at a fixed depth on the mitochondrial tree. The darker the line, the more basal the haplogroups.

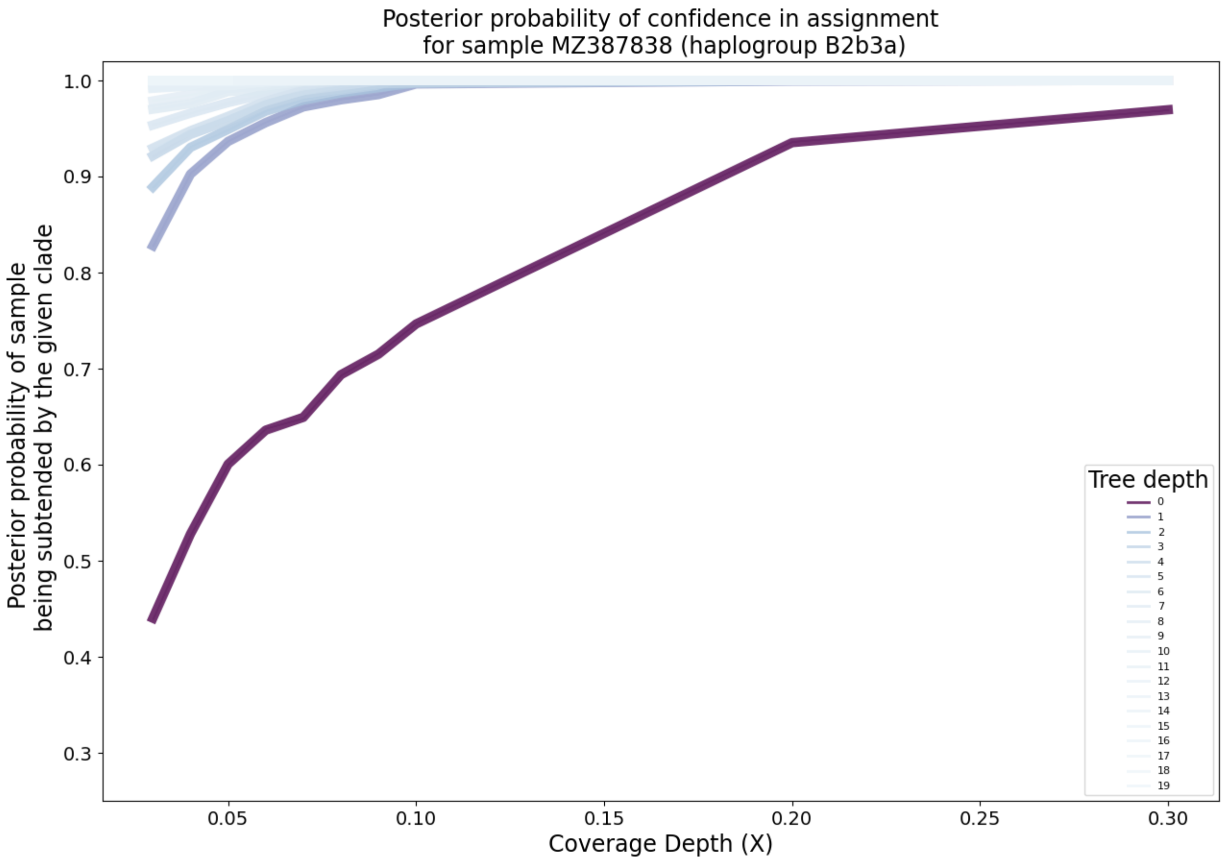

Supplementary Figure G: **Clade-level posterior probabilities of haplogroup assignment on simulated paired-end FASTQ data.** Each lineplot represents the mean over replicates at a fixed depth on the mitochondrial tree. The darker the line, the more basal the haplogroups.

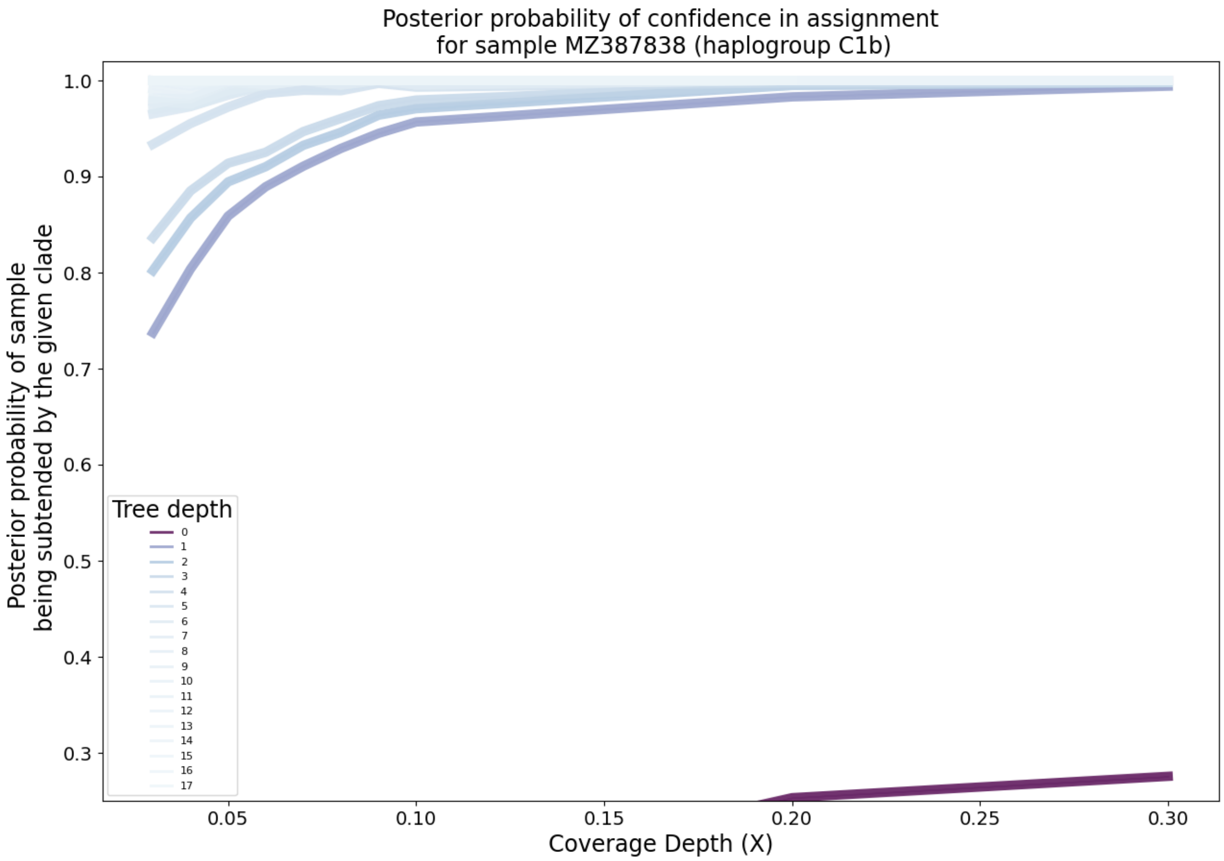

Supplementary Figure H: **Clade-level posterior probabilities of haplogroup assignment on simulated paired-end FASTQ data.** Each lineplot represents the mean over replicates at a fixed depth on the mitochondrial tree. The darker the line, the more basal the haplogroups.

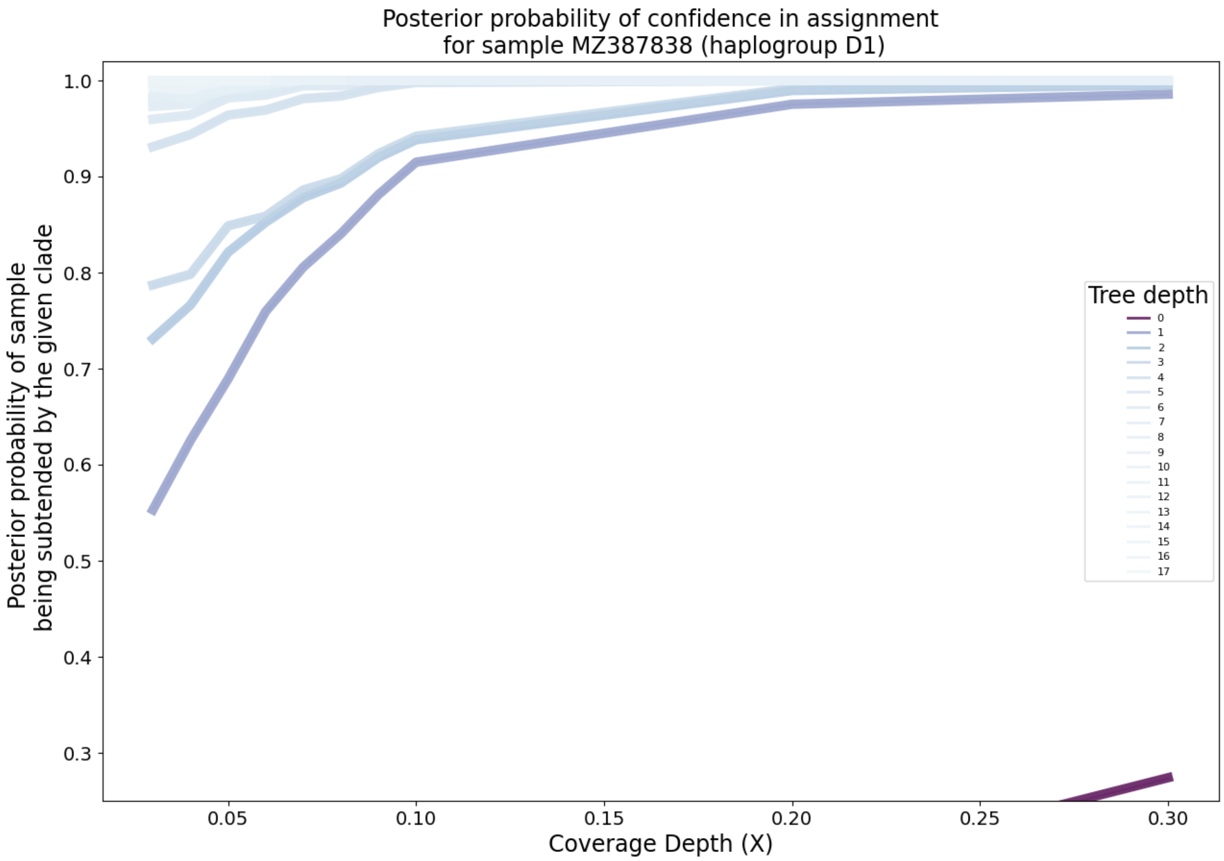

Supplementary Figure I: **Clade-level posterior probabilities of haplogroup assignment on simulated paired-end FASTQ data.** Each lineplot represents the mean over replicates at a fixed depth on the mitochondrial tree. The darker the line, the more basal the haplogroups.

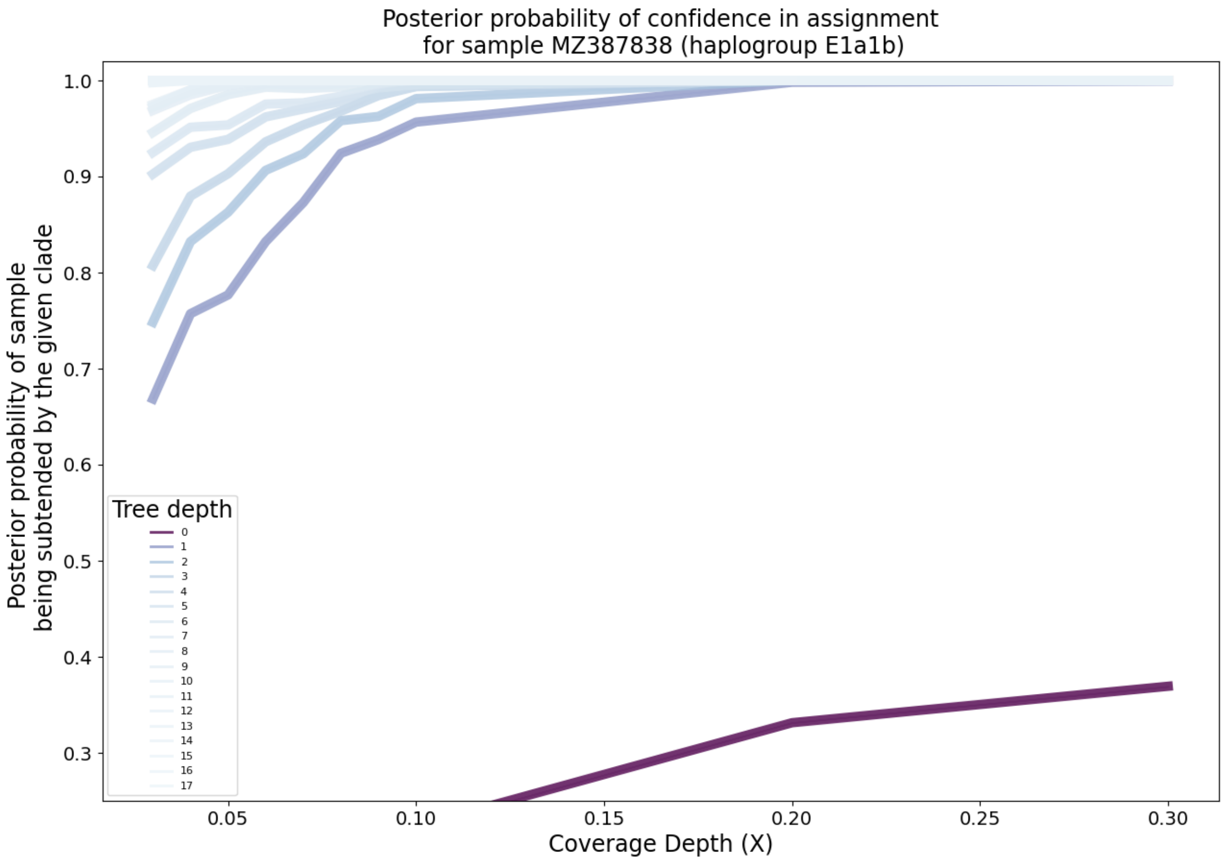

Supplementary Figure J: **Clade-level posterior probabilities of haplogroup assignment on simulated paired-end FASTQ data.** Each lineplot represents the mean over replicates at a fixed depth on the mitochondrial tree. The darker the line, the more basal the haplogroups.

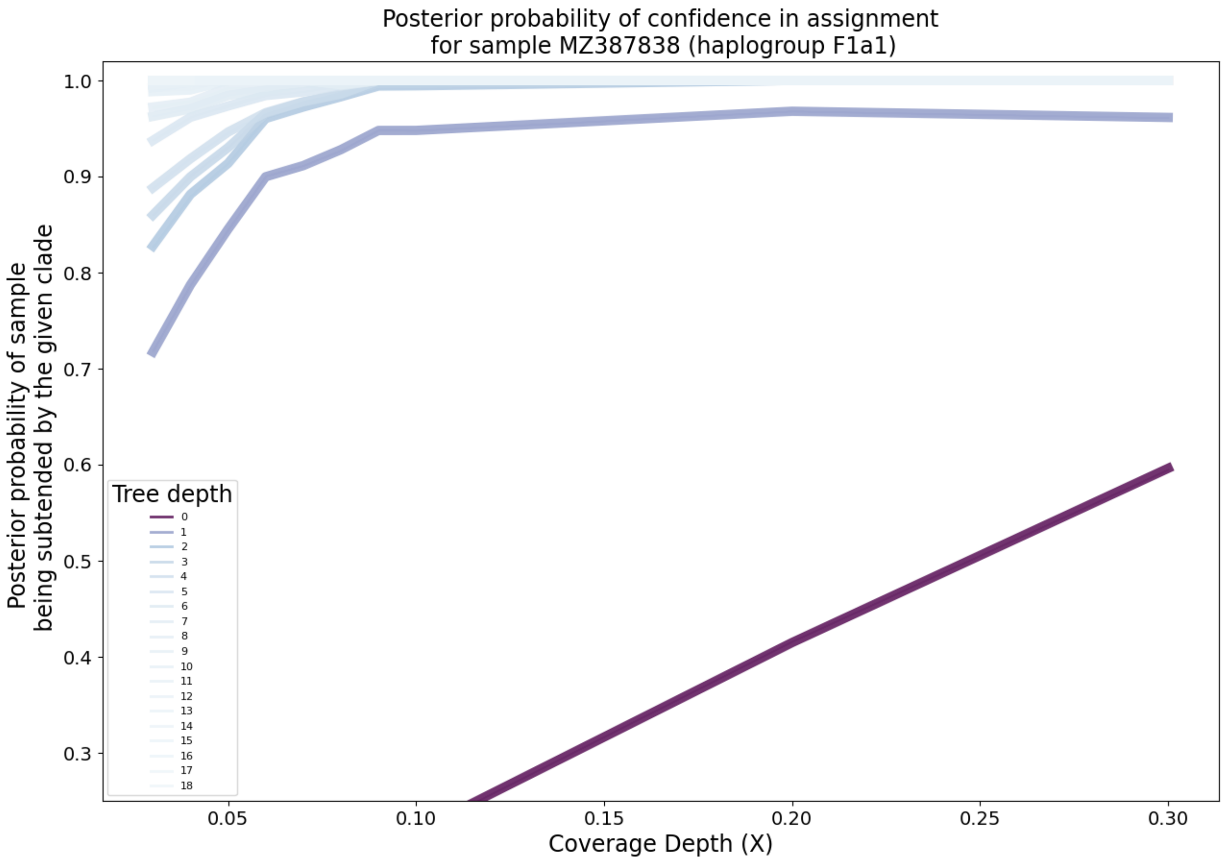

Supplementary Figure K: **Clade-level posterior probabilities of haplogroup assignment on simulated paired-end FASTQ data.** Each lineplot represents the mean over replicates at a fixed depth on the mitochondrial tree. The darker the line, the more basal the haplogroups.

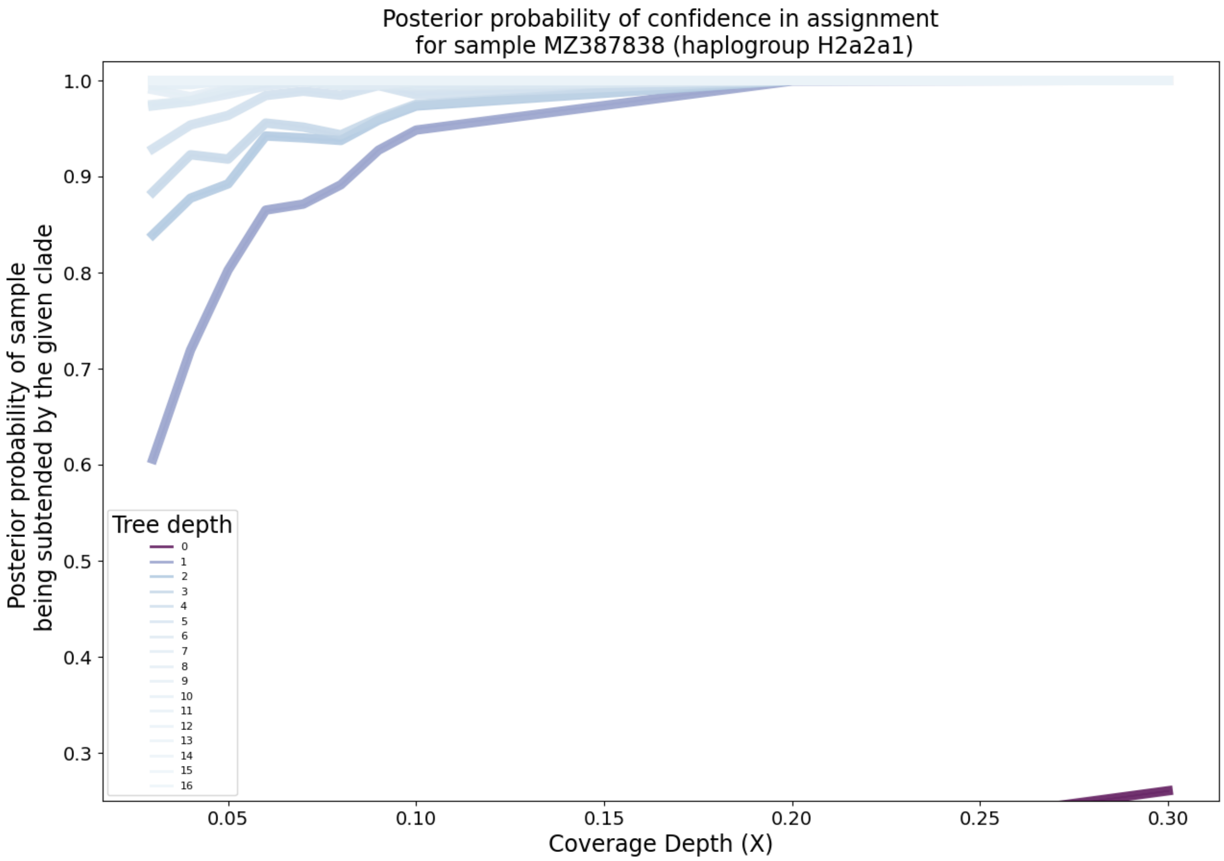

Supplementary Figure L: **Clade-level posterior probabilities of haplogroup assignment on simulated paired-end FASTQ data.** Each lineplot represents the mean over replicates at a fixed depth on the mitochondrial tree. The darker the line, the more basal the haplogroups.

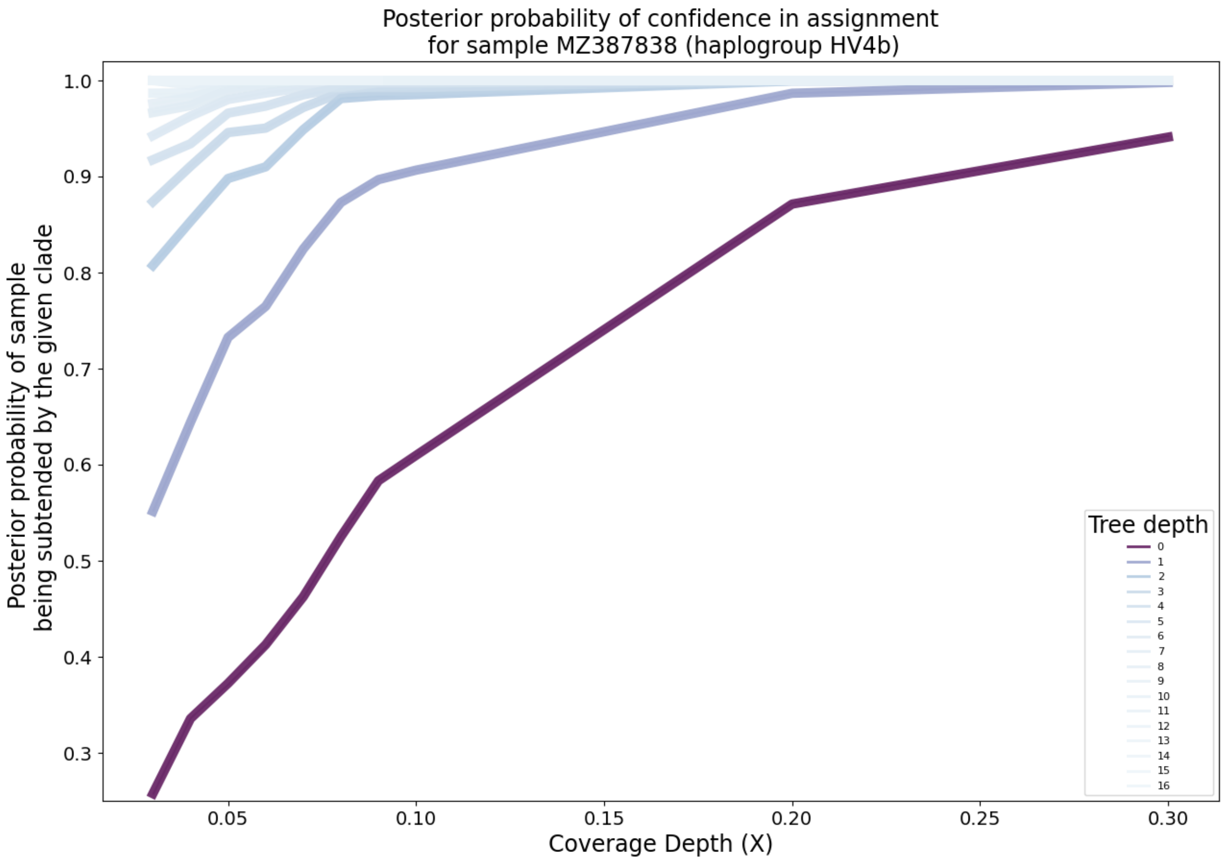

Supplementary Figure M: **Clade-level posterior probabilities of haplogroup assignment on simulated paired-end FASTQ data.** Each lineplot represents the mean over replicates at a fixed depth on the mitochondrial tree. The darker the line, the more basal the haplogroups.

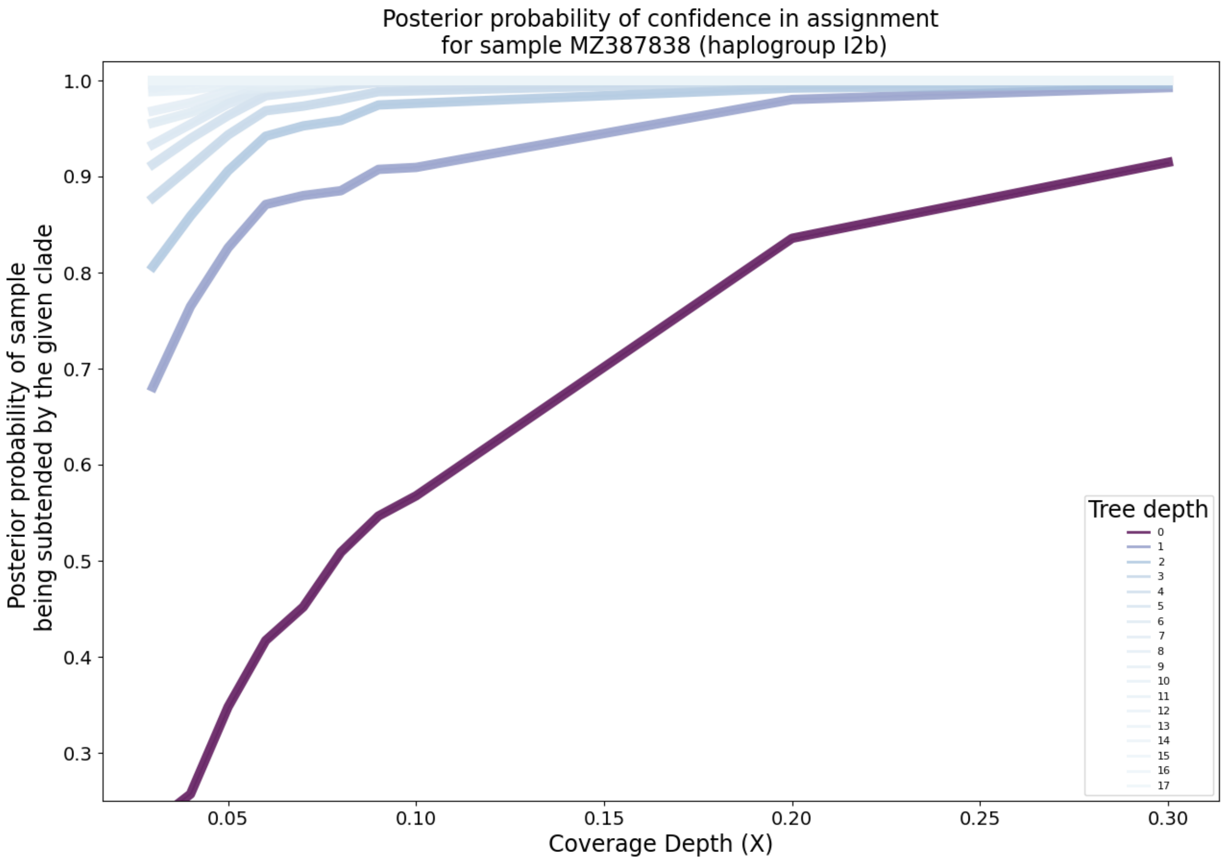

Supplementary Figure N: **Clade-level posterior probabilities of haplogroup assignment on simulated paired-end FASTQ data.** Each lineplot represents the mean over replicates at a fixed depth on the mitochondrial tree. The darker the line, the more basal the haplogroups.

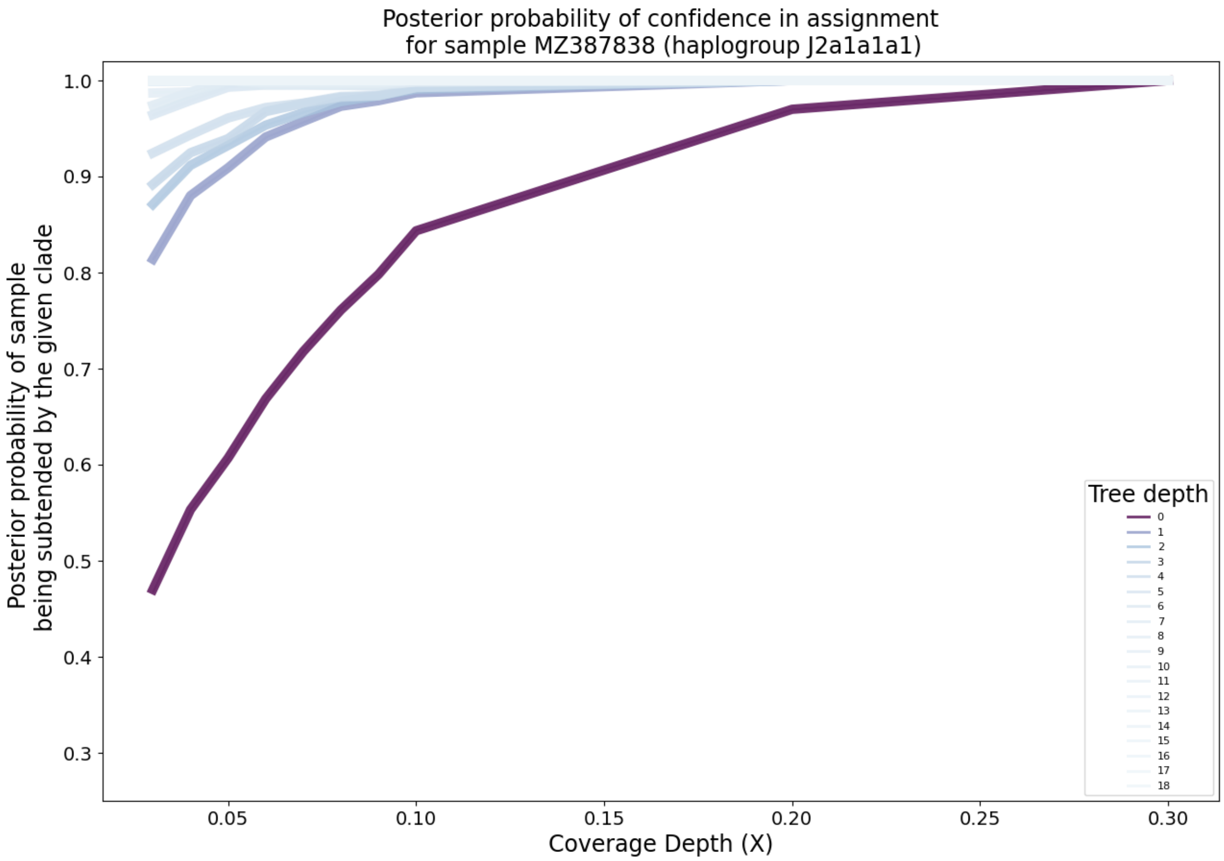

Supplementary Figure O: **Clade-level posterior probabilities of haplogroup assignment on simulated paired-end FASTQ data.** Each lineplot represents the mean over replicates at a fixed depth on the mitochondrial tree. The darker the line, the more basal the haplogroups.

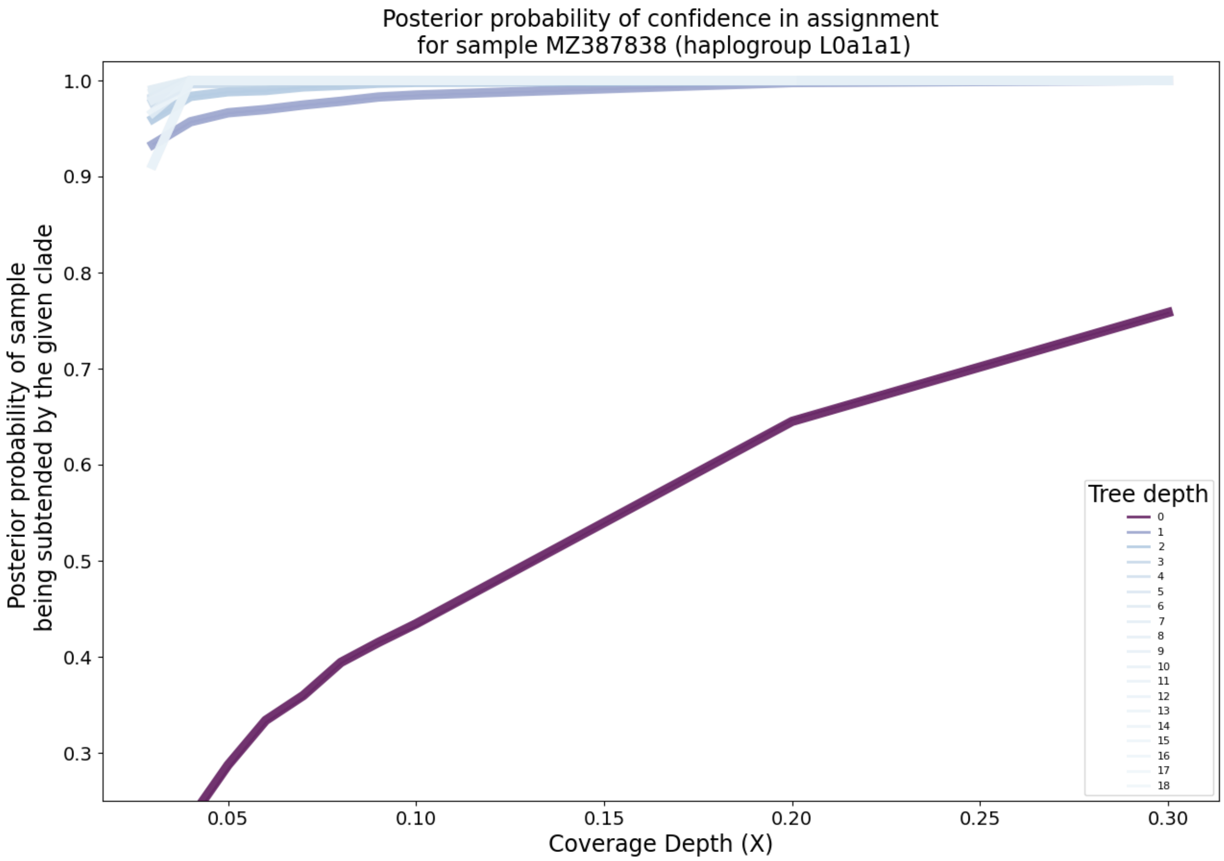

Supplementary Figure P: **Clade-level posterior probabilities of haplogroup assignment on simulated paired-end FASTQ data.** Each lineplot represents the mean over replicates at a fixed depth on the mitochondrial tree. The darker the line, the more basal the haplogroups.

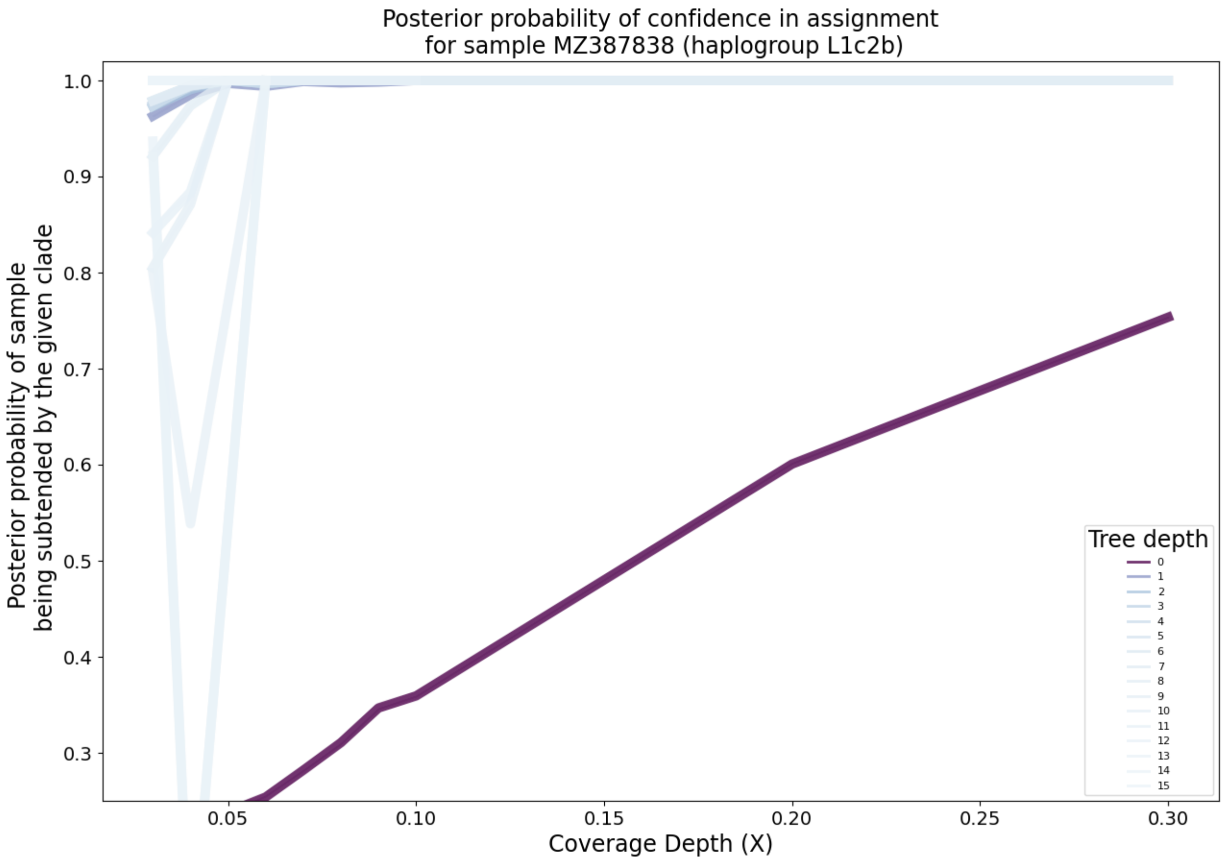

Supplementary Figure Q: **Clade-level posterior probabilities of haplogroup assignment on simulated paired-end FASTQ data.** Each lineplot represents the mean over replicates at a fixed depth on the mitochondrial tree. The darker the line, the more basal the haplogroups.

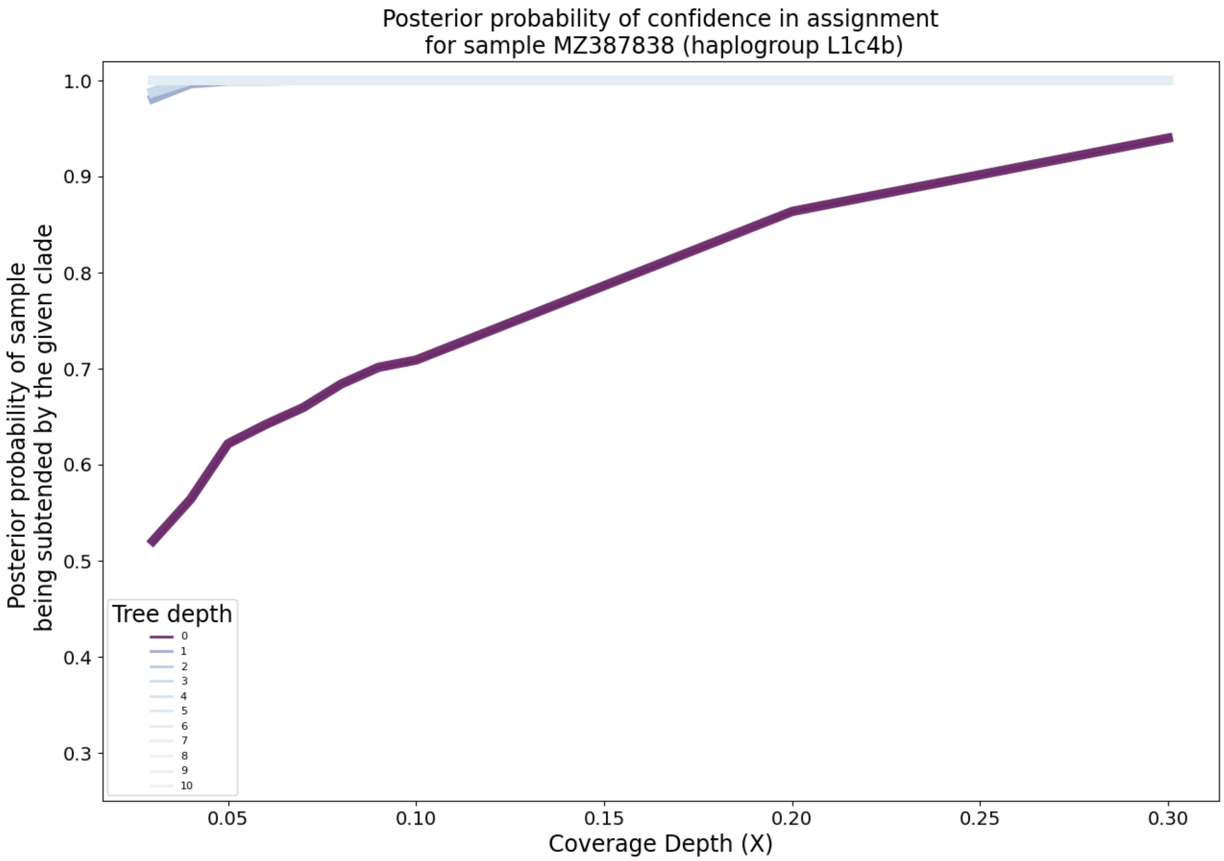

Supplementary Figure R: **Clade-level posterior probabilities of haplogroup assignment on simulated paired-end FASTQ data.** Each lineplot represents the mean over replicates at a fixed depth on the mitochondrial tree. The darker the line, the more basal the haplogroups.

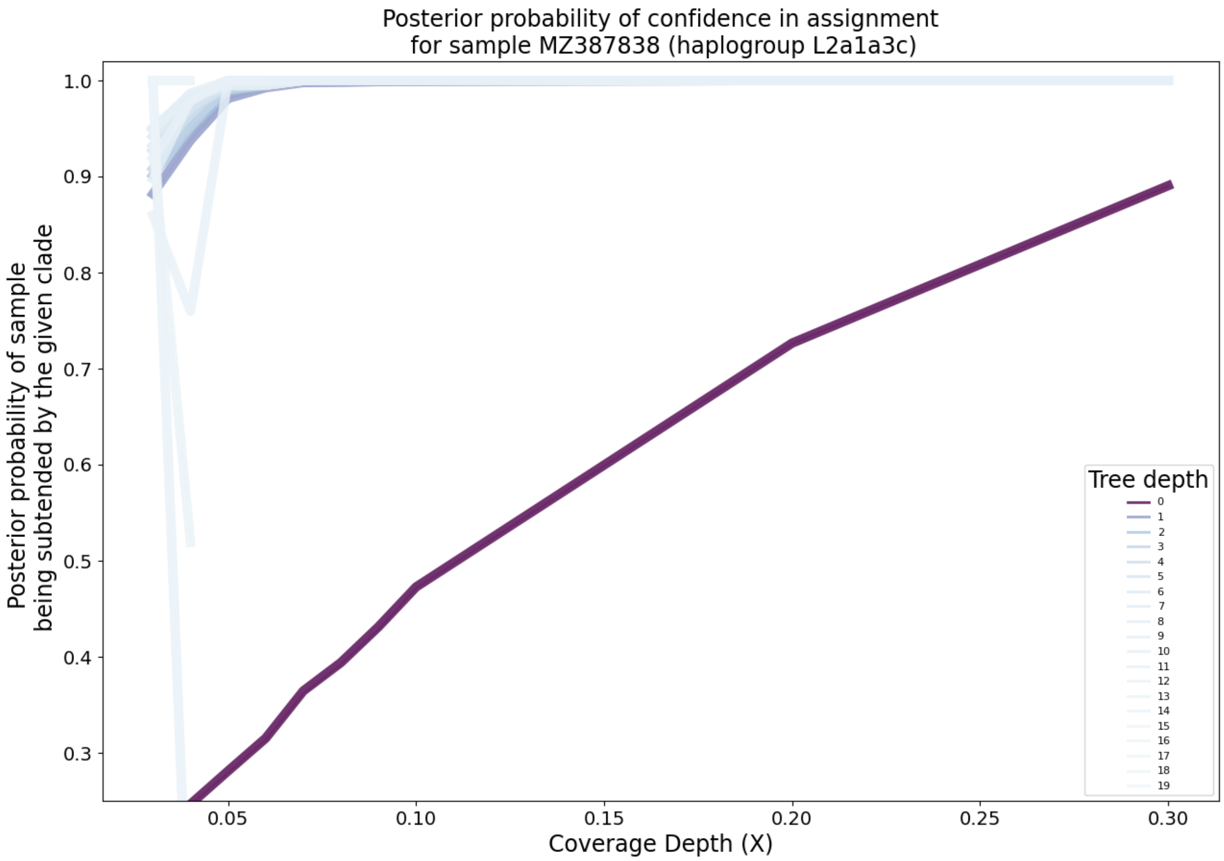

Supplementary Figure S: **Clade-level posterior probabilities of haplogroup assignment on simulated paired-end FASTQ data.** Each lineplot represents the mean over replicates at a fixed depth on the mitochondrial tree. The darker the line, the more basal the haplogroups.

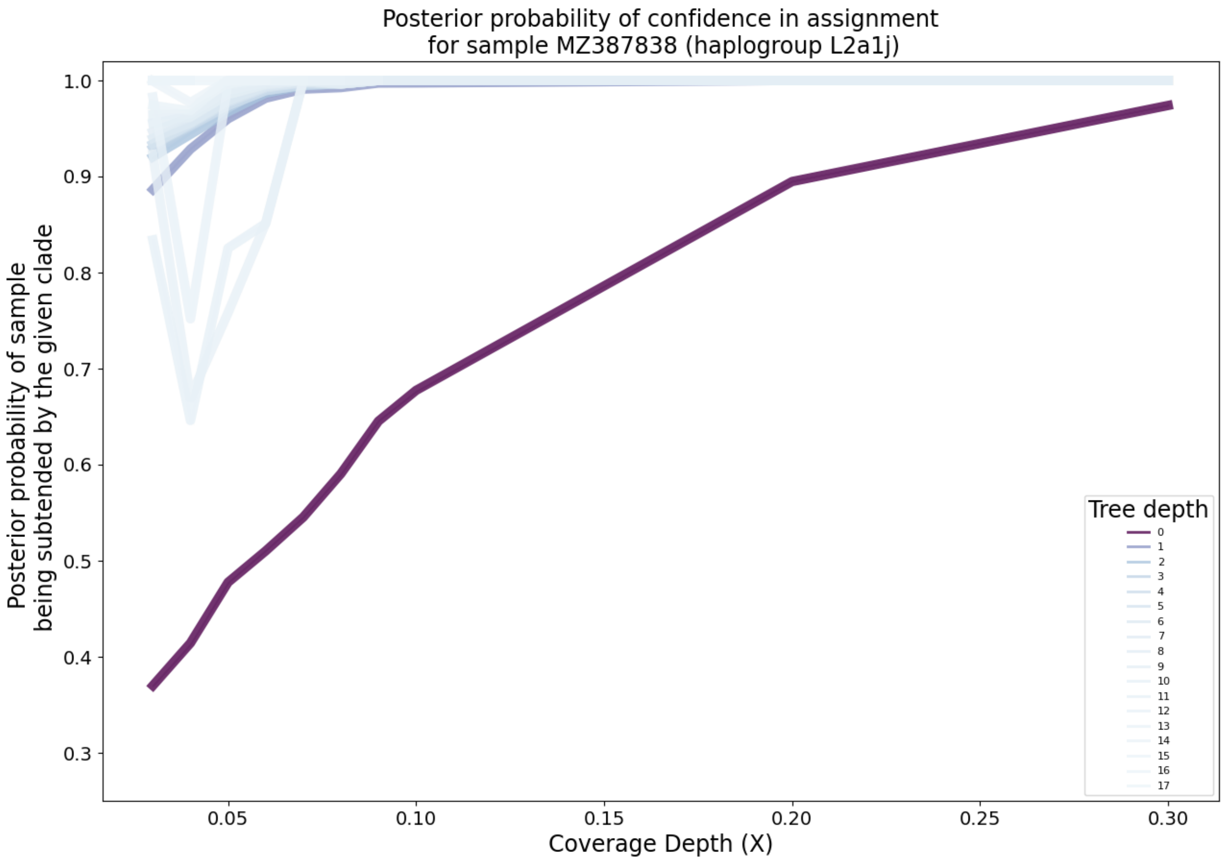

Supplementary Figure T: **Clade-level posterior probabilities of haplogroup assignment on simulated paired-end FASTQ data.** Each lineplot represents the mean over replicates at a fixed depth on the mitochondrial tree. The darker the line, the more basal the haplogroups.

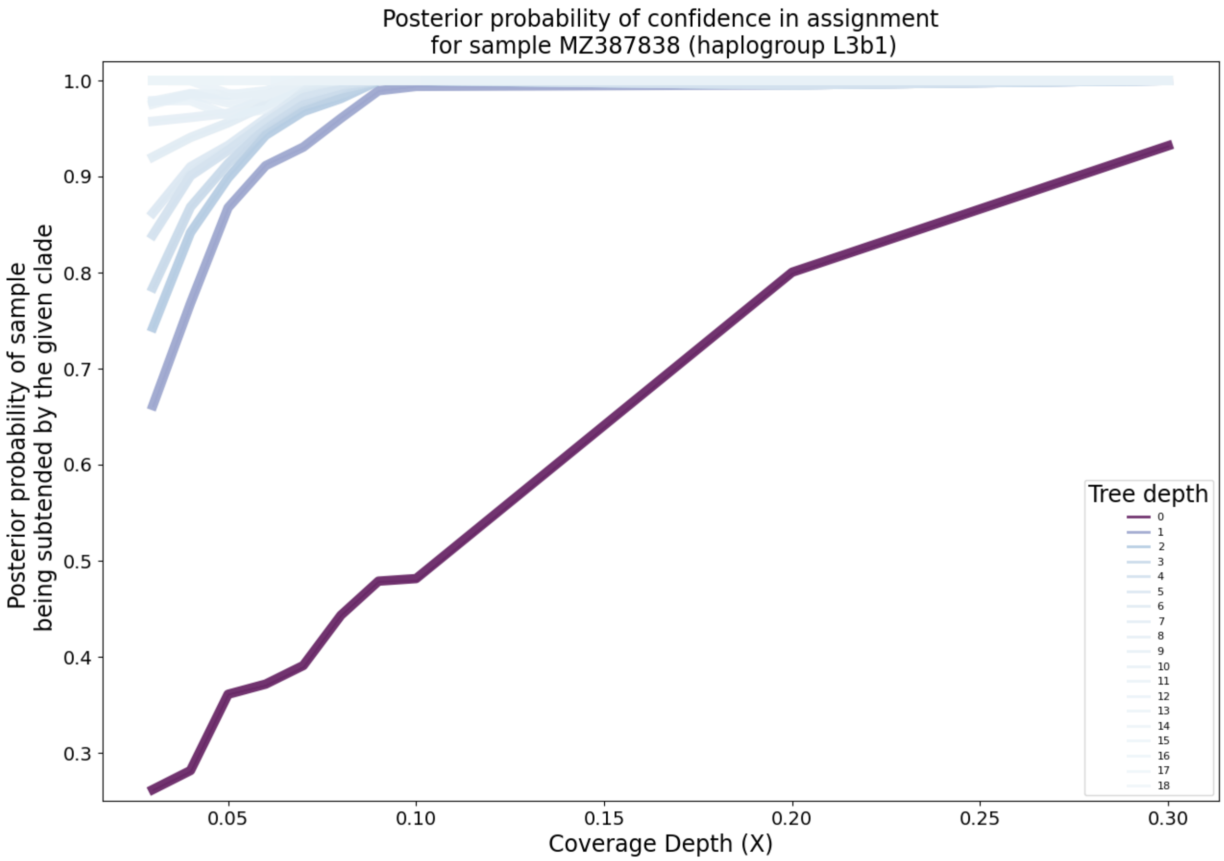

Supplementary Figure U: **Clade-level posterior probabilities of haplogroup assignment on simulated paired-end FASTQ data.** Each lineplot represents the mean over replicates at a fixed depth on the mitochondrial tree. The darker the line, the more basal the haplogroups.

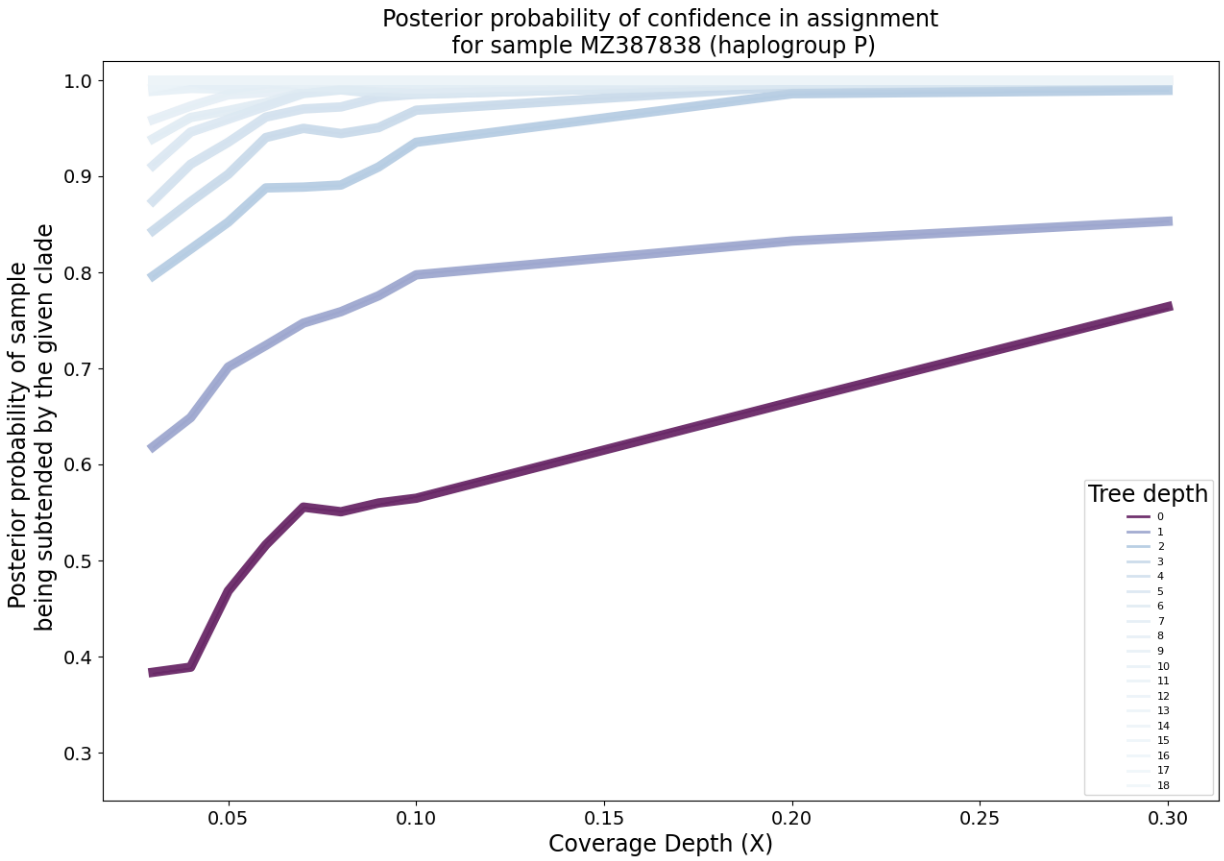

Supplementary Figure V: **Clade-level posterior probabilities of haplogroup assignment on simulated paired-end FASTQ data.** Each lineplot represents the mean over replicates at a fixed depth on the mitochondrial tree. The darker the line, the more basal the haplogroups.

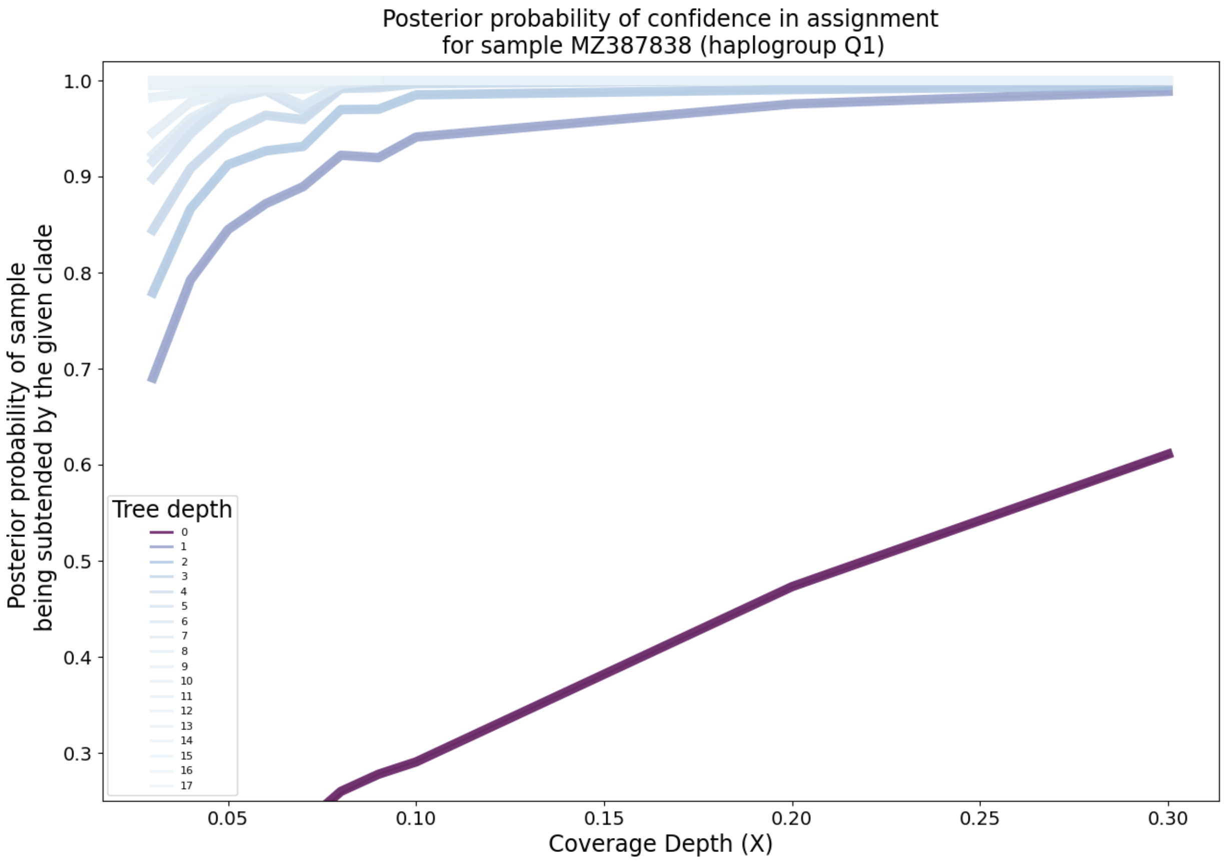

Supplementary Figure W: **Clade-level posterior probabilities of haplogroup assignment on simulated paired-end FASTQ data.** Each lineplot represents the mean over replicates at a fixed depth on the mitochondrial tree. The darker the line, the more basal the haplogroups.

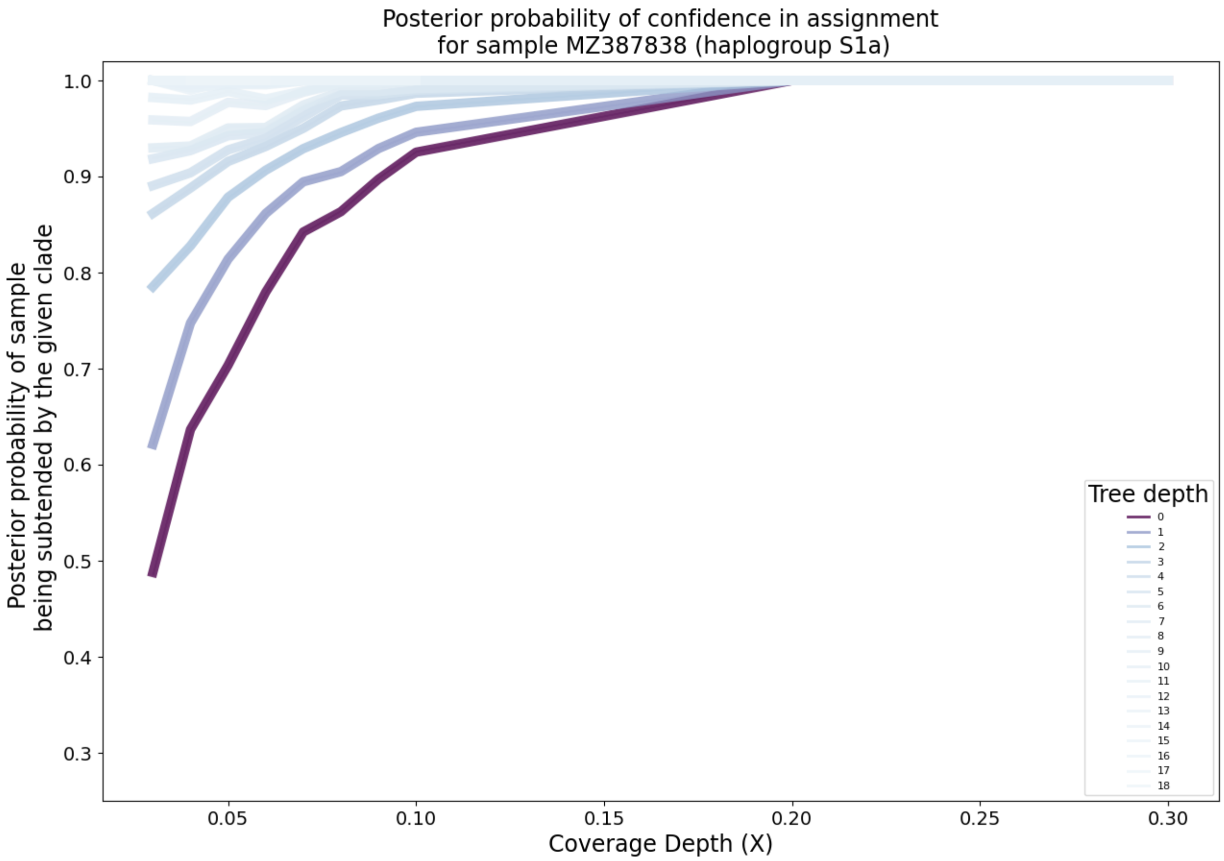

Supplementary Figure X: **Clade-level posterior probabilities of haplogroup assignment on simulated paired-end FASTQ data.** Each lineplot represents the mean over replicates at a fixed depth on the mitochondrial tree. The darker the line, the more basal the haplogroups.

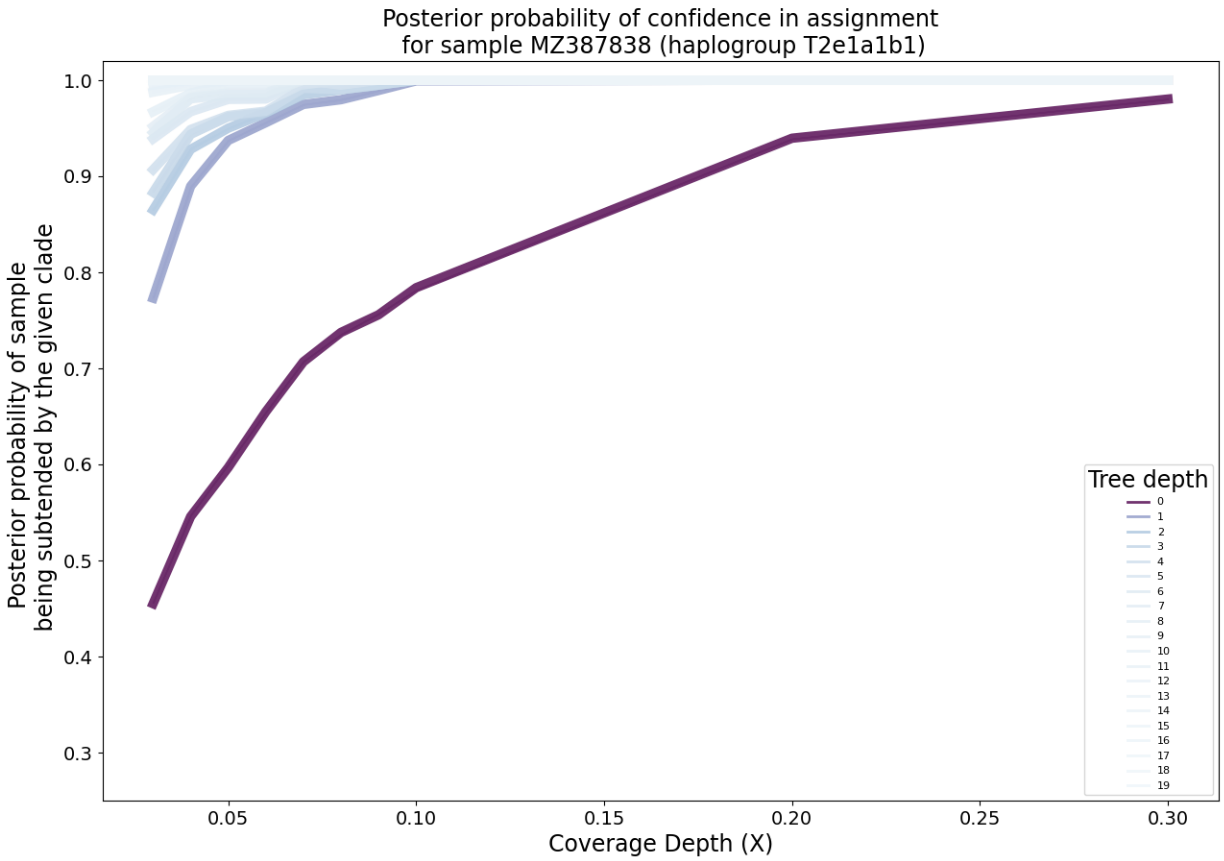

Supplementary Figure Y: **Clade-level posterior probabilities of haplogroup assignment on simulated paired-end FASTQ data.** Each lineplot represents the mean over replicates at a fixed depth on the mitochondrial tree. The darker the line, the more basal the haplogroups.

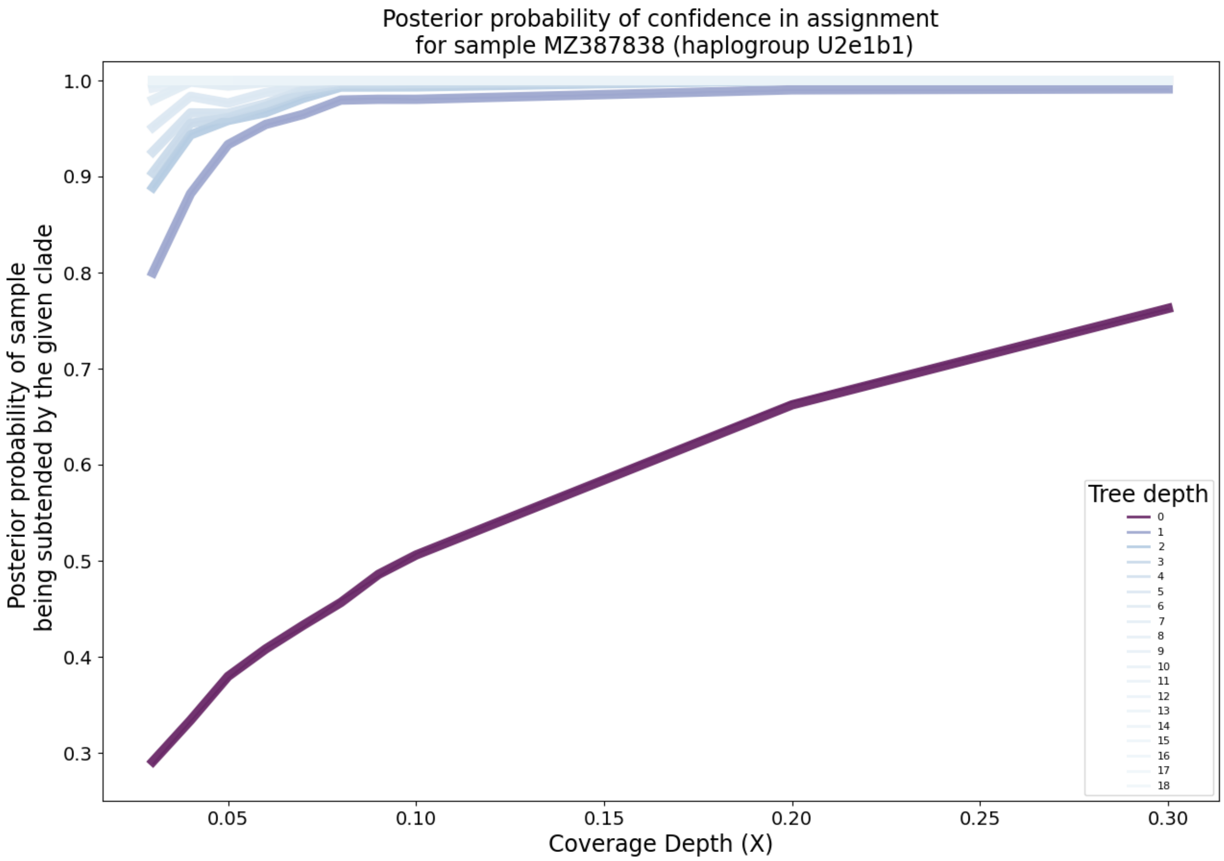

Supplementary Figure Z: **Clade-level posterior probabilities of haplogroup assignment on simulated paired-end FASTQ data.** Each lineplot represents the mean over replicates at a fixed depth on the mitochondrial tree. The darker the line, the more basal the haplogroups.

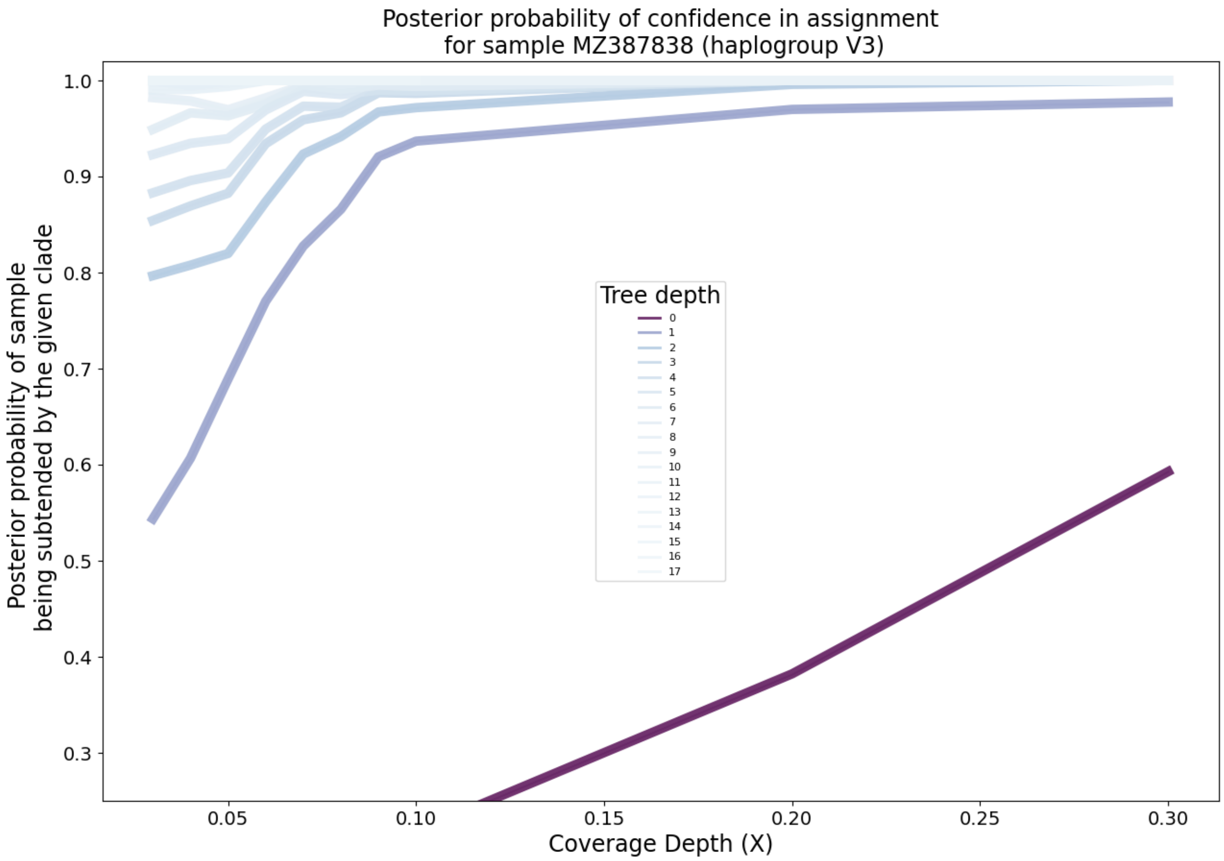

Supplementary Figure AA: **Clade-level posterior probabilities of haplogroup assignment on simulated paired-end FASTQ data.** Each lineplot represents the mean over replicates at a fixed depth on the mitochondrial tree. The darker the line, the more basal the haplogroups.

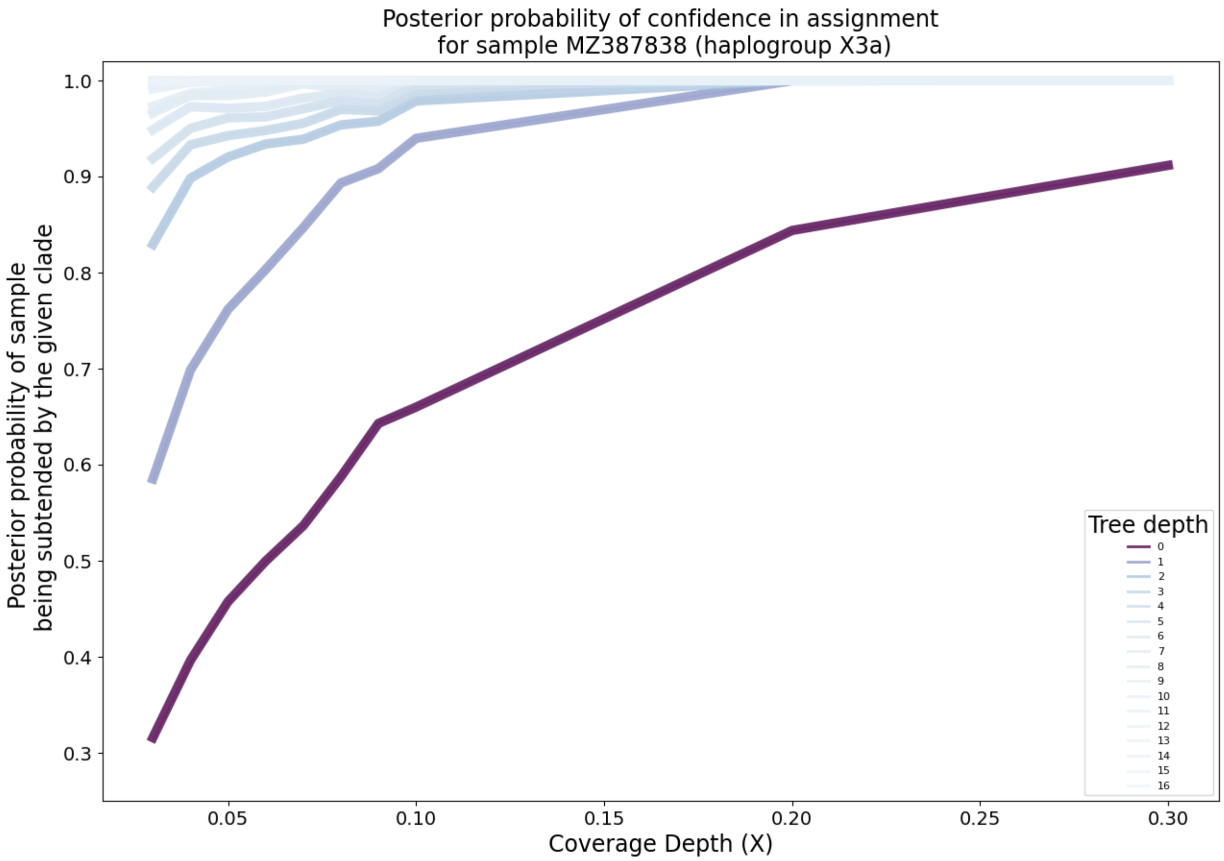

Supplementary Figure AB: **Clade-level posterior probabilities of haplogroup assignment on simulated paired-end FASTQ data.** Each lineplot represents the mean over replicates at a fixed depth on the mitochondrial tree. The darker the line, the more basal the haplogroups.

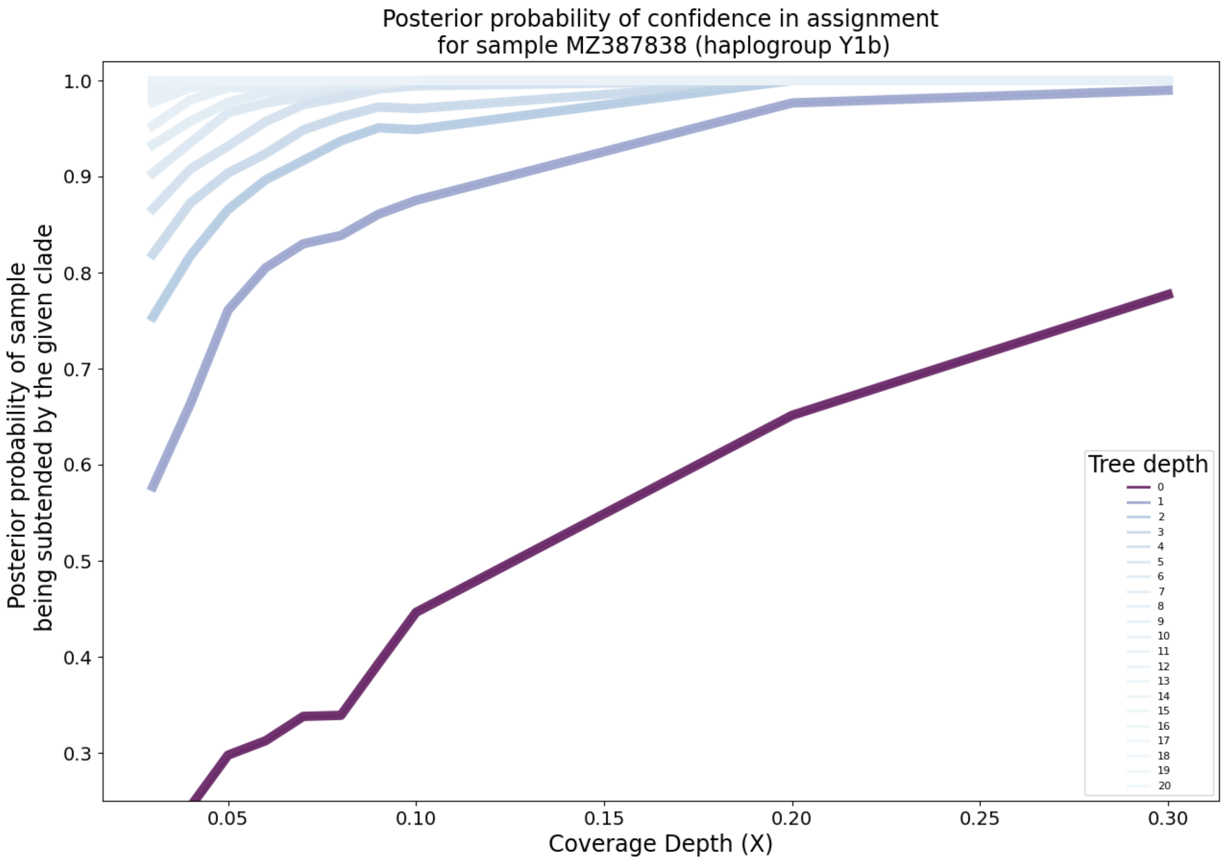

Supplementary Figure AC: **Clade-level posterior probabilities of haplogroup assignment on simulated paired-end FASTQ data.** Each lineplot represents the mean over replicates at a fixed depth on the mitochondrial tree. The darker the line, the more basal the haplogroups.

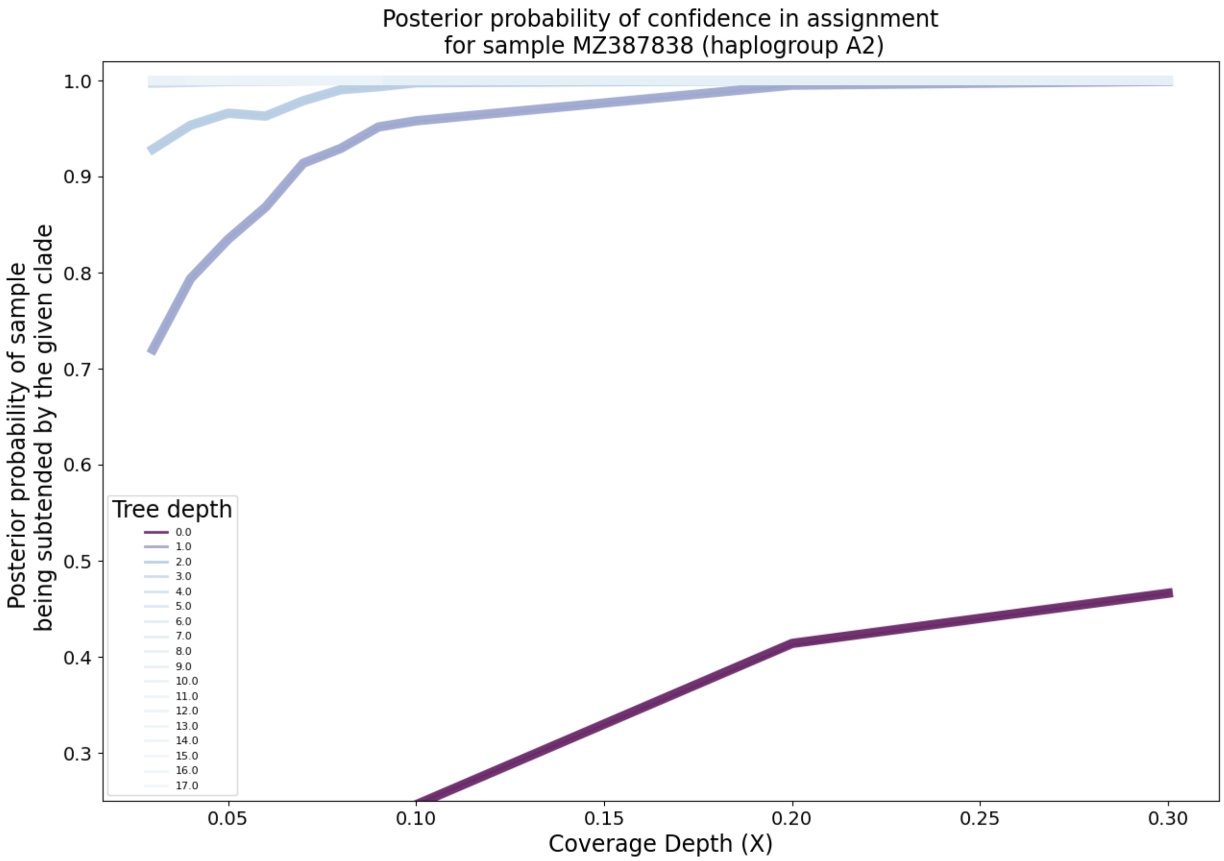

Supplementary Figure AD: **Clade-level posterior probabilities of haplogroup assignment on simulated paired-end FASTQ data with added NuMT reads.** NuMT reads were included at a rate of one in 200. Each lineplot represents the mean over replicates at a fixed depth on the mitochondrial tree. The darker the line, the more basal the haplogroups.

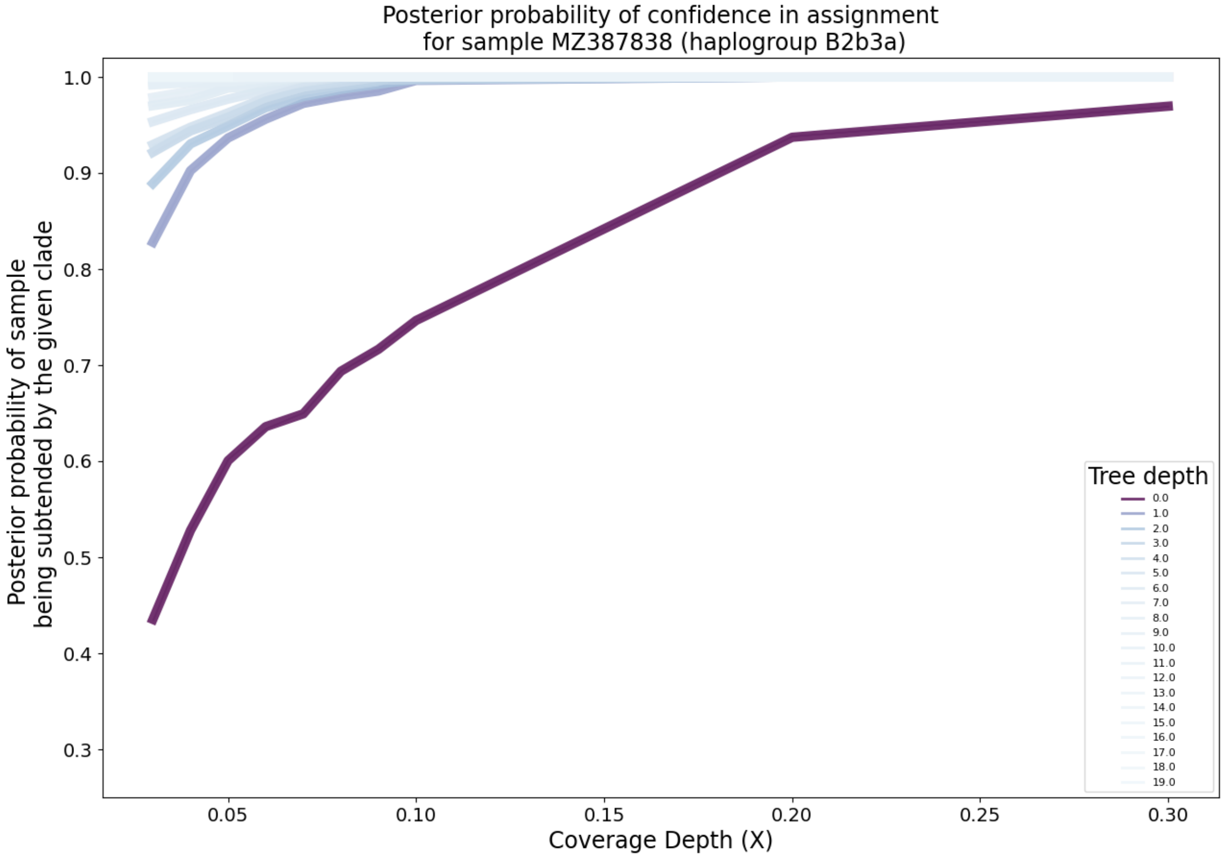

Supplementary Figure AE: **Clade-level posterior probabilities of haplogroup assignment on simulated paired-end FASTQ data with added NuMT reads (CONTINUED).** NuMT reads were included at a rate of one in 200. Each lineplot represents the mean over replicates at a fixed depth on the mitochondrial tree. The darker the line, the more basal the haplogroups.

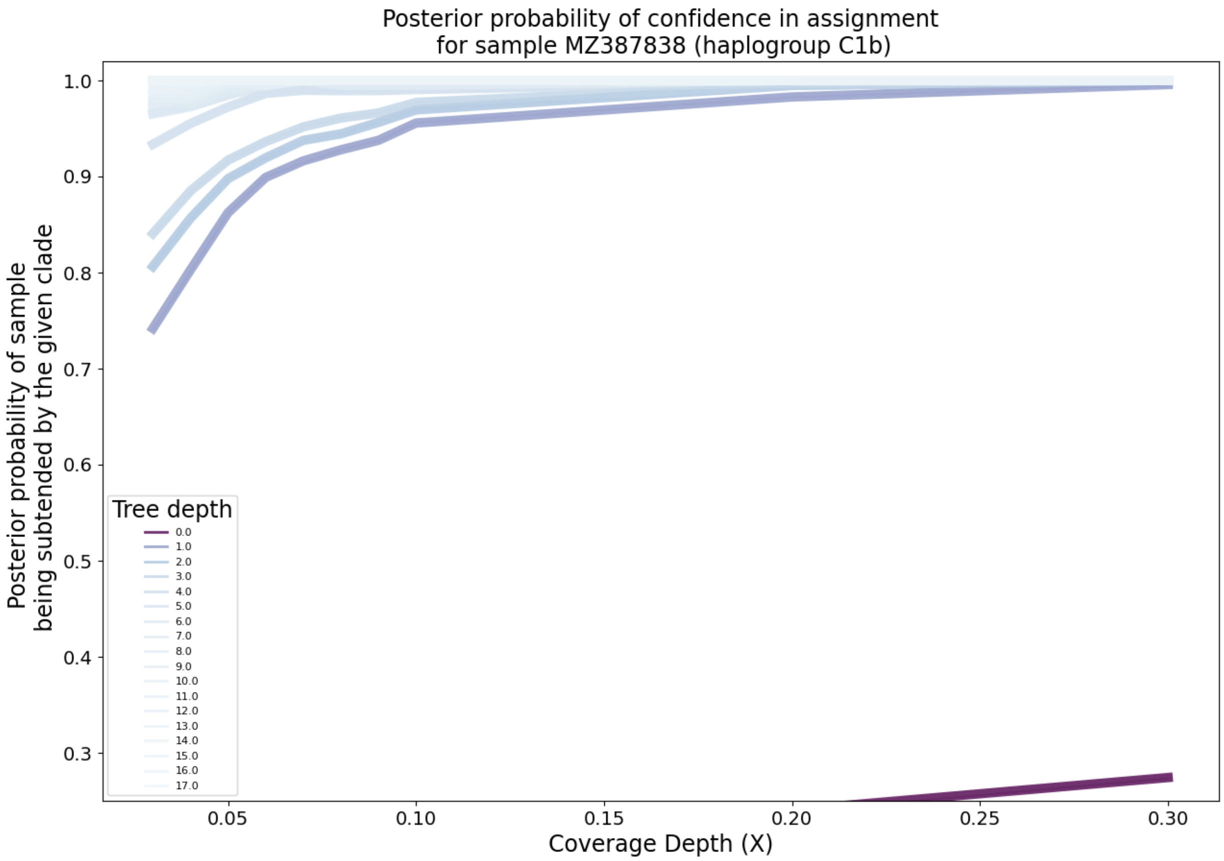

Supplementary Figure AF: **Clade-level posterior probabilities of haplogroup assignment on simulated paired-end FASTQ data with added NuMT reads (CONTINUED).** NuMT reads were included at a rate of one in 200. Each lineplot represents the mean over replicates at a fixed depth on the mitochondrial tree. The darker the line, the more basal the haplogroups.

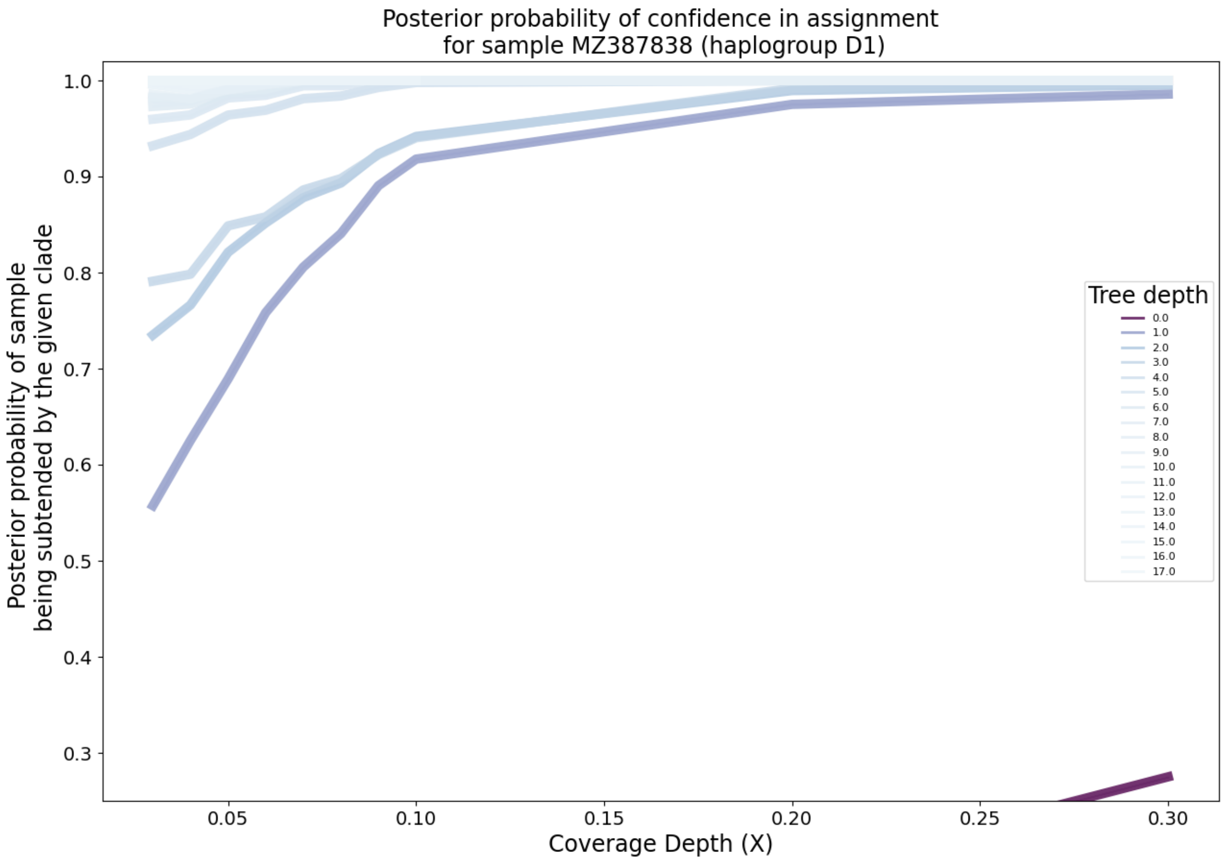

Supplementary Figure AG: **Clade-level posterior probabilities of haplogroup assignment on simulated paired-end FASTQ data with added NuMT reads (CONTINUED).** NuMT reads were included at a rate of one in 200. Each lineplot represents the mean over replicates at a fixed depth on the mitochondrial tree. The darker the line, the more basal the haplogroups.

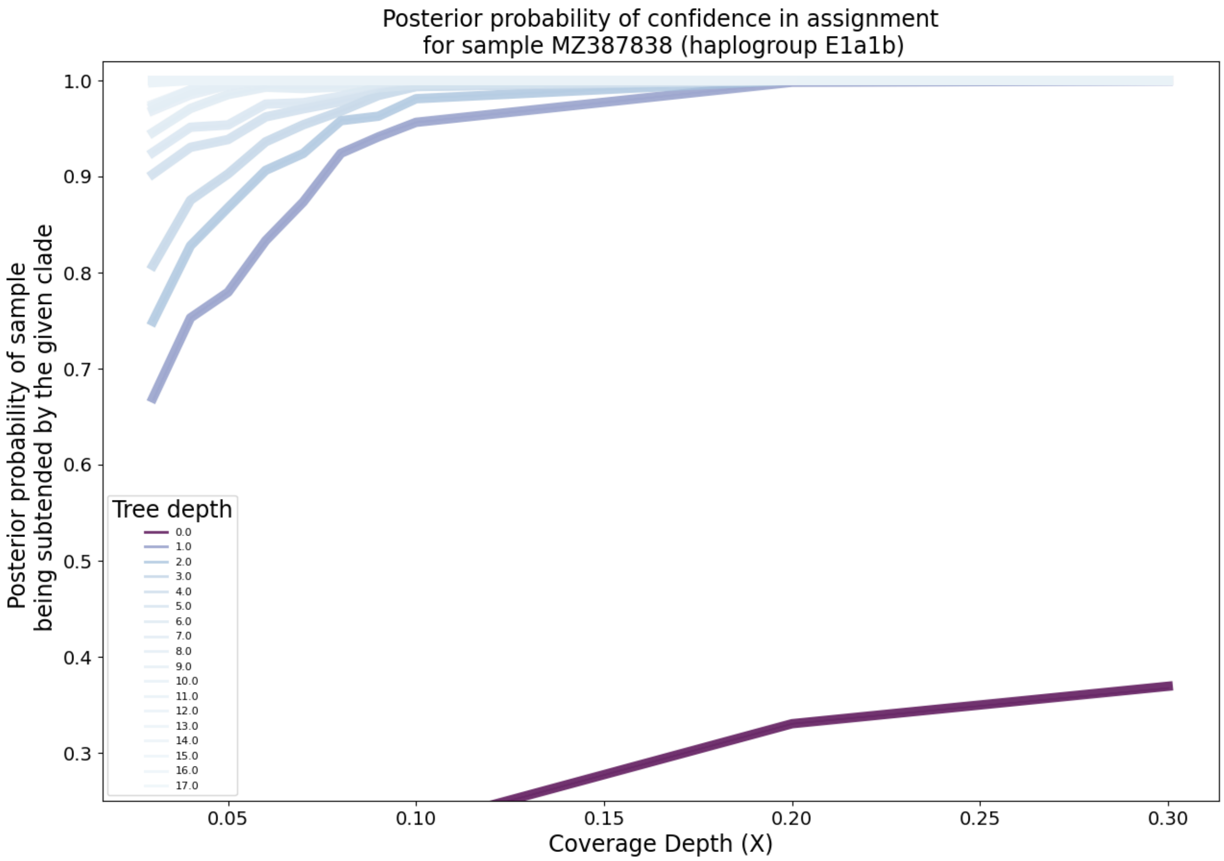

Supplementary Figure AH: **Clade-level posterior probabilities of haplogroup assignment on simulated paired-end FASTQ data with added NuMT reads (CONTINUED).** NuMT reads were included at a rate of one in 200. Each lineplot represents the mean over replicates at a fixed depth on the mitochondrial tree. The darker the line, the more basal the haplogroups.

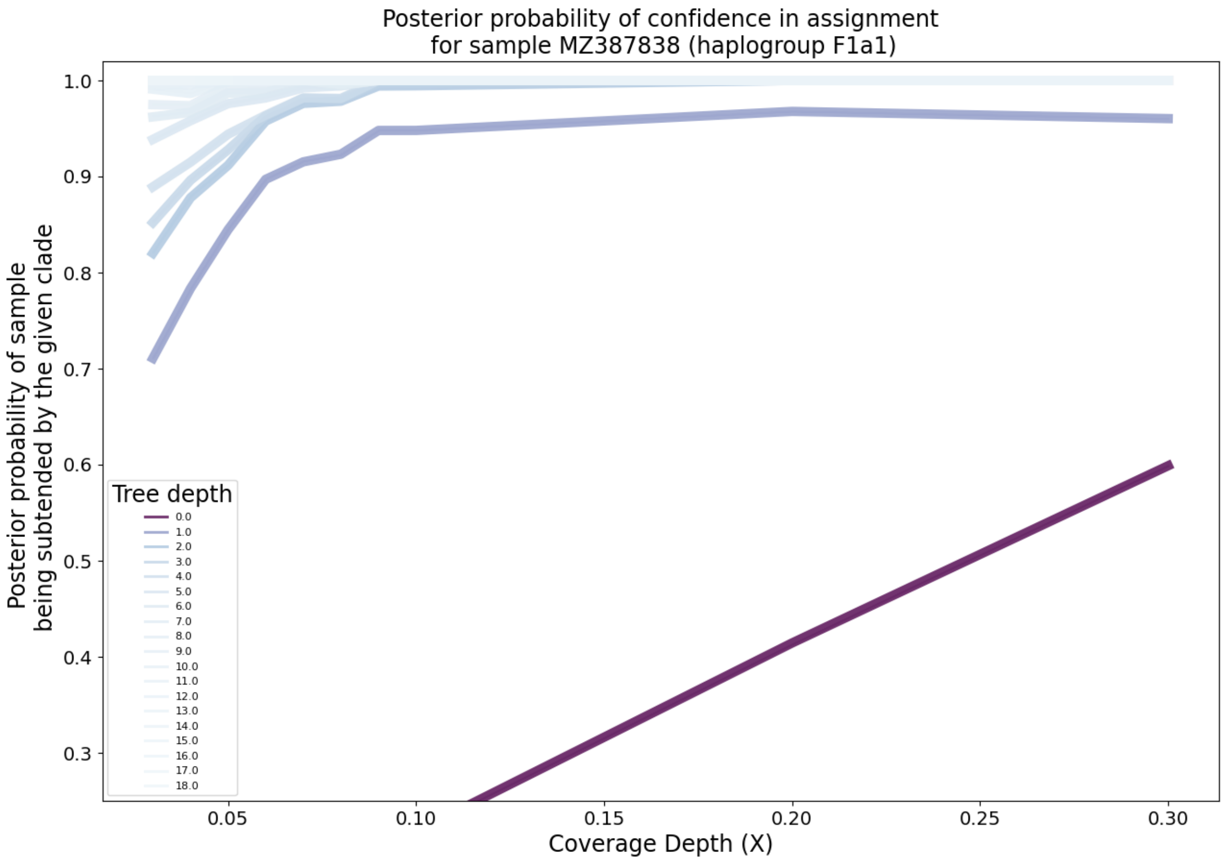

Supplementary Figure AI: **Clade-level posterior probabilities of haplogroup assignment on simulated paired-end FASTQ data with added NuMT reads (CONTINUED).** NuMT reads were included at a rate of one in 200. Each lineplot represents the mean over replicates at a fixed depth on the mitochondrial tree. The darker the line, the more basal the haplogroups.

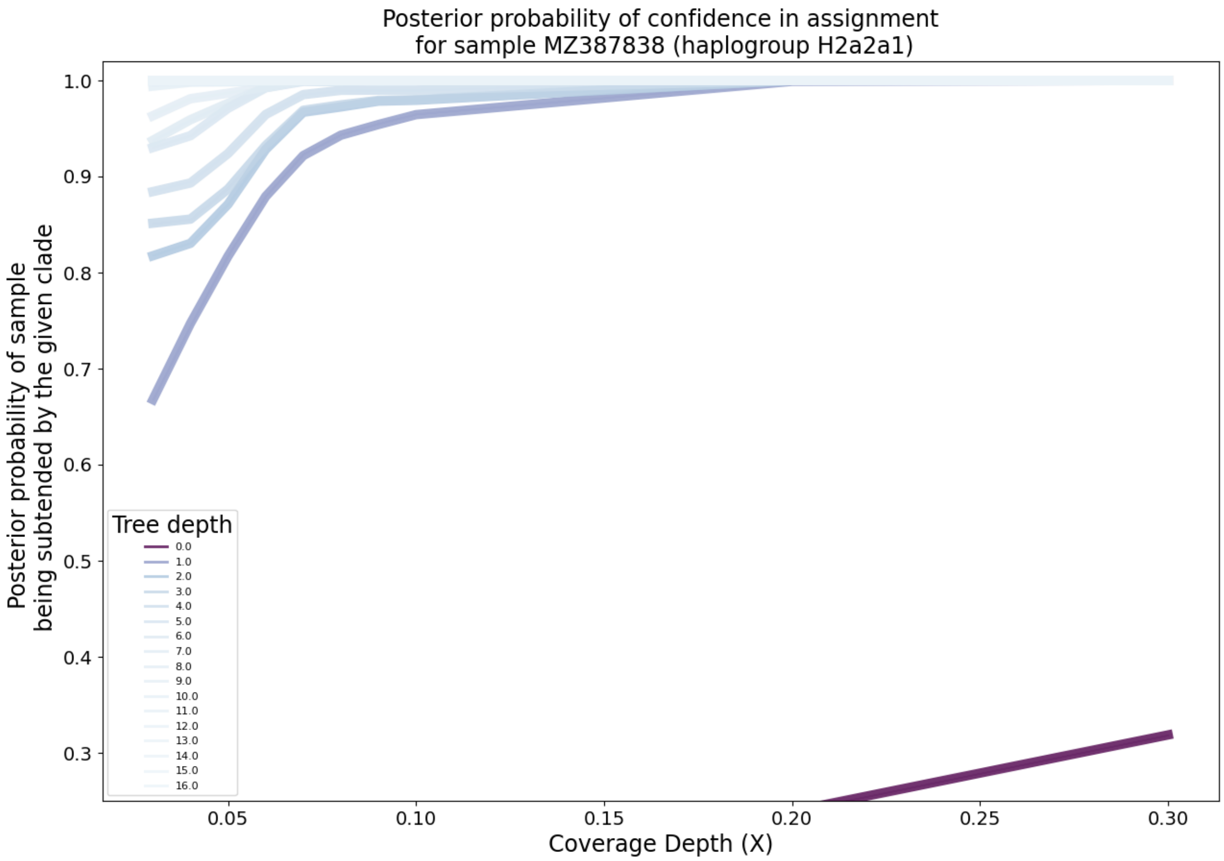

Supplementary Figure AJ: **Clade-level posterior probabilities of haplogroup assignment on simulated paired-end FASTQ data with added NuMT reads (CONTINUED).** NuMT reads were included at a rate of one in 200. Each lineplot represents the mean over replicates at a fixed depth on the mitochondrial tree. The darker the line, the more basal the haplogroups.

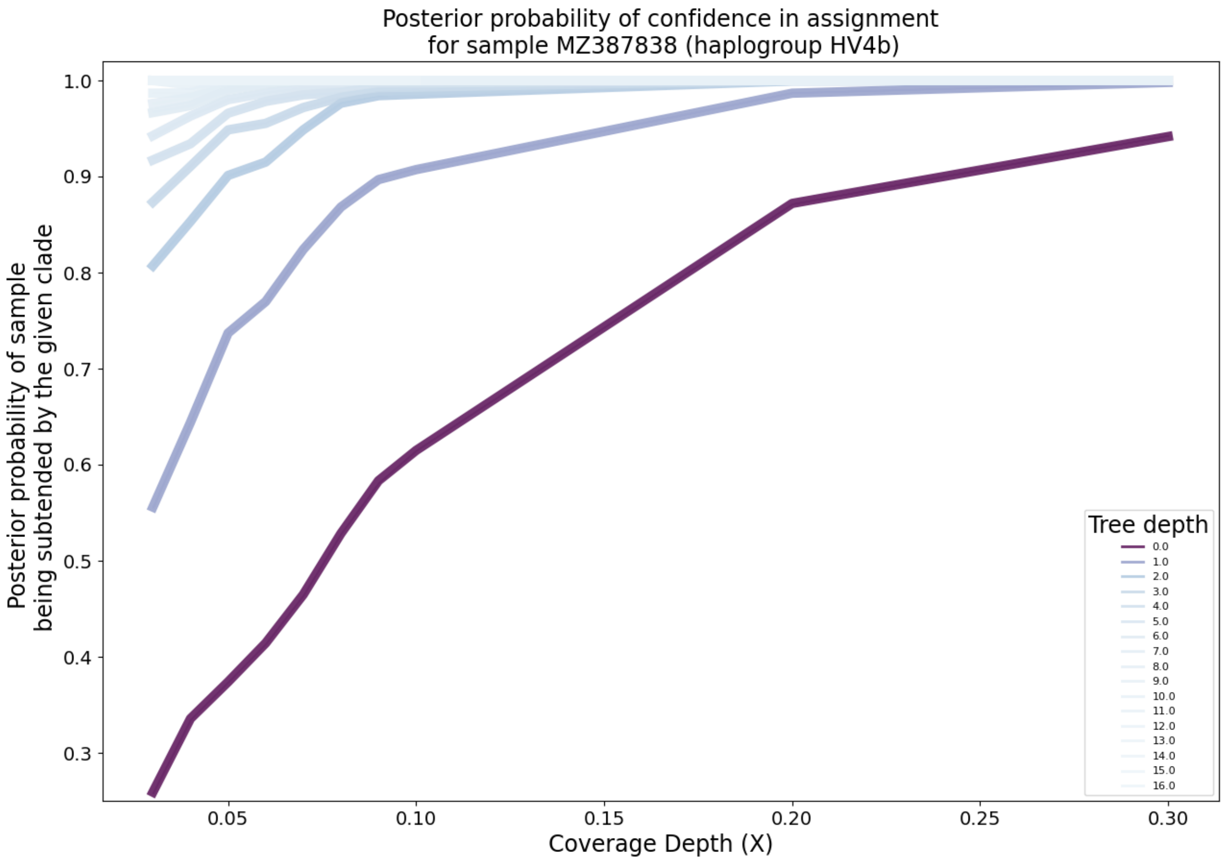

Supplementary Figure AK: **Clade-level posterior probabilities of haplogroup assignment on simulated paired-end FASTQ data with added NuMT reads (CONTINUED).** NuMT reads were included at a rate of one in 200. Each lineplot represents the mean over replicates at a fixed depth on the mitochondrial tree. The darker the line, the more basal the haplogroups.

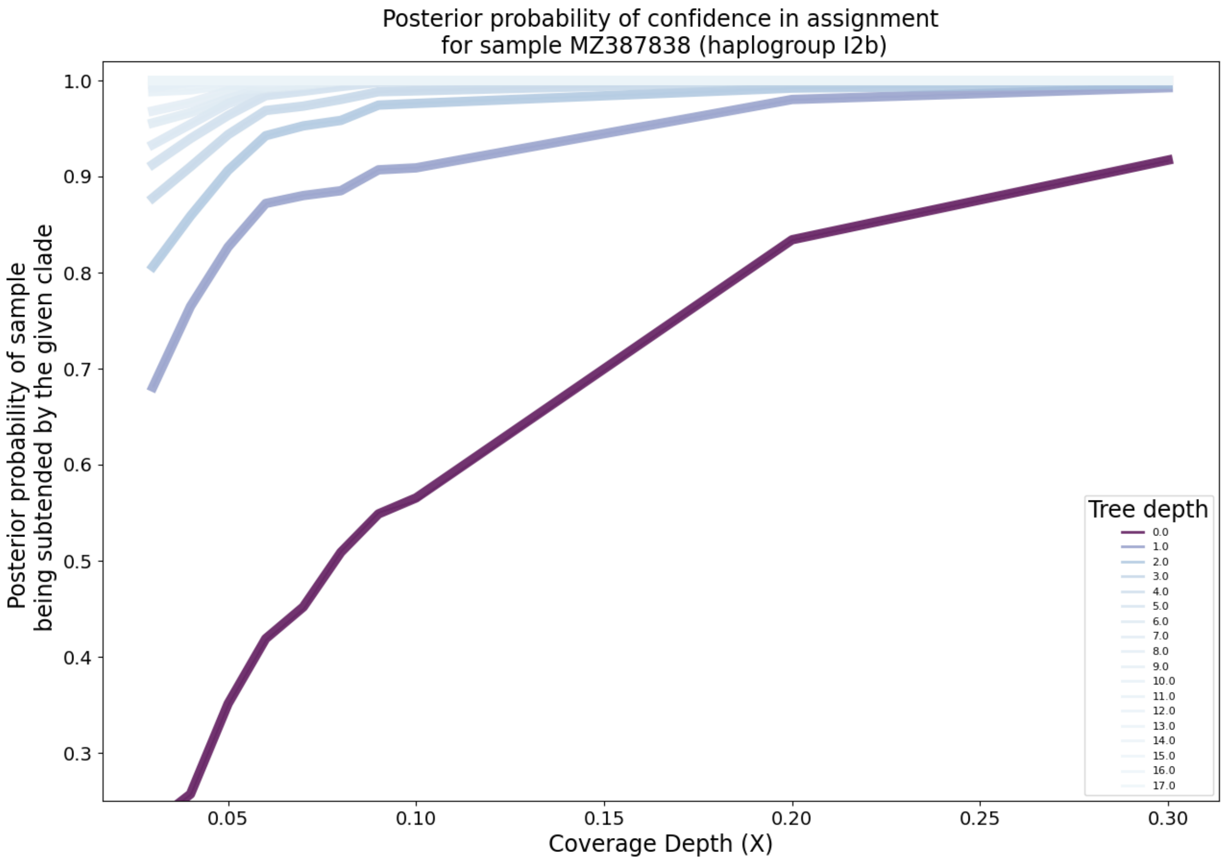

Supplementary Figure AL: **Clade-level posterior probabilities of haplogroup assignment on simulated paired-end FASTQ data with added NuMT reads (CONTINUED).** NuMT reads were included at a rate of one in 200. Each lineplot represents the mean over replicates at a fixed depth on the mitochondrial tree. The darker the line, the more basal the haplogroups.

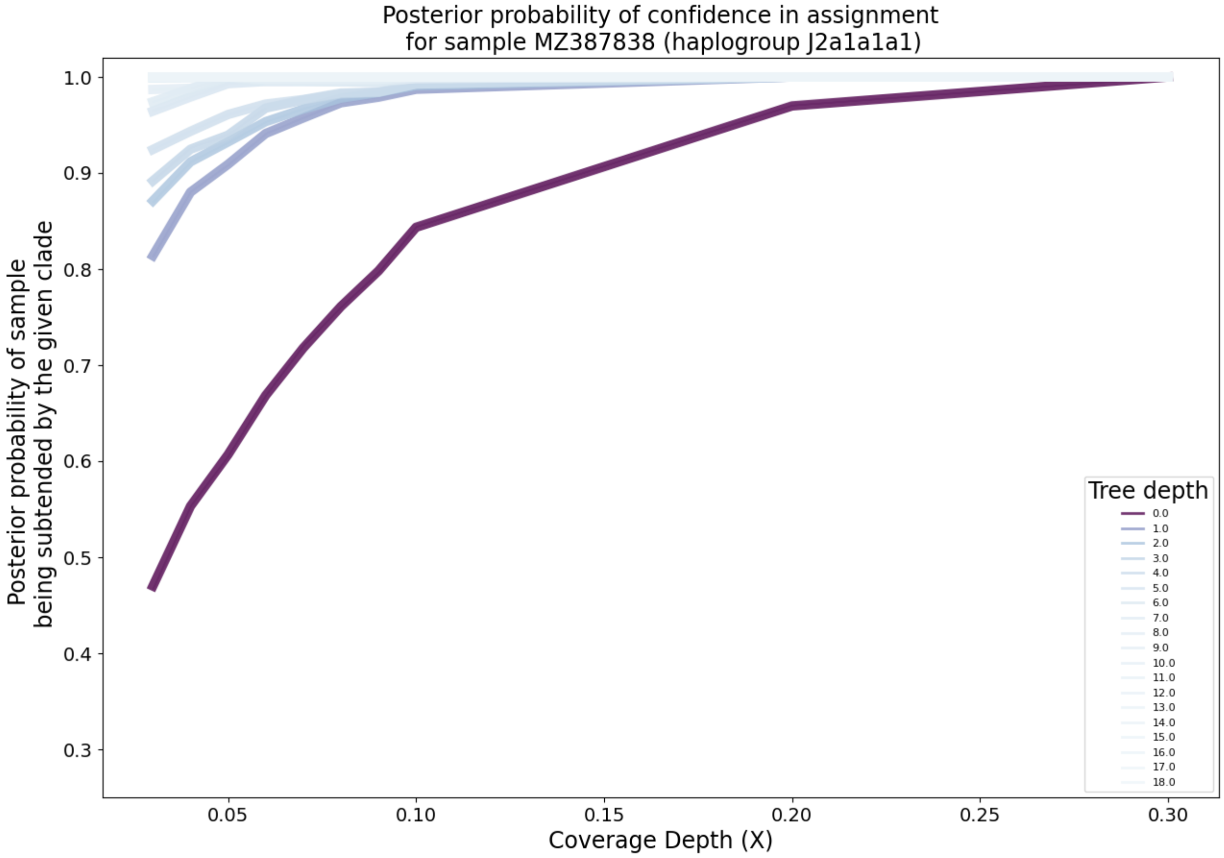

Supplementary Figure AM: **Clade-level posterior probabilities of haplogroup assignment on simulated paired-end FASTQ data with added NuMT reads (CONTINUED).** NuMT reads were included at a rate of one in 200. Each lineplot represents the mean over replicates at a fixed depth on the mitochondrial tree. The darker the line, the more basal the haplogroups.

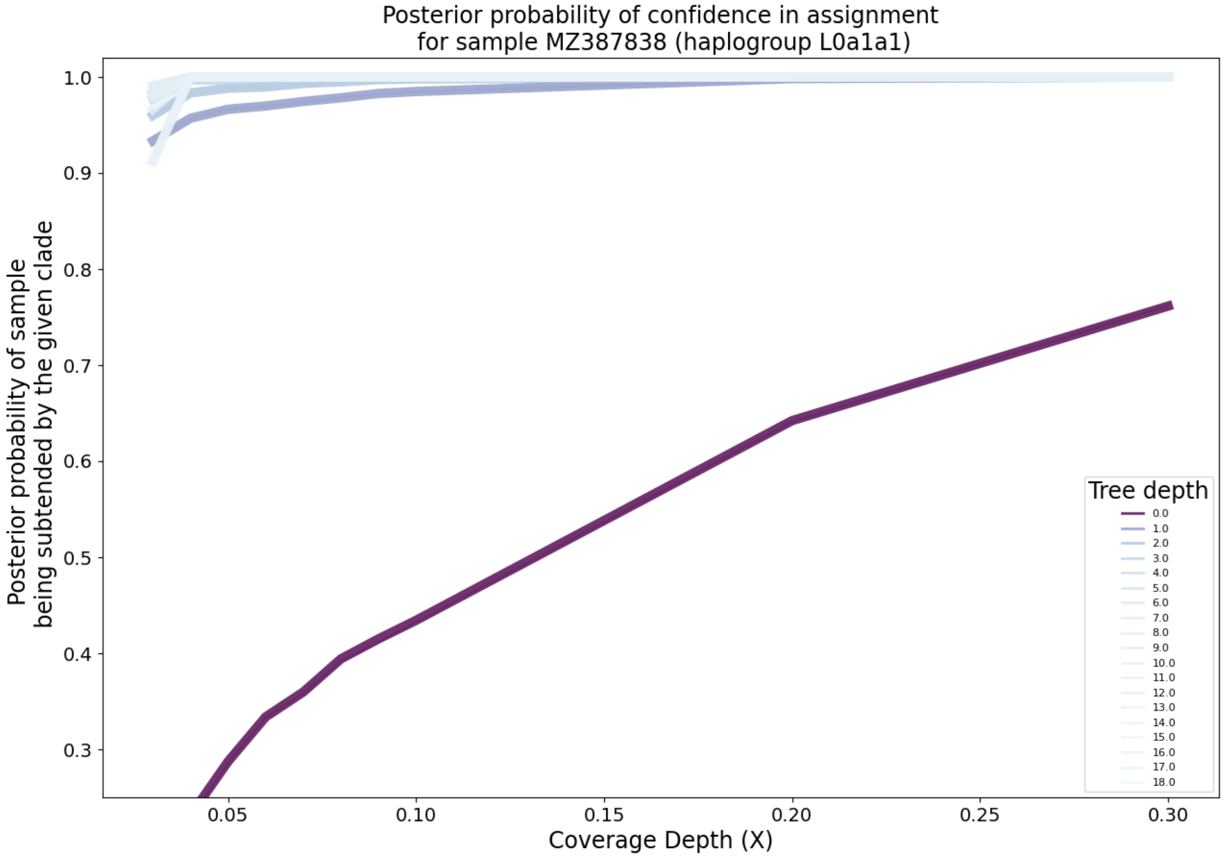

Supplementary Figure AN: **Clade-level posterior probabilities of haplogroup assignment on simulated paired-end FASTQ data with added NuMT reads (CONTINUED).** NuMT reads were included at a rate of one in 200. Each lineplot represents the mean over replicates at a fixed depth on the mitochondrial tree. The darker the line, the more basal the haplogroups.

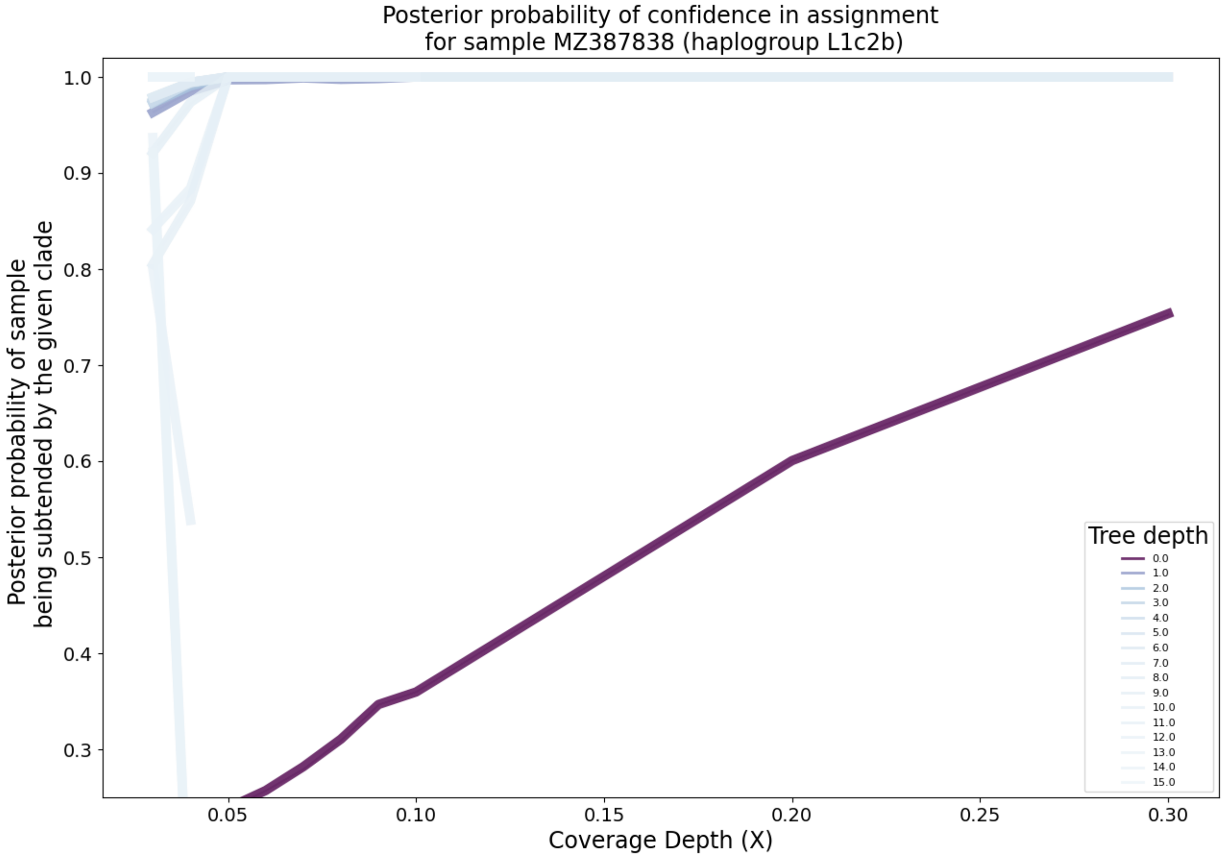

Supplementary Figure AO: **Clade-level posterior probabilities of haplogroup assignment on simulated paired-end FASTQ data with added NuMT reads (CONTINUED).** NuMT reads were included at a rate of one in 200. Each lineplot represents the mean over replicates at a fixed depth on the mitochondrial tree. The darker the line, the more basal the haplogroups.

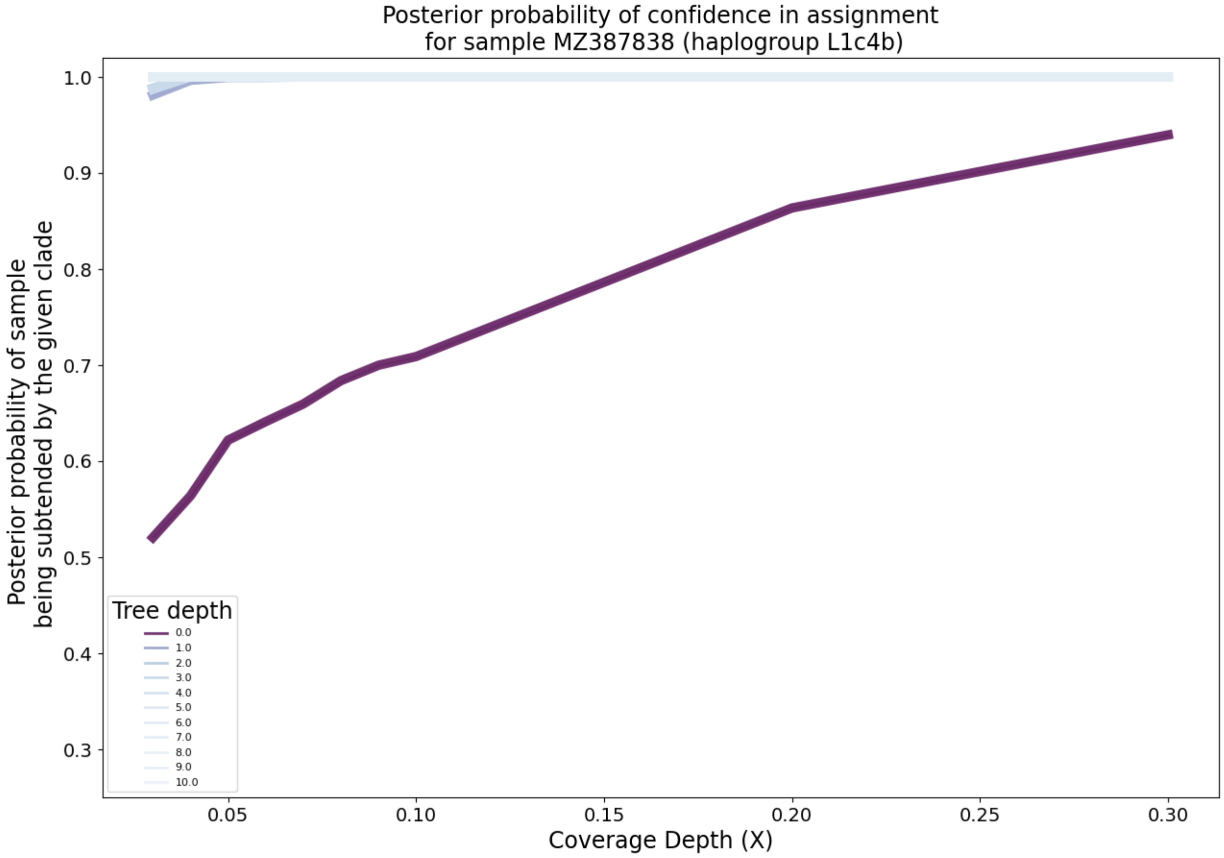

Supplementary Figure AP: **Clade-level posterior probabilities of haplogroup assignment on simulated paired-end FASTQ data with added NuMT reads (CONTINUED).** NuMT reads were included at a rate of one in 200. Each lineplot represents the mean over replicates at a fixed depth on the mitochondrial tree. The darker the line, the more basal the haplogroups.

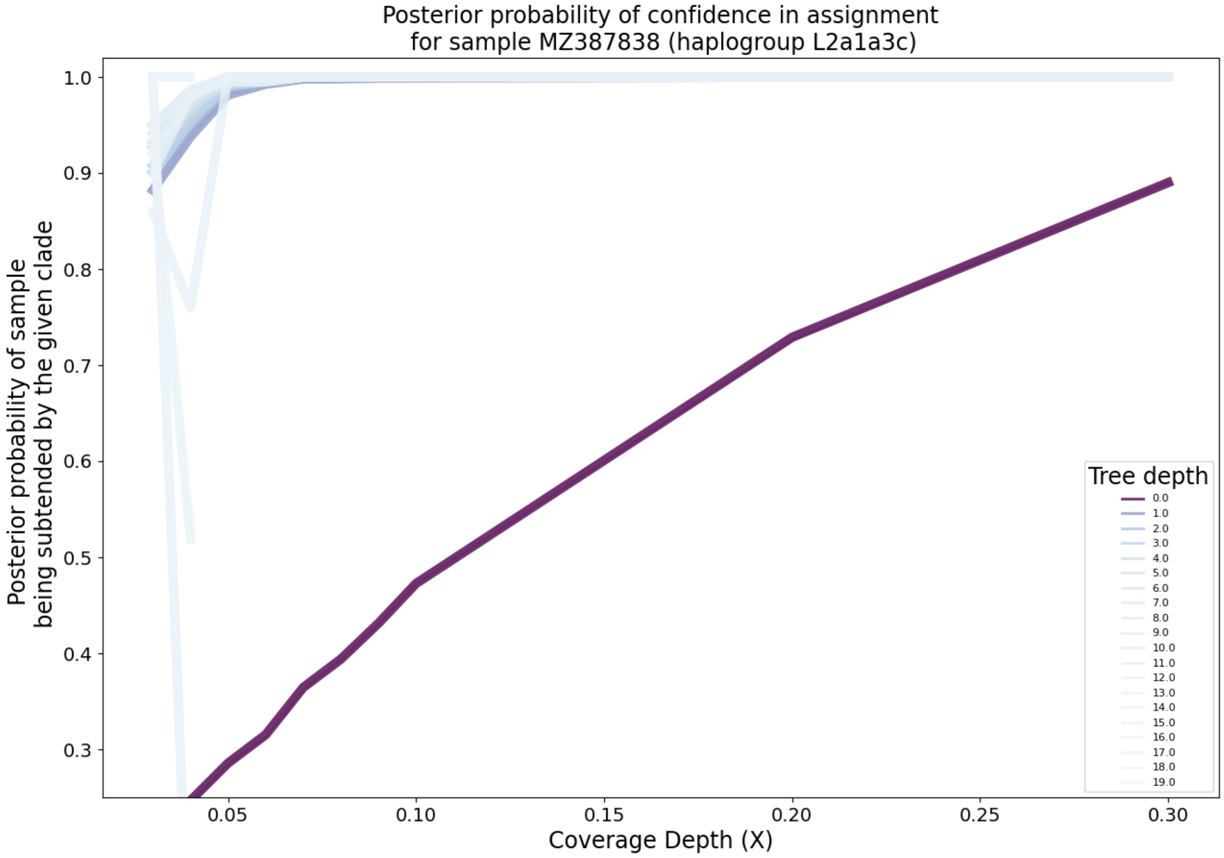

Supplementary Figure AQ: **Clade-level posterior probabilities of haplogroup assignment on simulated paired-end FASTQ data with added NuMT reads (CONTINUED).** NuMT reads were included at a rate of one in 200. Each lineplot represents the mean over replicates at a fixed depth on the mitochondrial tree. The darker the line, the more basal the haplogroups.

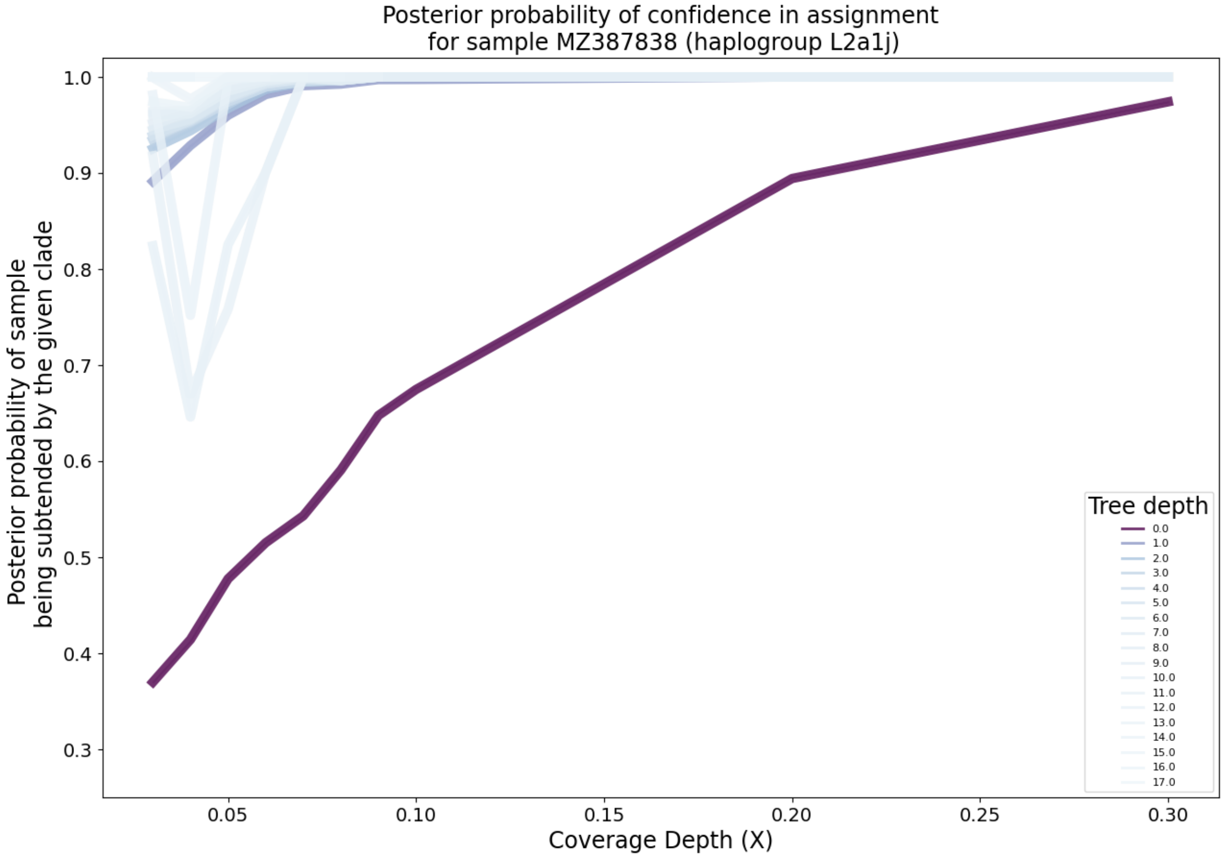

Supplementary Figure AR: **Clade-level posterior probabilities of haplogroup assignment on simulated paired-end FASTQ data with added NuMT reads (CONTINUED).** NuMT reads were included at a rate of one in 200. Each lineplot represents the mean over replicates at a fixed depth on the mitochondrial tree. The darker the line, the more basal the haplogroups.

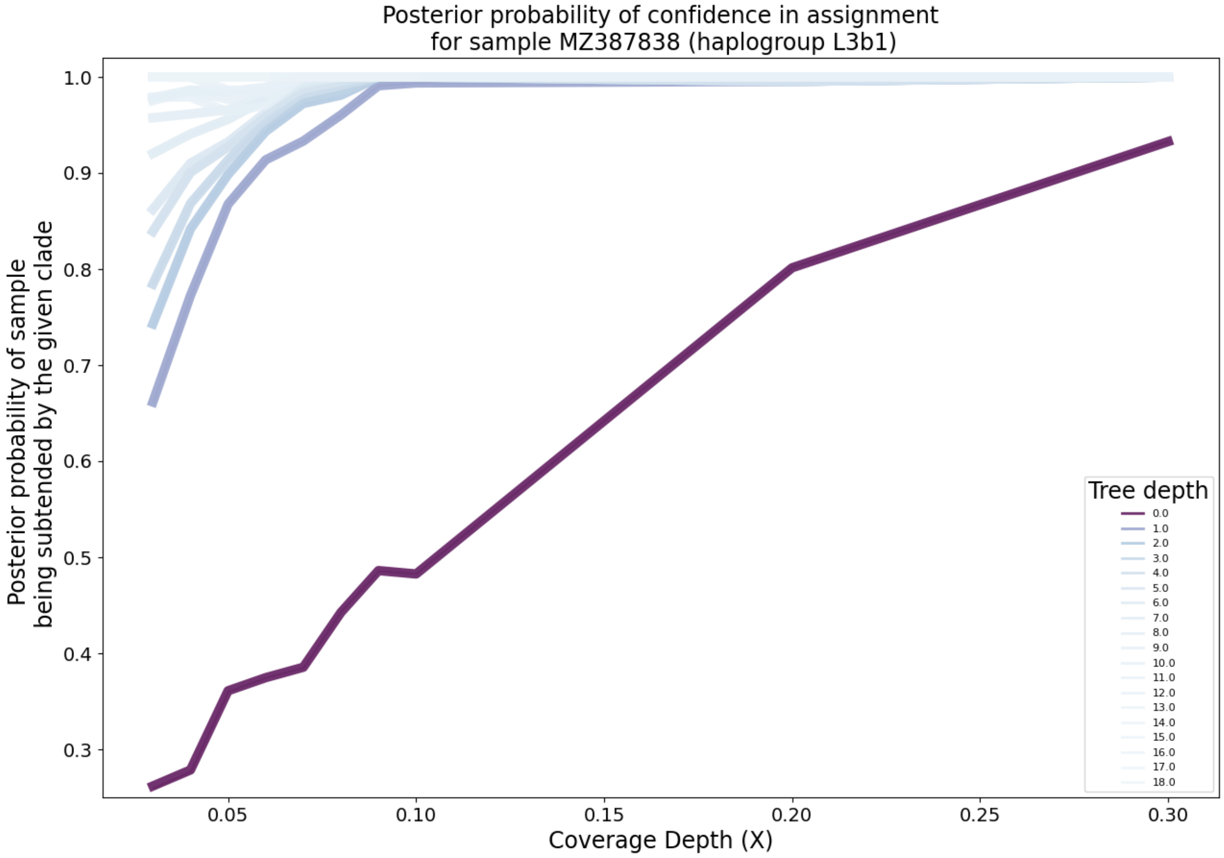

Supplementary Figure AS: **Clade-level posterior probabilities of haplogroup assignment on simulated paired-end FASTQ data with added NuMT reads (CONTINUED).** NuMT reads were included at a rate of one in 200. Each lineplot represents the mean over replicates at a fixed depth on the mitochondrial tree. The darker the line, the more basal the haplogroups.

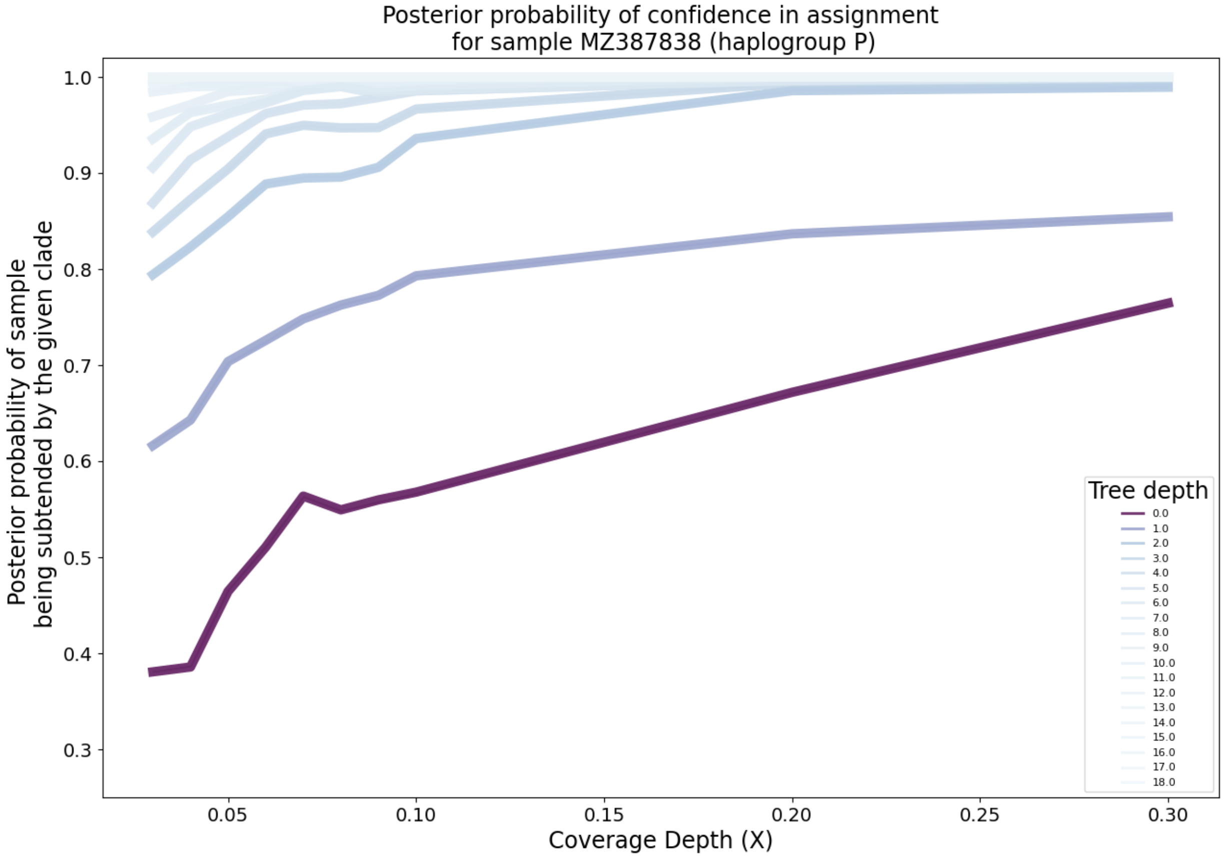

Supplementary Figure AT: **Clade-level posterior probabilities of haplogroup assignment on simulated paired-end FASTQ data with added NuMT reads (CONTINUED).** NuMT reads were included at a rate of one in 200. Each lineplot represents the mean over replicates at a fixed depth on the mitochondrial tree. The darker the line, the more basal the haplogroups.

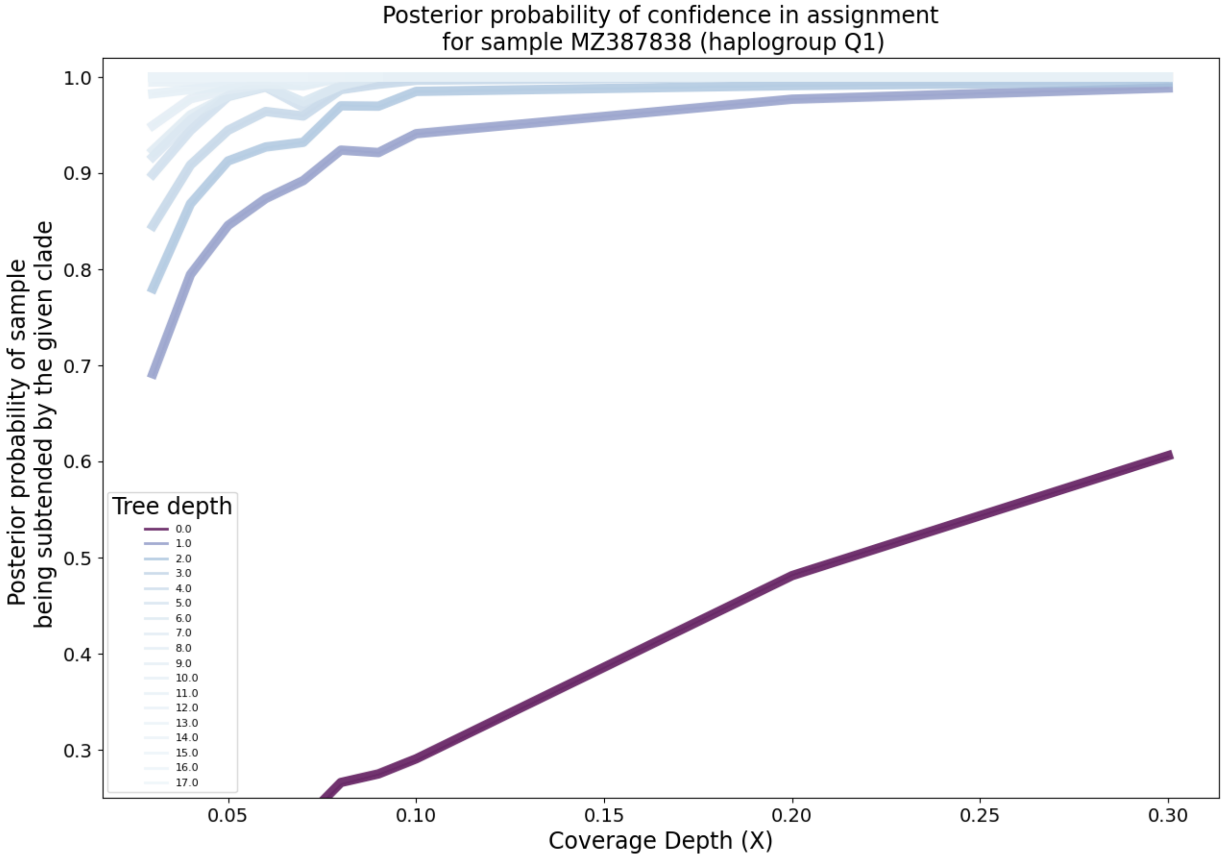

Supplementary Figure AU: **Clade-level posterior probabilities of haplogroup assignment on simulated paired-end FASTQ data with added NuMT reads (CONTINUED).** NuMT reads were included at a rate of one in 200. Each lineplot represents the mean over replicates at a fixed depth on the mitochondrial tree. The darker the line, the more basal the haplogroups.

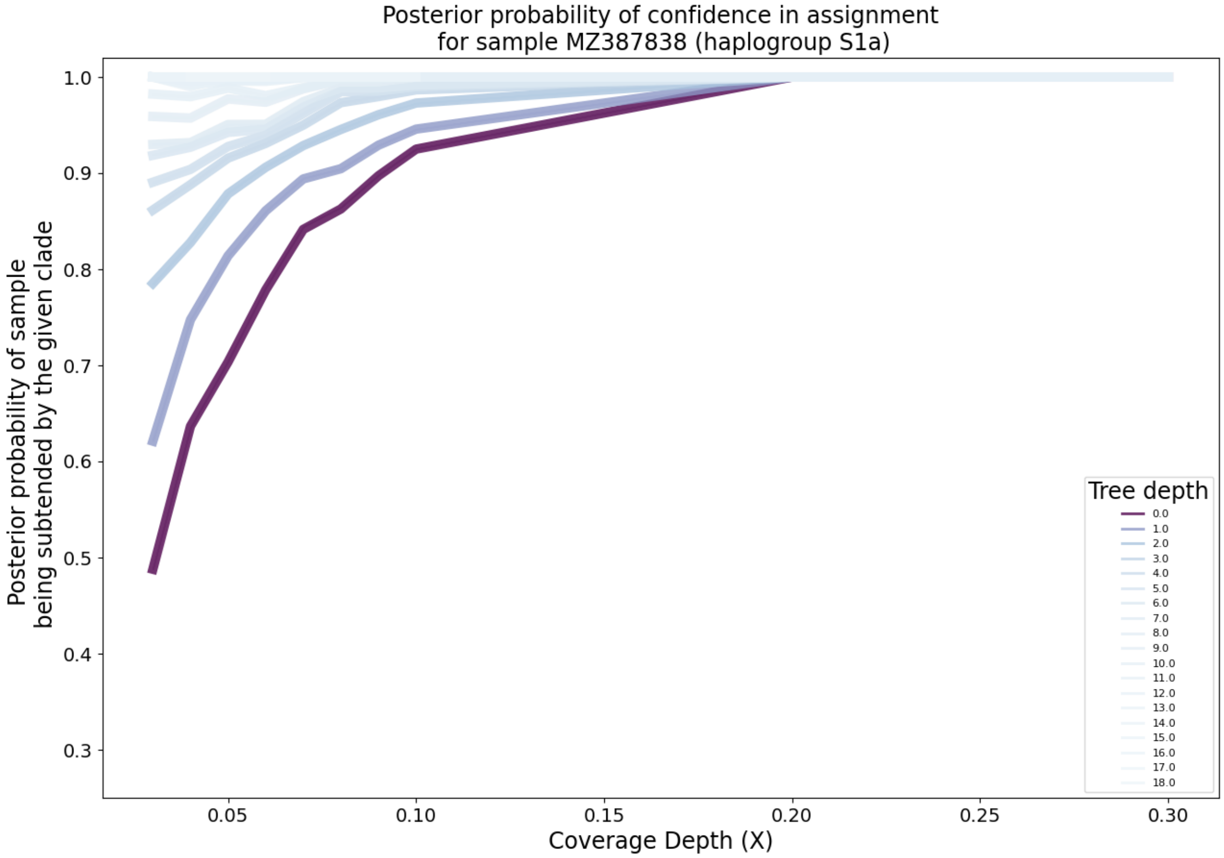

Supplementary Figure AV: **Clade-level posterior probabilities of haplogroup assignment on simulated paired-end FASTQ data with added NuMT reads (CONTINUED).** NuMT reads were included at a rate of one in 200. Each lineplot represents the mean over replicates at a fixed depth on the mitochondrial tree. The darker the line, the more basal the haplogroups.

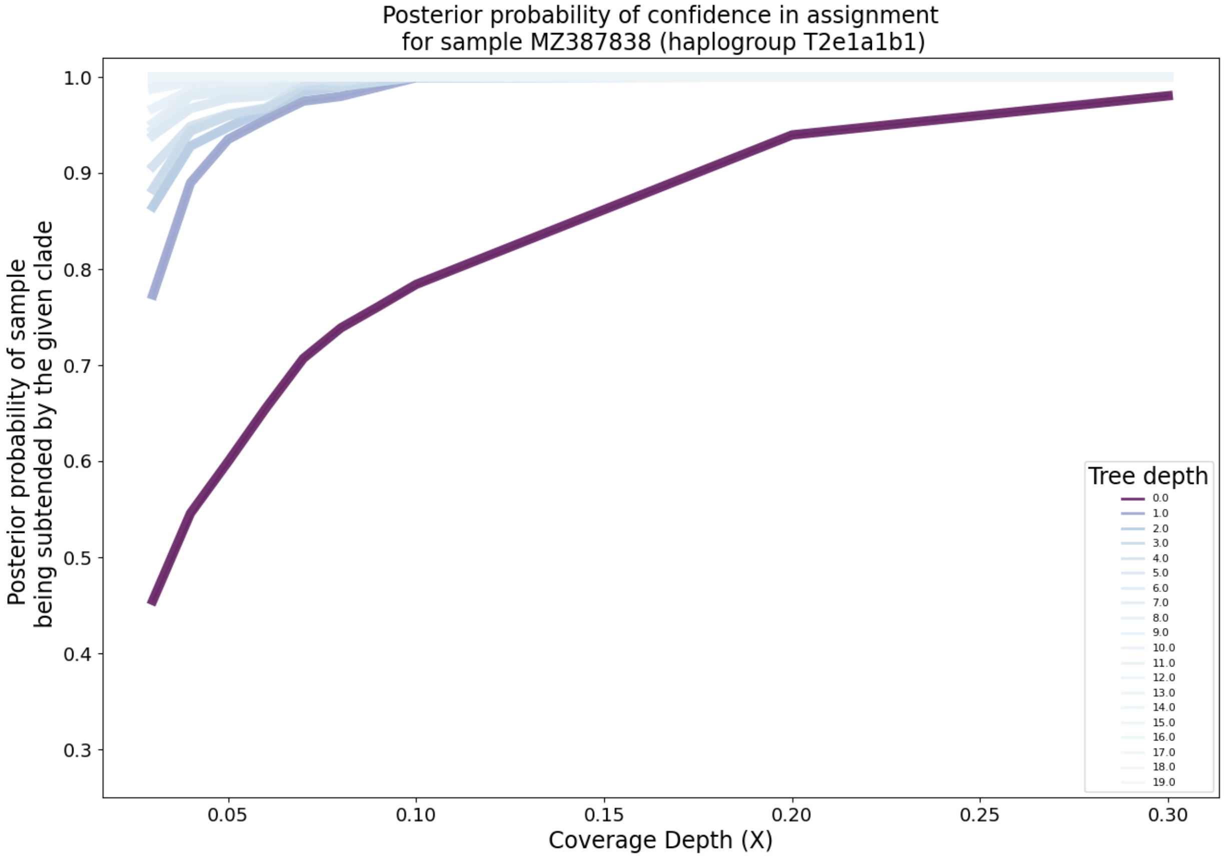

Supplementary Figure AW: **Clade-level posterior probabilities of haplogroup assignment on simulated paired-end FASTQ data with added NuMT reads (CONTINUED).** NuMT reads were included at a rate of one in 200. Each lineplot represents the mean over replicates at a fixed depth on the mitochondrial tree. The darker the line, the more basal the haplogroups.

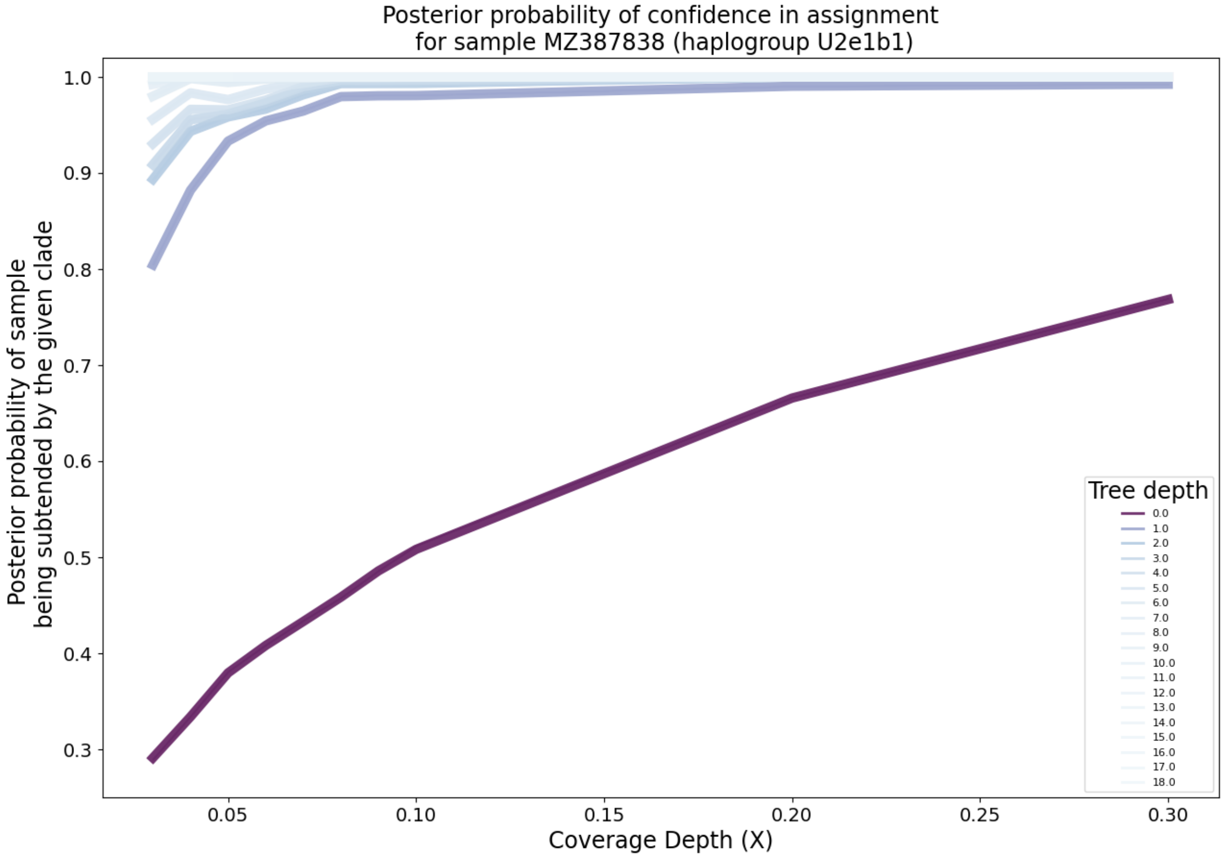

Supplementary Figure AX: **Clade-level posterior probabilities of haplogroup assignment on simulated paired-end FASTQ data with added NuMT reads (CONTINUED).** NuMT reads were included at a rate of one in 200. Each lineplot represents the mean over replicates at a fixed depth on the mitochondrial tree. The darker the line, the more basal the haplogroups.

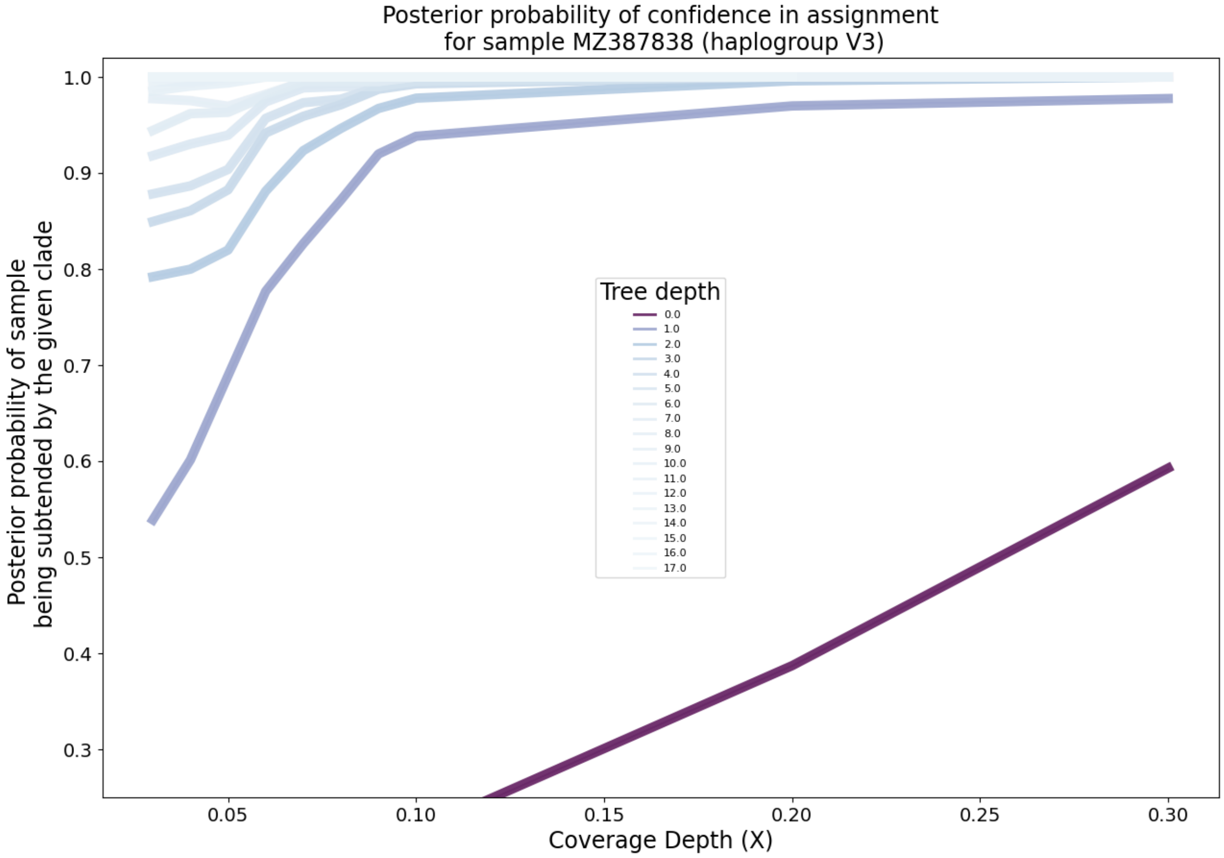

Supplementary Figure AY: **Clade-level posterior probabilities of haplogroup assignment on simulated paired-end FASTQ data with added NuMT reads (CONTINUED).** NuMT reads were included at a rate of one in 200. Each lineplot represents the mean over replicates at a fixed depth on the mitochondrial tree. The darker the line, the more basal the haplogroups.

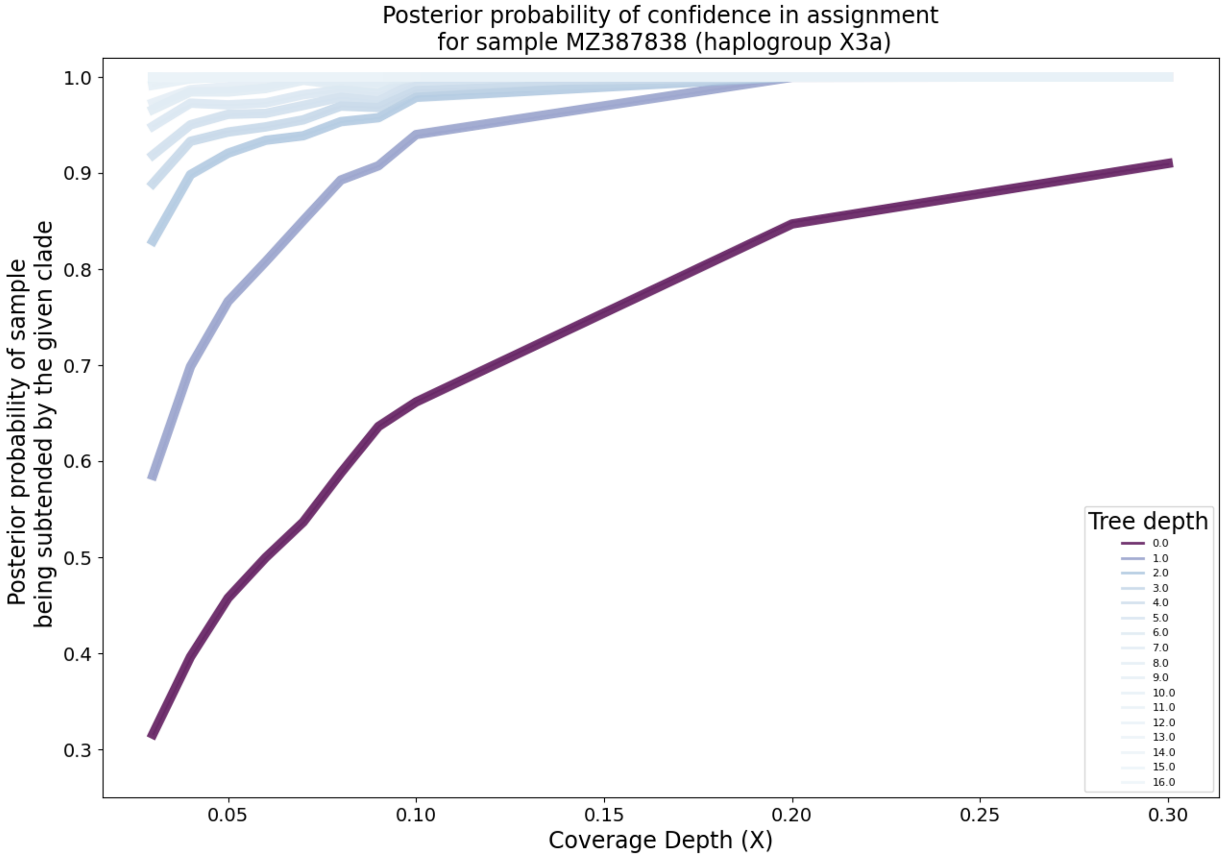

Supplementary Figure AZ: **Clade-level posterior probabilities of haplogroup assignment on simulated paired-end FASTQ data with added NuMT reads (CONTINUED).** NuMT reads were included at a rate of one in 200. Each lineplot represents the mean over replicates at a fixed depth on the mitochondrial tree. The darker the line, the more basal the haplogroups.

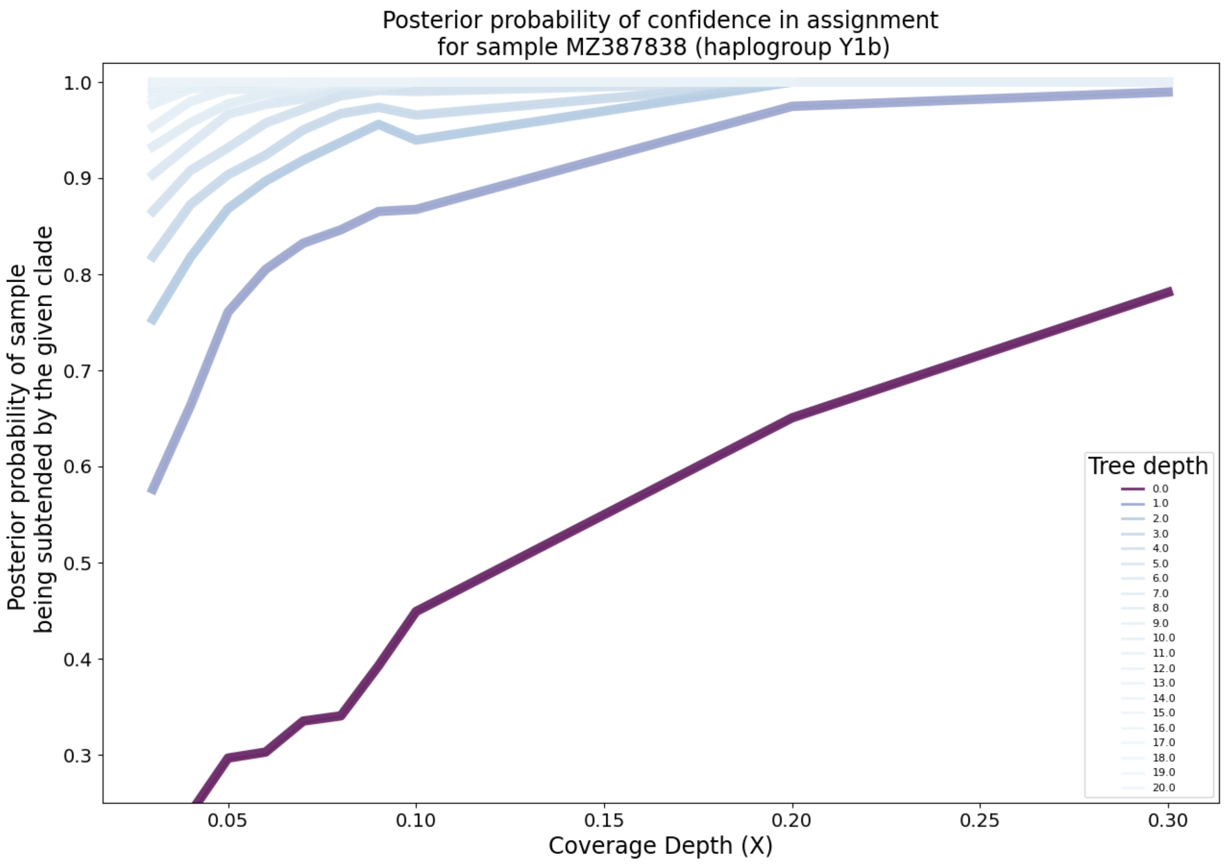

Supplementary Figure BA: **Clade-level posterior probabilities of haplogroup assignment on simulated paired-end FASTQ data with added NuMT reads (CONTINUED).** NuMT reads were included at a rate of one in 200. Each lineplot represents the mean over replicates at a fixed depth on the mitochondrial tree. The darker the line, the more basal the haplogroups.

2.4 Empirical Paired-end FASTQ Posterior Plots

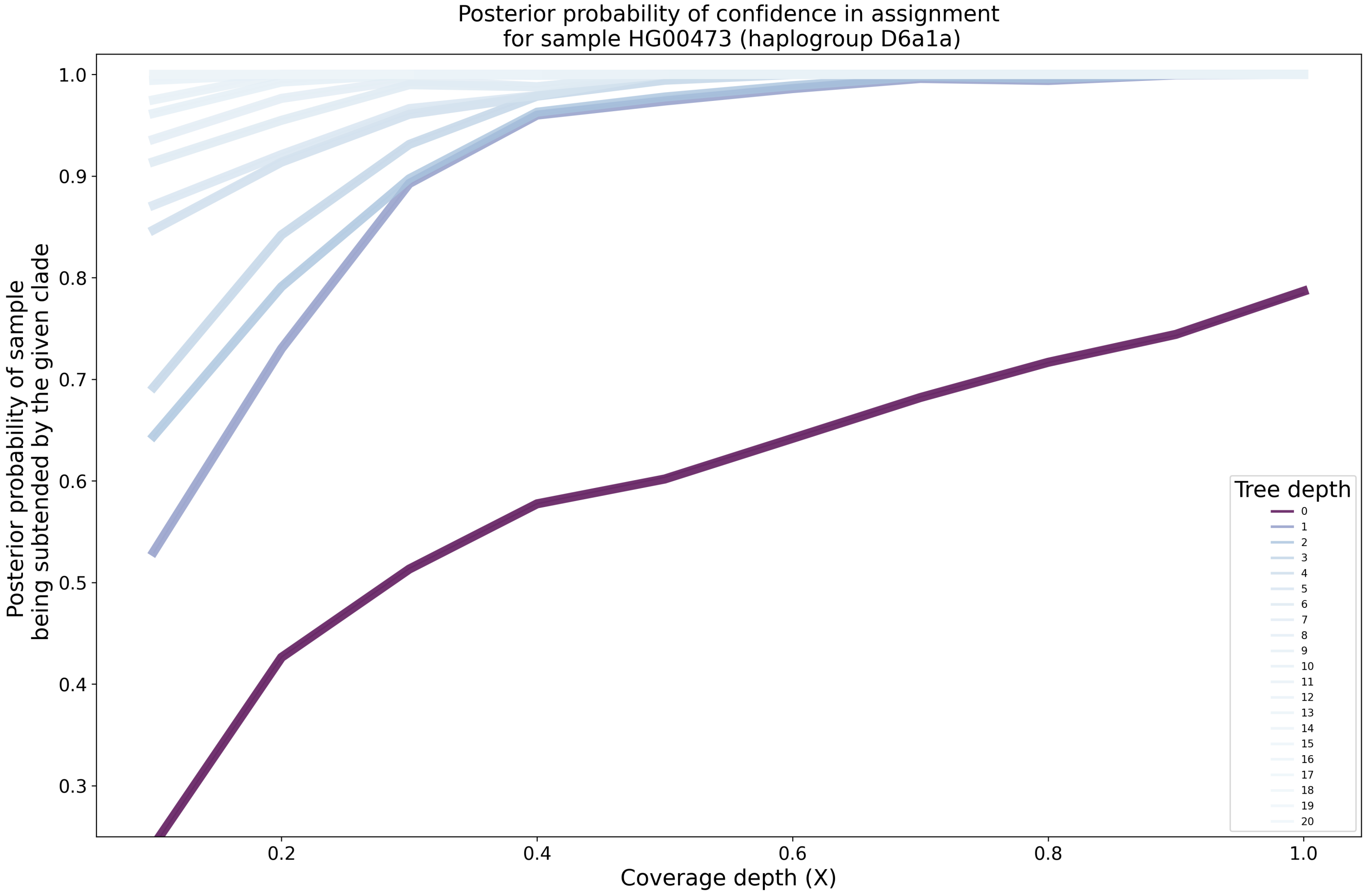

Supplementary Figure BB: **Clade-level posterior probabilities on empirical paired-end FASTQ data.** Each lineplot represents the mean over replicates at a fixed depth on the mitochondrial tree. The darker the line, the more basal the haplogroups.

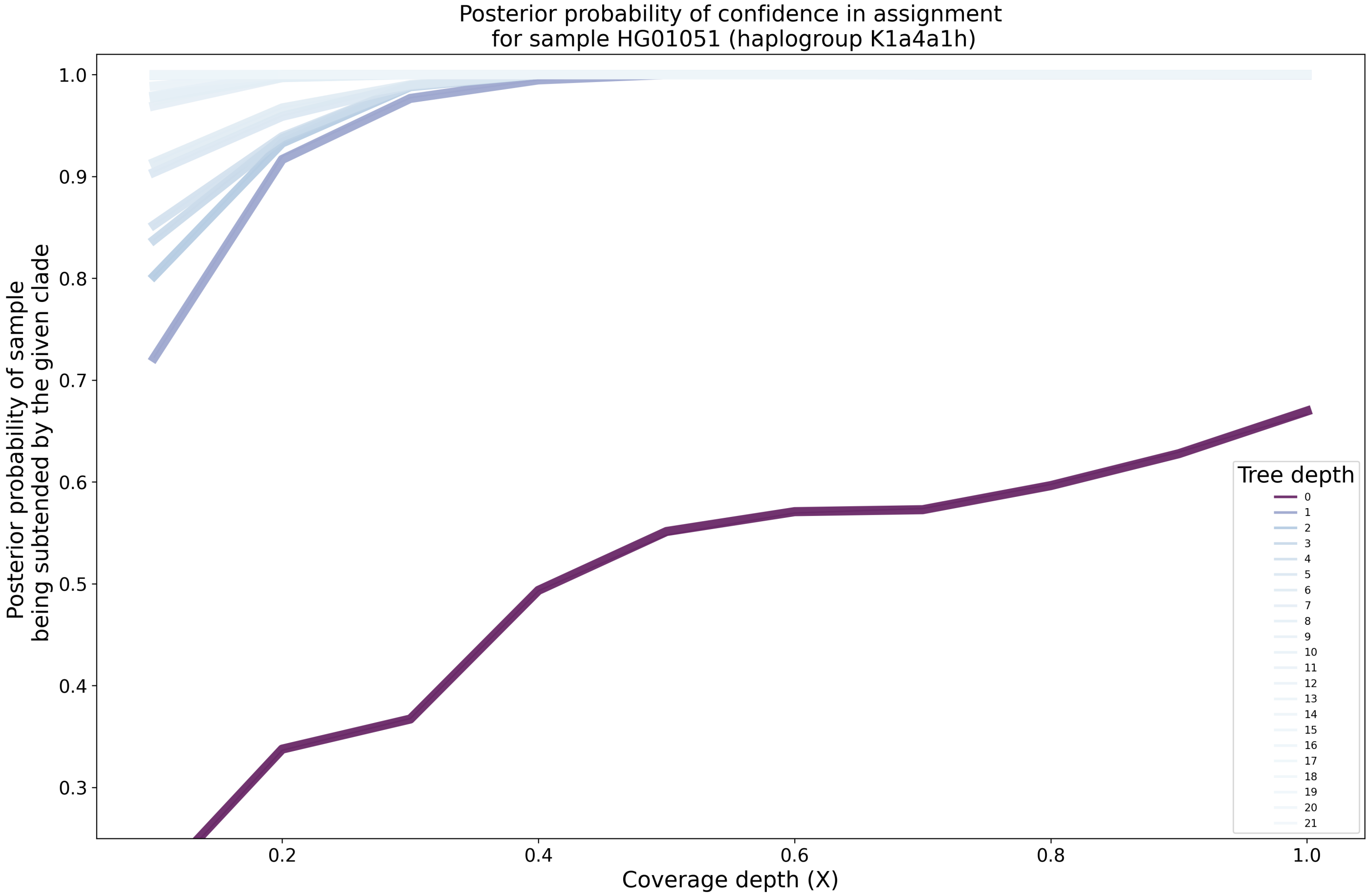

Supplementary Figure BC: **Clade-level posterior probabilities on empirical paired-end FASTQ data (CONTINUED).** Each lineplot represents the mean over replicates at a fixed depth on the mitochondrial tree. The darker the line, the more basal the haplogroups.

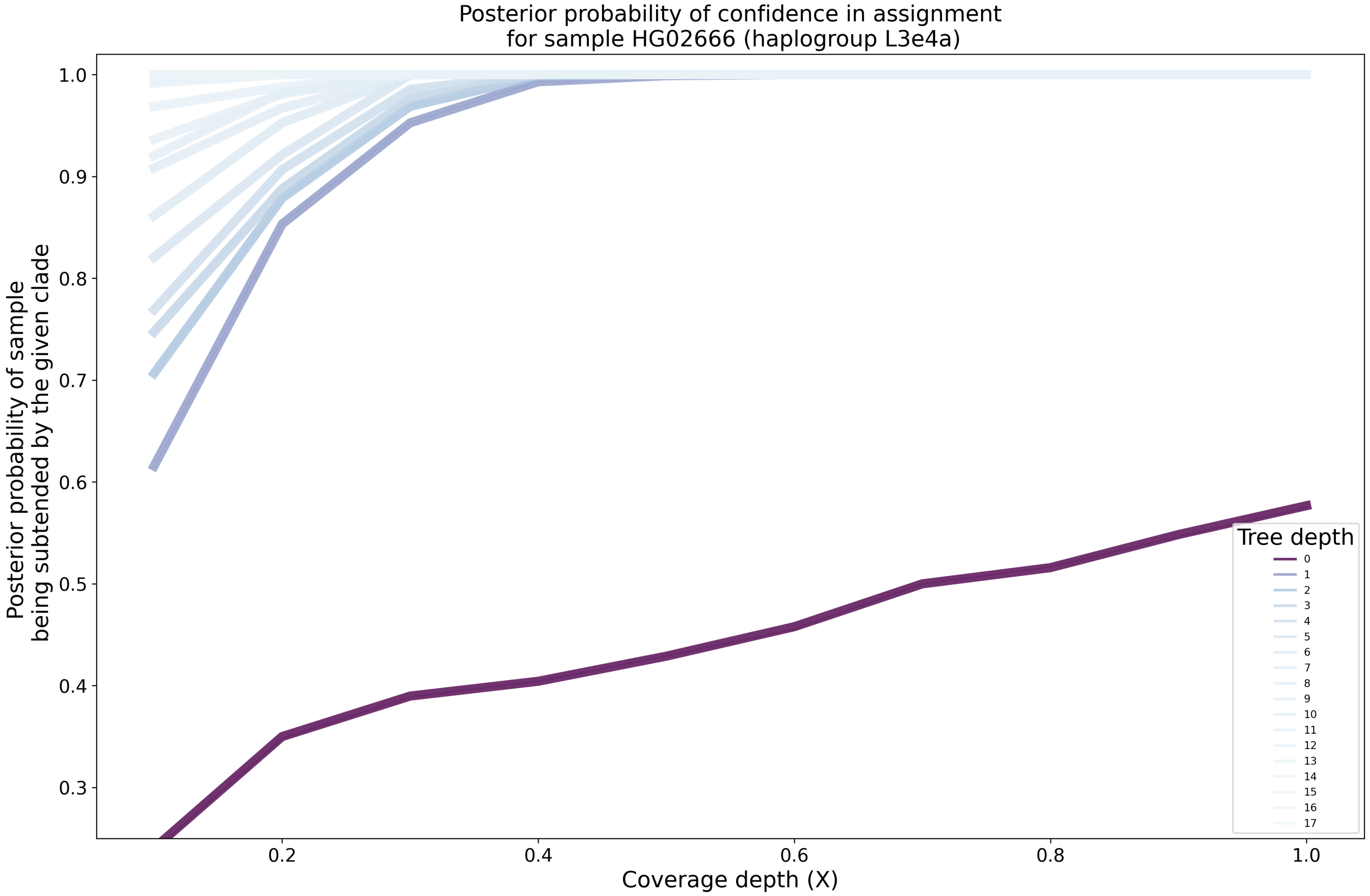

Supplementary Figure BD: **Clade-level posterior probabilities on empirical paired-end FASTQ data.** Each lineplot represents the mean over replicates at a fixed depth on the mitochondrial tree. The darker the line, the more basal the haplogroups.

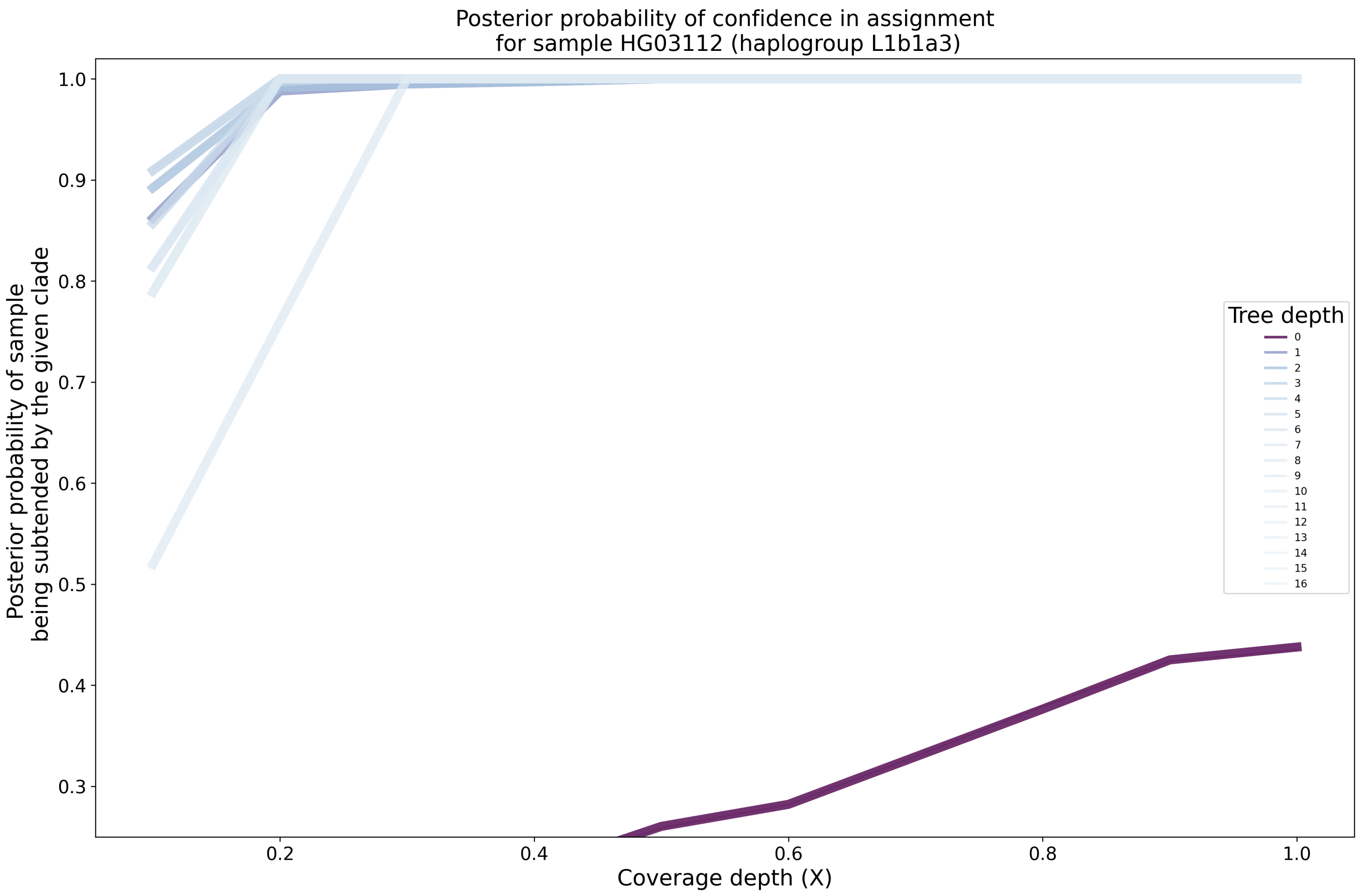

Supplementary Figure BE: **Clade-level posterior probabilities on empirical paired-end FASTQ data (CONTINUED).** Each lineplot represents the mean over replicates at a fixed depth on the mitochondrial tree. The darker the line, the more basal the haplogroups.

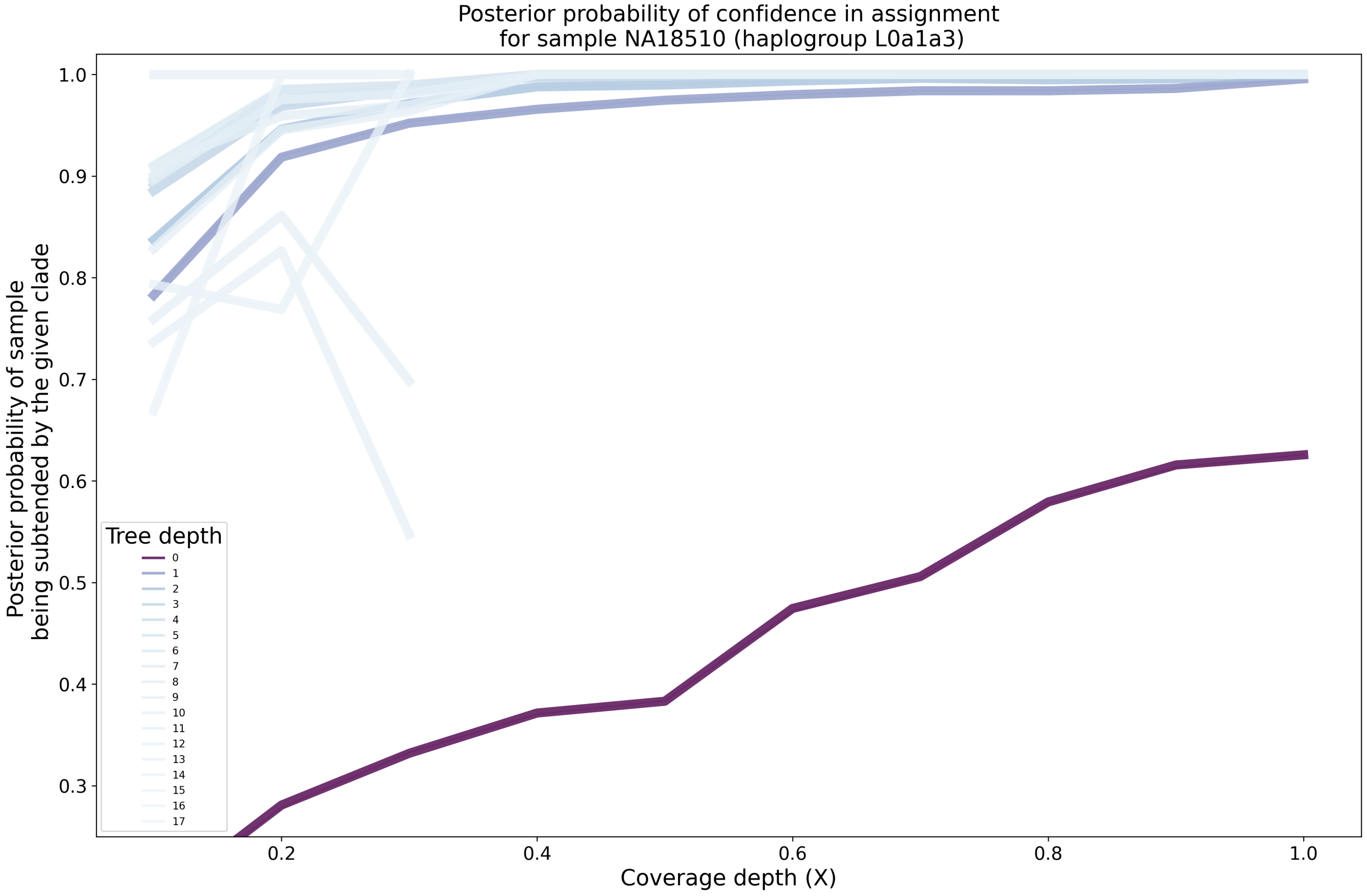

Supplementary Figure BF: **Clade-level posterior probabilities on empirical paired-end FASTQ data.** Each lineplot represents the mean over replicates at a fixed depth on the mitochondrial tree. The darker the line, the more basal the haplogroups.

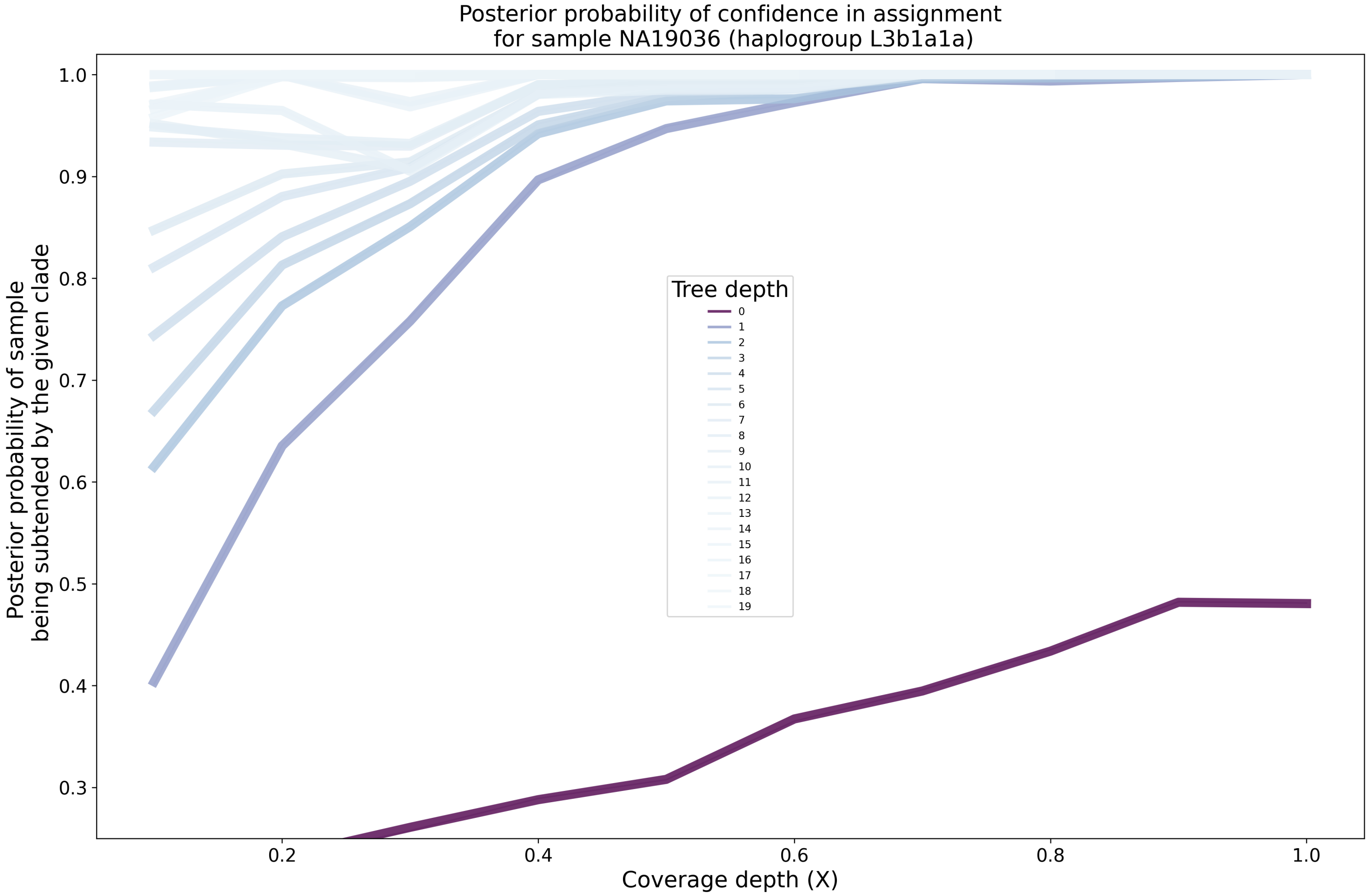

Supplementary Figure BG: **Clade-level posterior probabilities on empirical paired-end FASTQ data (CONTINUED).** Each lineplot represents the mean over replicates at a fixed depth on the mitochondrial tree. The darker the line, the more basal the haplogroups.

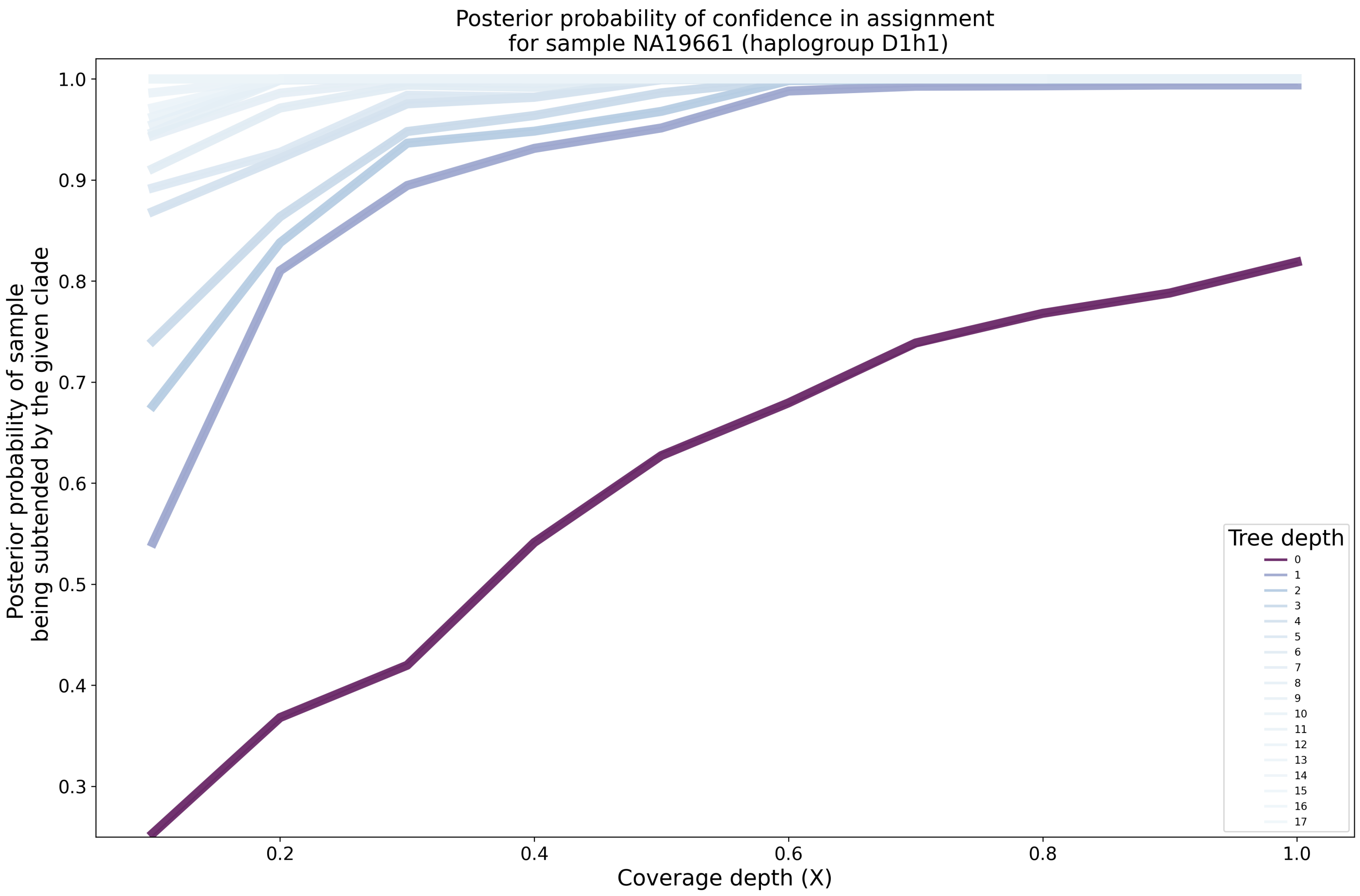

Supplementary Figure BH: **Clade-level posterior probabilities on empirical paired-end FASTQ data.** Each lineplot represents the mean over replicates at a fixed depth on the mitochondrial tree. The darker the line, the more basal the haplogroups.

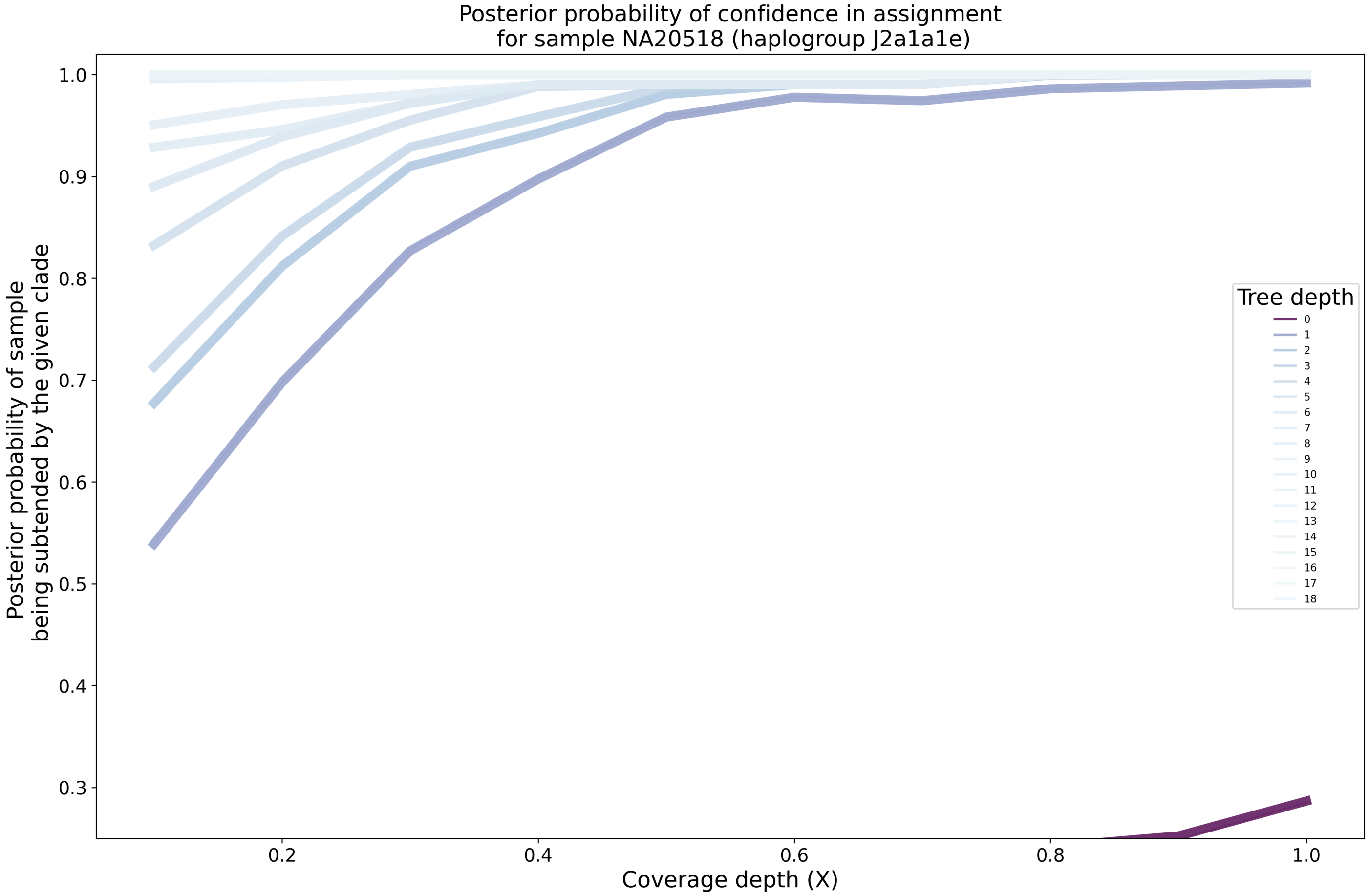

Supplementary Figure BI: **Clade-level posterior probabilities on empirical paired-end FASTQ data (CONTINUED).** Each lineplot represents the mean over replicates at a fixed depth on the mitochondrial tree. The darker the line, the more basal the haplogroups.

2.5 Runtime and Peak Memory Usage

Program	User time (s)	Wall clock time (s)	Peak memory usage (Mb)
`HaploCart`	24.63	28.06	1334
`HaploGrep2`	4.22	1.49	191
`Phy-Mer`	29.30	30.16	579

Supplementary Table D: **Runtime and Peak Memory Usage on FASTA input.** Results are averaged over three input samples (NCBI accessions MZ387838, MW057682, MN894713). Statistics were measured with the command `/usr/bin/time -v`. User time and wall clock time are reported to two significant digits, while peak memory usage is rounded to the nearest integer. In all cases `HaploCart` was run in quiet mode (`-q`) without posterior calculations (`-np`).

Program	User time (s)	Wall clock time (s)	Peak memory usage (Mb)
`HaploCart`	25.40	11.14	1335
`HaploGrep2`	4.22	1.49	191
`Phy-Mer`	29.30	30.16	579

Supplementary Table E: **Runtime and Peak Memory Usage on FASTA input.** Results are averaged over three input samples (NCBI accessions MZ387838, MW057682, MN894713). Statistics were measured with the command `/usr/bin/time -v`. User time and wall clock time are reported to two significant digits, while peak memory usage is rounded to the nearest integer. In all cases `HaploCart` was run in quiet mode (`-q`) without posterior calculations (`-np`).

Program	User time (s)	Wall clock time (s)	Peak memory usage (Mb)
`HaploCart`	25.29	8.03	1335
`HaploGrep2`	4.22	1.49	191
`Phy-Mer`	29.30	30.16	579

Supplementary Table F: **Runtime and Peak Memory Usage on FASTA input.** Results are averaged over three input samples (NCBI accessions MZ387838, MW057682, MN894713). Statistics were measured with the command `/usr/bin/time -v`. User time and wall clock time are reported to two significant digits, while peak memory usage is rounded to the nearest integer. In all cases `HaploCart` was run in quiet mode (`-q`) without posterior calculations (`-np`).

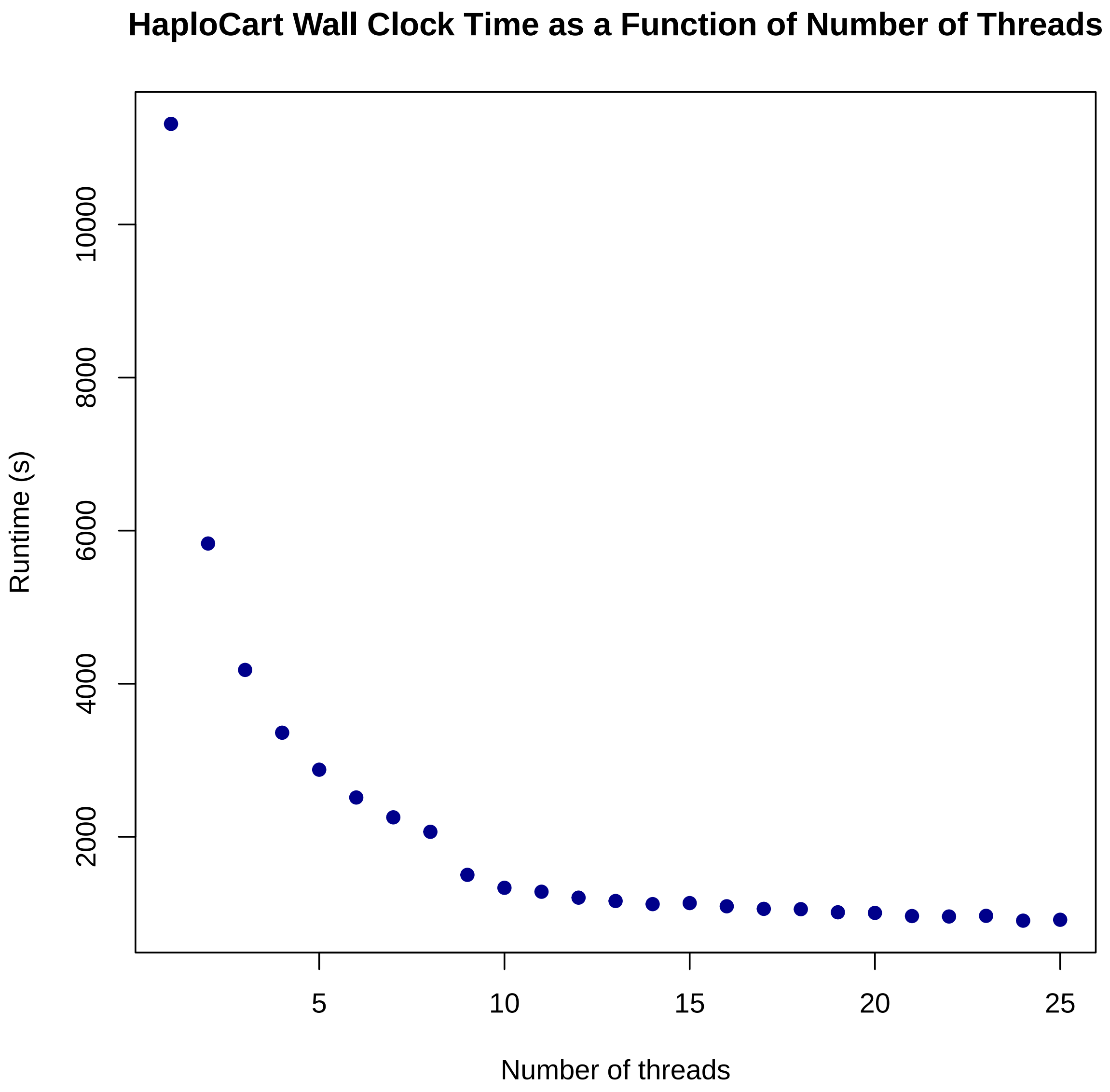

Supplementary Figure BJ: `HaploCart` **Wall clock time (in seconds) as a function of number of threads from one to fifteen.** Each point represents the time taken to report haplogroup assignments on the 311 empirical consensus FASTA sequences detailed in Table SG. `HaploCart` was run in quiet mode (-q) without computing clade-level posterior probabilities of assignments (-np). Wall clock times were measured with the command `/usr/bin/time -v`.

103 ## 2.6 Empirical Data in FASTA Format

**Population**	**NCBI Accession**	`HaploCart` **prediction**	`HaploGrep2` **prediction**	
Australian	AF346963	S1	S1	[**9**]
Australian	AF346964	M42a	M42a	[**9**]
Australian	AF346965	S2	S2	[**9**]
India	AF346966	G3b1	G3b1	[**9**]
Bamileke	AF346967	L3e3b2	L3e3b2	[**9**]
Biaka	AF346968	L1c1a1a1a	L1c1a1a1a	[**9**]
Biaka	AF346969	L1c1a2b	L1c1a2b	[**9**]
Buriat	AF346970	C4b3b	C4b3b	[**9**]
Chukchi	AF346971	A2b1	A2b1	[**9**]
Chinese	AF346972	F4a1b	F4a1b	[**9**]
Chinese	AF346973	M9a1a1a	M9a1a1a	[**9**]
Tatar	AF346974	H5	H5	[**9**]
Dutch	AF346975	H5a1a	H5a1a	[**9**]
Effik	AF346976	L2a1i1	L2a1i1	[**9**]
Effik	AF346977	L2a1a2	L2a1a2	[**9**]
English	AF346978	HV0d	HV0+195	[**9**]
Evenki	AF346979	C4a2a1b	C4a2a1b	[**9**]
Ewondo	AF346980	L3e1e1	L3e1e1	[**9**]
French	AF346981	H1r	H1r	[**9**]
Georgian	AF346982	T2e	T2e	[**9**]
German	AF346983	J1c4	J1c4	[**9**]
Guarani	AF346984	D1a2	D1a2	[**9**]
Hausa	AF346985	L0a1a2	L0a1a2	[**9**]
Ibo	AF346986	L1b1a3	L1b1a3	[**9**]
Ibo	AF346987	L1c1d1	L1c1d1	[**9**]
Italian	AF346988	U5b3a1a	U5b3a1a	[**9**]
Japan	AF346989	D4b2b1	D4b2b1	[**9**]
Japan	AF346990	D4a1	D4a1	[**9**]
Khirgiz	AF346991	B4a1b1a	B4a1b1a	[**9**]
Kikuyu	AF346992	L1c2a1a	L1c2a1a	[**9**]
Korea	AF346993	B4a1b1a	B4a1b1a	[**9**]
Lisongo	AF346994	L3e2b7	L3e2b7	[**9**]
Mandenka	AF346995	L2c3a	L2c3a	[**9**]
Mbenzele	AF346996	L1c1a2b	L1c1a2b	[**9**]
Mbenzele	AF346997	L1c1a1a1a	L1c1a1a1a	[**9**]
Mbuti	AF346998	L0a2b	L0a2b	[**9**]
Mbuti	AF346999	L0a2b	L0a2b	[**9**]
Mkamba	AF347000	L3h1a2a1	L3h1a2a1	[**9**]
Piman	AF347001	B2a5	B2a5	[**9**]
Papua_New_Guinea_Coast	AF347002	P1	P1	[**9**]
Papua_New_Guinea_Coast	AF347003	Q1	Q1	[**9**]
Papua_New_Guinea_High	AF347004	P1d1	P1d1*	[**9**]
Papua_New_Guinea_High	AF347005	P1	P1+152	[**9**]
Saami	AF347006	V7a1	V7a1	[**9**]
Samoa	AF347007	B4a1a1a16	B4a1a1+152	[**9**]
San	AF347008	L0k1a1a	L0k1a1a	[**9**]
San	AF347009	L0k1a1c	L0k1a1c	[**9**]
SibInuit	AF347010	D2a1b	D2a1b	[**9**]
Uzbek	AF347011	B4c2a	B4c2a	[**9**]
Warao	AF347012	C1d1	C1d1*5	[**9**]
Warao	AF347013	C1d1	C1d1*5	[**9**]
Yoruba	AF347014	L3d1a1a	L3d1a1a	[**9**]
Yoruba	AF347015	L3e2b1a1	L3e2b1a1	[**9**]
Mauritania	AF381981	L2c1a	L2c1a*1	[**13**]
Canary	AF381982	U3a1c	U3a1c	[**13**]
Morocco	AF381983	U3a	U3a	[**13**]
Morocco	AF381984	M1a2a	M1a2a*	[**13**]

Morocco	AF381985	T2e1a	T	[**13**]
Morocco	AF381986	X2b2	X2b2	[**13**]
Morocco	AF381987	J1b2	J1b2	[**13**]
Morocco	AF381988	L0a1b1	L0a1b1	[**13**]
Berber	AF381989	U5b1b1	U5b1b1+152	[**13**]
Berber	AF381990	V25	V25	[**13**]
Mauritania	AF381991	L3b1a	L3b1a	[**13**]
Mauritania	AF381992	L1c3a	L1c3a	[**13**]
Mauritania	AF381993	H1	H1	[**13**]
Mauritania	AF381994	L1b1a5	L1b1a5	[**13**]
Jordan	AF381995	U2e	U2e	[**13**]
Jordan	AF381996	M1b1	M1b1	[**13**]
Jordan	AF381997	R	HV+73	[**13**]
Jordan	AF381998	L3d3b	L3d3b	[**13**]
Jordan	AF381999	N1b1a	N1b1a	[**13**]
Jordan	AF382000	U2d2	U2d2	[**13**]
Maragato	AF382001	J1d1b	J1d1b	[**13**]
Maragato	AF382002	H1a	H100	[**13**]
Maragato	AF382003	W1	W1	[**13**]
Leon	AF382004	U2e1c1	U2e1c1	[**13**]
Leon	AF382005	K1b2	K1b2b	[**13**]
Leon	AF382006	T1a1	T1a1	[**13**]
Leon	AF382007	I5a1b	I5a1b	[**13**]
Morocco	AF382008	U6a7a2	U6a7a2	[**13**]
Canary	AF382009	C1b2	C1b2	[**13**]
Canary	AF382010	A2	A2	[**13**]
Andalusia	AF382011	U7b	U7b	[**13**]
Filipino	AF382012	M7c1c3	M7c1c3	[**13**]
India	AF382013	M30c	M30c	[**13**]
Taiwan_Aborigine	AJ842744	B4a1a3a1a	B4a1a3a1a	[**26**]
Taiwan_Aborigine	AJ842745	B4a1a2	B4a1a2	[**26**]
Taiwan_Aborigine	AJ842746	B4a1a	B4a1a	[**26**]
Taiwan_Aborigine	AJ842747	B4a1a4	B4a1a4	[**26**]
Taiwan_Aborigine	AJ842748	B4a1a2	B4a1a2	[**26**]
Taiwan_Aborigine	AJ842749	B4a1a	B4a1a	[**26**]
Taiwan_Aborigine	AJ842750	B4a2a3	B4a2a3	[**26**]
Taiwan_Aborigine	AJ842751	B4a2a1	B4a2a1	[**26**]
Caucasian	AY195745	T2b21	T2b21	[**17**]
Caucasian	AY195746	H3ak	H3ak	[**17**]
Caucasian	AY195747	H5a1	H5a1	[**17**]
Native_American	AY195748	D1	D1	[**17**]
Native_American	AY195749	B2d	B2d	[**17**]
Caucasian	AY195750	V	V	[**17**]
Caucasian	AY195751	H11a7	H11a7	[**17**]
Caucasian	AY195752	H3b1a	H3b1a	[**17**]
Evenki	AY195753	C4a1a3a1	C4a1a3a1	[**17**]
Caucasian	AY195754	J1c1d	J1c1d	[**17**]
Georgian	AY195755	G2a1	G2a1+16189	[**17**]
Georgian	AY195756	N1b1a3	N1b1a3	[**17**]
Iraqi_Israeli	AY195757	H	H	[**17**]
Caucasian	AY195758	H8c2	H8c2	[**17**]
Native_American	AY195759	C1b1	C1b1	[**17**]
Korea	AY195760	A5b1a	A5b1a*	[**17**]
Koryak	AY195761	Z1a2a	Z1a2a	[**17**]
Koryak	AY195762	G1b4	G1b+16129	[**17**]
Koryak	AY195763	C4b2a	C4b2a	[**17**]
Caucasian	AY195764	U2e1f1	U2e1f1	[**17**]
Caucasian	AY195765	K1c1b	K1c1b	[**17**]
South_African	AY195766	L2b1a3	L2b1a3*	[**17**]
Caucasian	AY195767	T2b1	T2b1	[**17**]

Caucasian	AY195768	W3a1	W3a1	[**17**]
Caucasian	AY195769	I1b	I1b	[**17**]
Tofalar_Negidal	AY195770	B4a1c2	B4a1c2	[**17**]
Taiwan	AY195771	A5b1c1	A5b1c1	[**17**]
Udegei	AY195772	C4b1	C4b1	[**17**]
Finland	AY195773	X2c1	X2c1	[**17**]
Caucasian	AY195774	J1c4b	J1c4b	[**17**]
Caucasian	AY195775	H1b1c	H1b1+16362	[**17**]
South_African	AY195776	L2a1f	L2a1f	[**17**]
Khwe	AY195777	L0d2a1a	L0d2a1a	[**17**]
Caucasian	AY195778	J2b1a3	J2b1a3	[**17**]
Finland	AY195779	W1a	W1a	[**17**]
South_African	AY195780	L0a1a2	L0a1a2	[**17**]
Caucasian	AY195781	V6	V6	[**17**]
South_African	AY195782	L3d1d	L3d1d*2	[**17**]
San	AY195783	L1b1a4a	L1b1a4a	[**17**]
South_African	AY195784	L3b1a	L3b1a*4	[**17**]
South_African	AY195785	L2c5	L2c5	[**17**]
Mixteca_Baja	AY195786	A2v1	A2v1	[**17**]
Navajo	AY195787	X2a2	X2a2	[**17**]
San	AY195788	L2a2b1a	L2a2b1a	[**17**]
San	AY195789	L1c1a1a1b1	L1c1a1a1b1	[**17**]
Nanaj_Negidal	AY195790	D4o2a	D4o2a	[**17**]
Malay	AY195791	F1a1a1	F1a1a1	[**17**]
Nanaj_Negidal	AY195792	Y1a	Y1a+16189	[**17**]
Berber	AY275527	U6b	U6b	[**14**]
Canary	AY275528	U6b1a1	U6b1a1	[**14**]
Senegal	AY275529	U6b	U6b	[**14**]
Galicia	AY275530	U6b	U6b	[**14**]
Mauritania	AY275531	U6a7a1	U6a7a1	[**14**]
Maragato_Leon	AY275532	U6a7a1	U6a7a1	[**14**]
Canary	AY275533	U6a7b1	U6a7b1	[**14**]
Morocco	AY275534	U6a1a1	U6a1a1	[**14**]
Mauritania	AY275535	U6a	U6a+16189	[**14**]
Berber	AY275536	U6c2	U6c2	[**14**]
Canary	AY275537	U6c1	U6c1	[**14**]
Australian	AY289051	S2	S2	[**8**]
Australian	AY289052	P3a	P3a	[**8**]
Australian	AY289053	P6	P6	[**8**]
Australian	AY289054	P7	P7	[**8**]
Australian	AY289055	P6	P6	[**8**]
Australian	AY289056	O1a	O1a	[**8**]
Australian	AY289057	P4b1	P4b1	[**8**]
Australian	AY289058	O1a	O1a	[**8**]
Australian	AY289059	O	O	[**8**]
Australian	AY289060	S2	S2*	[**8**]
Australian	AY289061	S2	S2	[**8**]
Australian	AY289062	S4	S4	[**8**]
Australian	AY289063	P5	P5	[**8**]
Australian	AY289064	P4b	P4b	[**8**]
Australian	AY289065	P3a	P3a	[**8**]
Australian	AY289066	S3	S3	[**8**]
Australian	AY289067	S3	S3	[**8**]
Cook	AY289068	B4a1a1m1	B4a1a1m1	[**8**]
Cook	AY289069	B4a1a1a16	B4a1a1a*1	[**8**]
Filipino	AY289070	E2a	E2a	[**8**]
Kannada	AY289071	M30b	M30b	[**8**]
Koraga	AY289072	M30a1	M30a1	[**8**]
Koraga	AY289073	U1a1a	U1a1a	[**8**]
Mullukurun	AY289074	M35a1a	M35a1a	[**8**]

Nasioi	AY289075	Q1c1a	Q1c1a	[**8**]
Papua_New_Guinea_Coast	AY289076	B4a1a	B4a1a	[**8**]
Papua_New_Guinea_Coast	AY289077	B4a1a1	B4a1a1	[**8**]
Papua_New_Guinea_Coast	AY289078	Q3a1	Q3a1*	[**8**]
Papua_New_Guinea_Coast	AY289079	Q3a	Q3a	[**8**]
Papua_New_Guinea_Coast	AY289080	B4a1a1	B4a1a1	[**8**]
Papua_New_Guinea_Coast	AY289081	Q1	Q1	[**8**]
Papua_New_Guinea_Coast	AY289082	Q1a1a	Q1a1a	[**8**]
Papua_New_Guinea_Coast	AY289083	B4a1a1a1	B4a1a1a1	[**8**]
Papua_New_Guinea_High	AY289084	P2	P2*1a	[**8**]
Papua_New_Guinea_High	AY289085	Q1a	Q1a	[**8**]
Papua_New_Guinea_High	AY289086	P1	P1	[**8**]
Papua_New_Guinea_High	AY289087	P1d1	P1d1*	[**8**]
Papua_New_Guinea_High	AY289088	P2	P2*1a	[**8**]
Papua_New_Guinea_High	AY289089	Q3a1	Q3a1*	[**8**]
Papua_New_Guinea_High	AY289090	Q1	Q1	[**8**]
Papua_New_Guinea_High	AY289091	P3b	P3b	[**8**]
Papua_New_Guinea_High	AY289092	P1	P1d	[**8**]
Samoa	AY289093	B4a1a1a13	B4a1a1a13	[**8**]
Samoa	AY289094	B4a1a1k1	B4a1a1k1	[**8**]
Taiwan_Aborigine	AY289095	F4b1	F4b1	[**8**]
Taiwan_Aborigine	AY289096	F4b1	F4b1*	[**8**]
Taiwan_Aborigine	AY289097	M7b1a2a1a	M7b1a2a1a*	[**8**]
Taiwan_Aborigine	AY289098	M7b1a2a1	M7b1a2a1	[**8**]
Thai	AY289099	F1a1a1	F1a1a1	[**8**]
Thai	AY289100	B4c2a	B4c2a	[**8**]
Thai	AY289101	B4c2	B4c2	[**8**]
Tonga	AY289102	B4a1a1a16	B4a1a1	[**8**]
Buriat	AY519484	B4d1'2'3	B4d1'2'3	[**24**]
Evenki	AY519485	C4a2a1b	C4a2a1b	[**24**]
Ket	AY519486	A8a	A8a	[**24**]
Koryak	AY519487	C4b2a	C4b2a	[**24**]
Mansi	AY519488	A12a	A12a	[**24**]
Negidal'tsy	AY519489	B5b2a	B5b2a	[**24**]
Nganasan	AY519490	C4b8a	C4b8a	[**24**]
Nganasan	AY519491	D4o2a	D4o2a	[**24**]
Tofalar	AY519492	B4a1c2	B4a1c2	[**24**]
Tofalar	AY519493	Z1	Z1	[**24**]
Tubalar	AY519494	B4b1a	B4b1a	[**24**]
Tuvan	AY519495	B4a1c2	B4a1c2	[**24**]
Ul'chi	AY519496	C1a	C1a	[**24**]
Ul'chi	AY519497	M8a2b	M8a2b	[**24**]
Mansi	AY570524	D5a3a1a	D5a3a1a	[**24**]
Tuvan	AY570525	D5a2a1	D5a2a1	[**24**]
Tuvli	AY570526	C4b1a	C4b1a	[**24**]
Nganasan	AY615359	C5b1a1	C5b1a1	[**24**]
Tofalar	AY615360	C4a1a3a1	C4a1a3a1	[**24**]
Ul'chi	AY615361	C5a1	C5a1	[**24**]
Pakistan	AY882379	U2a1a	U2a1a*	[**1**]
Pakistan	AY882380	U2b2	U2b2	[**1**]
Pakistan	AY882381	U2c1b	U2c1b*1a	[**1**]
Spain	AY882382	U2e1a1	U2e1a1	[**1**]
Yemen	AY882383	U3a2a1	U3a2a1	[**1**]
Adygei	AY882384	U3b3	U3b3	[**1**]
Yemen	AY882385	U3b1a1	U3b1a1	[**1**]
Spain	AY882386	U4a1a	U4a1a	[**1**]
Italian	AY882387	U4a2a	U4a2a	[**1**]
Adygei	AY882388	U4b1a1a1	U4b1a1a1	[**1**]
Ethiopia	AY882389	U9a	U9a	[**1**]
Pakistan	AY882390	U9b1	U9b1	[**1**]

Pakistan	AY882391	U7b	U7b	[**1**]
Spain	AY882392	U8a1a1	U8a1a1	[**1**]
Italian	AY882393	U8b1a1	U8b1a1*	[**1**]
Italian	AY882394	K1c1a	K1c1a	[**1**]
Italian	AY882395	K1a26	K1a26	[**1**]
Adygei	AY882396	U1a1a	U1a1a+16129*	[**1**]
Italian	AY882397	U1b3	U1b3	[**1**]
Adygei	AY882398	U5a1f1a	U5a1f1a	[**1**]
Italian	AY882399	U5a1	U5a1*	[**1**]
Italian	AY882400	U5b1b1d	U5b1b1+@16192	[**1**]
Spain	AY882401	U5b1b1d	U5b1b1d	[**1**]
Italian	AY882402	U5b1b1d	U5b1b1d	[**1**]
Saami	AY882403	U5b1b1a	U5b1b1a	[**1**]
Saami	AY882404	U5b1b1a1	U5b1b1a1	[**1**]
Yakut	AY882405	U5b1b1a	U5b1b1a	[**1**]
Saami	AY882406	U5b1b1a3	U5b1b1a3	[**1**]
Fulbe	AY882407	U5b1b1b	U5b1b1b	[**1**]
Berber	AY882408	U5b1b1e	U5b1b1e	[**1**]
Italian	AY882409	U5b1c	U5b1c	[**1**]
Italian	AY882410	U5b1c1a1	U5b1c1a1	[**1**]
Italian	AY882411	U5b1d1b	U5b1d1b	[**1**]
Berber	AY882412	U5b1d1a	U5b1d1a	[**1**]
Italian	AY882413	U5b2a1a2	U5b2a1a2	[**1**]
Spain	AY882414	U5b2a1a2	U5b2a1a2	[**1**]
Italian	AY882415	U5b2a2a1	U5b2a2a1	[**1**]
Ethiopia	AY882416	U6a2a2a	U6a2a2a	[**1**]
Spain	AY882417	U6b1	U6b1	[**1**]
Nicobarese	AY950286	B5a1a1	B5a1a1	[**25**]
Nicobarese	AY950287	B5a1a1	B5a1a1	[**25**]
Nicobarese	AY950288	B5a1a1	B5a1a1	[**25**]
Nicobarese	AY950289	F1a1a1	F1a1a1	[**25**]
Nicobarese	AY950290	B5a1a1	B5a1a1	[**25**]
Onge	AY950291	M32a	M32a	[**25**]
Onge	AY950292	M32a	M32a	[**25**]
Onge	AY950293	M31a1a	M31a1a	[**25**]
Onge	AY950294	M31a1a	M31a1a	[**25**]
Onge	AY950295	M32a	M32a	[**25**]
Great_Andamanese	AY950296	M32a	M32a*	[**25**]
Great_Andamanese	AY950297	M31a1a	M31a1a	[**25**]
Great_Andamanese	AY950298	M31a1a	M31a1a	[**25**]
Great_Andamanese	AY950299	M32a	M32a	[**25**]
Great_Andamanese	AY950300	M31a1a	M31a1a	[**25**]
New_Britain	AY956412	Q2a3a	Q2a3a	[**5**]
New_Ireland	AY956413	Q2a	Q2a	[**5**]
New_Britain	AY956414	Q2a	Q2a	[**5**]
Cambodian	AY963572	F1a1a1	F1a1a1	[**15**]
Chinese	AY963573	D4b2b	D4b2b	[**15**]
Bougainville	AY963574	B4a1a1aa	B4a1a1aa	[**15**]
Chinese	AY963575	A6a	A6a	[**15**]
Semang	AY963576	M21a	M21a	[**15**]
Semang	AY963577	M13b1	M13b1	[**15**]
Malay	AY963578	N22a	N22a	[**15**]
Malay	AY963579	R9b1a1a	R9b1a1a*	[**15**]
Malay	AY963580	N21a	N21a	[**15**]
Malay	AY963581	M21b1a	M21b1a*	[**15**]
Meyayu	AY963582	M55	M55	[**15**]
Malayu	AY963583	M22a	M22a	[**15**]
Semang	AY963584	R21	R21	[**15**]
Ugandan	AY963585	L0f2a1	L0f2a1	[**15**]
Italian	AY963586	I3a1	I3a1	[**2**]

New_Britain	DQ137398	M28b	M28b	[**16**]
New_Britain	DQ137399	M28b1	M28b1	[**16**]
New_Britain	DQ137400	M28a7	M28a7	[**16**]
New_Britain	DQ137401	M28a5b	M28a5b	[**16**]
New_Britain	DQ137402	M27b1	M27b1	[**16**]
New_Britain	DQ137403	M27b1	M27b1	[**16**]
New_Britain	DQ137404	M27b1	M27b1	[**16**]
Bougainville	DQ137405	M27c	M27c	[**16**]
New_Ireland	DQ137406	M27c	M27c	[**16**]
New_Britain	DQ137407	M29a	M29a	[**16**]
New_Britain	DQ137408	M29a	M29a	[**16**]
New_Britain	DQ137409	M29a	M29a	[**16**]
Bougainville	DQ137410	M27a1a1	M27a1a1	[**16**]
Bougainville	DQ137411	M27a1a1	M27a1a1	[**16**]

Supplementary Table G: Prediction of `HaploCart` and `HaploGrep2` on 311 human mitogenomes used in various publications including [**6**, **23**, **20**].

104 2.7 Empirical Ancient Data in Consensus FASTA Format

Sample ID	Age	ENA Accession	Unresolved Bases	HaploCart	HaploGrep2	Publication
DA100	1629 ybp	ERR2505844	2361	C4b1	C4b1	[**3**]
DA101	1701 ybp	ERR2505845	1082	U5a1b1e	U5a1b1e	[**3**]
DA171	1900-1300 ybp	ERR2505882	2982	H2a1	H2a1	[**3**]
DA15	2564 ybp	ERR2505876	2601	C4d	C4d	[**3**]
I10899	11700-7500 ybp	ERR3078265	1856	U5b	U5b	[**18**]
I11300	5400-5000 ybp	ERR3078266	2000	J2a1a1	J2a1a1	[**18**]
I8132	4600-4400 ybp	ERR3078350	12983	D1a2	H2a2a1	[**18**]
I8569	4871-4626 ybp	ERR3078345	4212	H1ah	H1ah	[**18**]
I7645	5990-5550 ybp	ERR3078321	11375	R7b2	H2a2a1	[**18**]
I7646	5710-5630 ybp	ERR3078322	8280	H1e1c	H2a2a1	[**18**]
STR393b	1550-1350 ybp	ERR2178282	1155	H5a1	H5a1	[**27**]
NW54	1550 ybp	ERR2178268	662	C4a1a1	C4a1a1	[**27**]
AED204	1550-1200ybp	ERR2178253	808	X2b11	X2b+226	[**27**]
Alh10	1600-1550 ybp	ERR2178259	111	I1	I1	[**27**]
FN2	1300-1600 ybp	ERR2178265	84	H3	H3	[**27**]
AED92b	1550-1200 ybp	ERR2178257	1633	U4a1	U4a1	[**27**]
STR266b	1550-1350 ybp	ERR2178275	1110	J1c	J1c5	[**27**]
Vim2b	1450 ybp	ERR2178288	245	H7	H7	[**27**]
STR491	1550-1350 ybp	ERR2178285	2657	T2b	T2b	[**27**]
STR486	1550-1350 ybp	ERR2178284	964	T2b	T2b	[**27**]

Supplementary Table H: Prediction of `HaploCart` and `HaploGrep2` on consensus FASTA called from ancient BAM files. The raw data was downloaded from the European Nucleotide Archive[**11**] using accessions found from the Ancient mtDNA Database(AmtDB)[**4**]. To call a consensus we ran the command `angsd doFasta 2 -minq 25 -minmapq 25 -uniqueonly 1 -doCounts 1 -seed 42` as per [**21**]. This was called on the original raw BAM file, i.e. not remapped to the mitochondria. The estimates for the age of the samples are in years before present and were found in the supplementary of the original publications.

2.8 Empirical Ancient Data in BAM Format

Sample	Prediction on BAM		Original prediction on consensus	MT Coverage(X)
	HaploCart	HaploGrep2	HaploCart/HaploGrep2	
DA100	C4b1	C4b1	C4b1	83.92
DA101	U5a1b1e	U5a1b1e	U5a1b1e	122.68
DA171	H2a1	H2a1	H2a1	11.61
DA15	C4d	C4d	C4d	50.81
AED204	X2b7	X2b+226	X2b11/X2b+266	11.50
I10899	U5b1	U5b1a	U5b	106.47
I11300	J2a1a1	J2a1a1	J2a1a1	71.31
I8132	H1aq	No pred	D1a2/H2a2a1	1.52
I8569	H1ah	No pred	H1ah/No Pred	6.45
Alh10	I1	I1	I1	183.33
STR393b	H5a1	H5a1	H5a1	12.06
NW54	C4a1a1	C4a1a1	C4a1a1	16.81
FN2	H3	H3	H3	172.58
AED92b	U4a1	U4a1	U4a1	12.29
STR266b	J1c	J1c	J1c/J1c5	8.43
Vim2b	H7	H7	H7	44.58
I7645	HV0d	No pred	R7b2/H2a2a1	1.63
I7646	H1e1c	H1e1c	H1e1c/H2a2a1	2.47
STR491	T2b	T2b	T2b	5.18
STR486	T2b	T2b	T2b	24.96

Supplementary Table I: Prediction of `HaploCart` and `HaploGrep2` on ancient BAM files at full coverage. The prediction on the consensus is provided again for comparison. If there was a disagreement for this predction, a '/' is used to denote the 2 different calls.

2.9 Empirical Ancient Data in BAM Format, Subsampled

Sample	0.5x		1x		2x		Full Coverage	
	HCT	HG2	HCT	HG2	HCT	HG2	HCT	HG2
DA100	C4b1	C	C4b1	C	C4b1	C4	C4b1	C4b1
DA101	U5a1b1e	U	U5a1b1e	U5a'b	U5a1b1e	U5a1b1e	U5a1b1e	U5a1b1e
DA171	H107	H1an2	H36	H2a1	H3i	H2a1	H2a1	H2a1
DA15	C4d	C	C4d	C4d	C4d	C4d	C4d	C4d
AED204	X2b4a1	H2a2a1	X2b4a1	H2a2a1	X2b'd	H2a2a1	X2b7	X2b+226
I10899	U5b1	R0	U5b1	U5b	U5b1	U5b	U5b1	U5b1a
I11300	J2a1a1	J	J2a1a1	J2a1	J2a1a1	J2a1a1	J2a1a1	J2a1a1
I8132	H1aq	No pred	H1aq	No pred	N/A	N/A	H1aq	No pred
I8569	H1e1a7	No pred	H1ah	No pred	H1ah	No pred	H1ah	No Pred
Alh10	I1	N1a1b	I1	I1	I1	I1	I1	I1
STR393b	H5c	H5a1	H10c	H5a1	H5a1	H2a2a	H5a1	H5a1
NW54	C4a1a	H2a2a1	C4a1a	H2a2a1	C4a1a1	C4a1a	C4a1a1	C4a1a1
FN2	H2a1	H2a2a	H3ar	H2a2a	H53	H	H3	H3
AED92b	U4a2d	U5a1a1b	U4a1	H2a2a1	U4a1	U4a1	U4a1	U4a1
STR266b	J1c9	J	J1c9	J	J1c9	J	J1c	J1c
Vim2b	H23	Z1	H23	H2a2a1	H7	H2a12a1	H7	H7
I7645	F	No Pred	HV0d	No Pred	N/A	N/A	HV0d	No Pred
I7646	H1e1c	No Pred	H1e1c	H1bi	H1e1c	H1e1c	H1e1c	H1e1c
STR491	T2b	T2b	T2b33	T2b	T2b	T2b	T2b	T2b
STR486	T2	H2a2a1	T2b	T2b	T2b	T2b	T2b	T2b

Supplementary Table J: Prediction of `HaploCart` and `HaploGrep2` on ancient BAM files at target coverage depths of $0.5X$, $1X$, and $2X$ on the mitochondria. These results suggest that `HaploCart` is able to return more precise predictions on low-coverage ancient data due to its more sensitive mapping and its obviation of reference bias towards the rCRS (which has haplogroup H2a2a1). 'N/A' denotes cases where 2X coverage is greater than the mean coverage of the full sample.

References

1. Alessandro Achilli et al. 'Saami and Berbers—an unexpected mitochondrial DNA link'. In: *The American Journal of Human Genetics* 76.5 (2005), pp. 883–886
2. Hans-Jürgen Bandelt et al. 'Low "penetrance" of phylogenetic knowledge in mitochondrial disease studies'. In: *Biochemical and Biophysical Research Communications* 333.1 (2005), pp. 122–130
3. Peter de Barros Damgaard et al. '137 ancient human genomes from across the Eurasian steppes'. In: *Nature* 557.7705 (2018), pp. 369–374
4. Edvard Ehler et al. 'AmtDB: a database of ancient human mitochondrial genomes'. In: *Nucleic acids research* 47.D1 (2019), pp. D29–D32
5. Jonathan Friedlaender et al. 'Expanding Southwest Pacific mitochondrial haplogroups P and Q'. In: *Molecular Biology and Evolution* 22.6 (2005), pp. 1506–1517
6. Qiaomei Fu et al. 'A revised timescale for human evolution based on ancient mitochondrial genomes'. In: *Current Biology* 23.7 (2013), pp. 553–559
7. *Human NumtS mitochondrial sequence.* https://genome.ucsc.edu/cgi-bin/hgTrackUi?hgsid=1369590759_fnmZlGFaIgECQhbAaziAwj8CC8Q8&db=hg18&c=chr5&g=numtSeq. Accessed: 2022-09-03
8. Max Ingman and Ulf Gyllensten. 'Mitochondrial genome variation and evolutionary history of Australian and New Guinean aborigines'. In: *Genome Research* 13.7 (2003), pp. 1600–1606
9. Max Ingman et al. 'Mitochondrial genome variation and the origin of modern humans'. In: *Nature* 408.6813 (2000), pp. 708–713
10. Daniela Lascaro et al. 'The RHNumtS compilation: features and bioinformatics approaches to locate and quantify Human NumtS'. In: *BMC Genomics* 9.1 (2008), pp. 1–13
11. Rasko Leinonen et al. 'The European nucleotide archive'. In: *Nucleic acids research* 39.suppl_1 (2010), pp. D28–D31
12. Heng Li. 'Minimap2: pairwise alignment for nucleotide sequences'. In: *Bioinformatics* 34.18 (2018), pp. 3094–3100
13. Nicole Maca-Meyer et al. 'Major genomic mitochondrial lineages delineate early human expansions'. In: *BMC Genetics* 2.1 (2001), pp. 1–8
14. Nicole Maca-Meyer et al. 'Mitochondrial DNA transit between West Asia and North Africa inferred from U6 phylogeography'. In: *BMC Genetics* 4.1 (2003), pp. 1–11
15. Vincent Macaulay et al. 'Single, rapid coastal settlement of Asia revealed by analysis of complete mitochondrial genomes'. In: *Science* 308.5724 (2005), pp. 1034–1036
16. D Andrew Merriwether et al. 'Ancient mitochondrial M haplogroups identified in the Southwest Pacific'. In: *Proceedings of the National Academy of Sciences* 102.37 (2005), pp. 13034–13039
17. Dan Mishmar et al. 'Natural selection shaped regional mtDNA variation in humans'. In: *Proceedings of the National Academy of Sciences* 100.1 (2003), pp. 171–176
18. Iñigo Olalde et al. 'The genomic history of the Iberian Peninsula over the past 8000 years'. In: *Science* 363.6432 (2019), pp. 1230–1234
19. Christopher Pockrandt et al. 'GenMap: ultra-fast computation of genome mappability'. In: *Bioinformatics* 36.12 (2020), pp. 3687–3692
20. Cosimo Posth et al. 'Pleistocene mitochondrial genomes suggest a single major dispersal of non-Africans and a Late Glacial population turnover in Europe'. In: *Current Biology* 26.6 (2016), pp. 827–833
21. Aparna Prasad, Eline D Lorenzen, and Michael V Westbury. 'Evaluating the role of reference-genome phylogenetic distance on evolutionary inference'. In: *Molecular Ecology Resources* 22.1 (2022), pp. 45–55
22. Domenico Simone et al. 'The reference human nuclear mitochondrial sequences compilation validated and implemented on the UCSC genome browser'. In: *BMC Genomics* 12.1 (2011), pp. 1–11
23. Pontus Skoglund et al. 'Separating endogenous ancient DNA from modern day contamination in a Siberian Neandertal'. In: *Proceedings of the National Academy of Sciences* 111.6 (2014), pp. 2229–2234
24. Elena B Starikovskaya et al. 'Mitochondrial DNA diversity in indigenous populations of the southern extent of Siberia, and the origins of Native American haplogroups'. In: *Annals of Human Genetics* 69.1 (2005), pp. 67–89
25. Kumarasamy Thangaraj et al. 'Reconstructing the origin of Andaman Islanders'. In: *Science* 308.5724 (2005), pp. 996–996
26. Jean A Trejaut et al. 'Traces of archaic mitochondrial lineages persist in Austronesian-speaking Formosan populations'. In: *PLOS Biology* 3.8 (2005), e247
27. Krishna R veeramah2018 et al. 'Population genomic analysis of elongated skulls reveals extensive female-biased immigration in Early Medieval Bavaria'. In: *Proceedings of the National Academy of Sciences* 115.13 (2018), pp. 3494–3499
28. *vg::Alignment Struct Reference.* https://vgteam.github.io/vg/structvg_1_1Alignment.html. Accessed: 2022-09-03
